# Supplementary material for: Malaysia’s Health Systems Response to COVID-19
Source: Int J Environ Res Public Health. 2021 Oct 22;18(21):11109. doi: 10.3390/ijerph182111109 (PMC8583455; doi:10.3390/ijerph182111109)
Supplement: Supplementary file 1 [file ijerph-18-11109-s001.zip › ijerph-1346571-supplementary/Supplemental Files/Supplementary File S1 - Evidence Table.pdf]

Appendix 6 Evidence table for relevant contents in the paper

References	Date of article / report / webpage / event	Details / Extracts of response
Ministry of Health Malaysia[1]	03/01/2020	It has begun to prepare for any possible transmission of COVID-19 to Malaysia since 3 January 2020 again where reagents to detect coronavirus with using the Conventional RT-PCR method has been prepared. Next, on January 11, 2020 when scientists from China have sharing genetic information COVID-19, on the same day, IMR Virology Unit has they created a sequence of reagents known as "primers and probes specific for COVID-19. The protocol that has been used by this IMR is also in line with the real-time RT-PCR protocol COVID-19 which has been released by the WHO according to Corman et al., 2020.
Ministry of Health Malaysia[2]	04/01/2020	Strengthening of screening activities for travellers from China at all international entries. 64 entries throughout Malaysia comprised of airports, seaports and ground-crossings.<br>54 thermal scanner at main international entrances. Fever screening and history of travelling to China within 2 weeks prior to arrival in Malaysia were taken. Those who fulfil PUI criteria will be referred to hospital for further management. Those who didn't fulfil PUI criteria were given Home Assessment Tools and put under Home Surveillance for 14 days. Those without symptoms were given Health Alert Card<br>-Random screening for Malaysian who used autogate for immigration clearance
Ministry of Health Malaysia [3]	06/01/2020	Infection and prevention control<br>Use of PPE for all staff involved in managing suspected cases.<br>Collaboration (national)<br>MOH will continuously collaborate closely with all related agencies to strengthen preparedness and response activities to face potential infection spread to Malaysia.<br>MOH will continuously collaborate closely with all related agencies to strengthen preparedness and response activities to face potential infection spread to Malaysia<br>Updated that China authority clarified that COVID-19 virus was not the same with avian flu, MER-CoV or SARS. Clarified that WHO did not issue any advice on travel and trading restriction with China<br>Strengthening existing fever screening activities among travellers by members of the PoE.<br>In the event of any feverish and symptomatic travellers, further examination would be conducted at the health quarantine centre or checkpoint located at the PoE<br>Information on the occurrence of these unknown pneumonia clusters in Wuhan, China and the prevention and control measures for infection among MOH members at all levels including the points of entry. In the event of any feverish and symptomatic travelers, check-ups will be conducted at the health quarantine center or checkpoint located at the PoE.<br>Strengthening screening of suspected patients at public primary care facilities and hospital emergency departments, and adhering to IPC strategies when managing cases.<br>Use existing notifying and reporting procedure. Immediately report to district health office, state health department

		and CPRC if detect any suspected cases. This applied for all HCWs from both public and private sectors.<br>Strengthening SARI and ILI surveillance at all identified sentinel location.<br>Continuously monitor ILI cluster in community and unusual events such as admission to ICU or death among ILI patients<br>MOH monitored current situation continuously
Minister of Health Malaysia [4]	16/01/2020	All MOH staff include health officers at PoE were informed about COVID-19 cluster in Hubei, China and the infection prevention and control measures<br>mphasise and strengthen existing temperature screening by MOH staff at PoEs<br>If a traveller was detected fever by thermal scanner, examination will be performed again at the health quarantine center or examination center located at the international PoE.<br>Travellers who had respiratory tract infections within 14 days after returning home were advised to visit nearby health facilities for examination.
Minister of Health Malaysia [5]	17/01/2020	Risk communication<br>Director General of Health of the Malaysian Ministry of Health released a circular to alert all state health directors and relevant ministry directors about the emergence of the novel coronavirus. The letter provides the details of the preparedness and response, which was summarised in Govdoc_1.
Ministry of Health Malaysia [6]	21/01/2020	The detection reagents of COVID-19 virus arrived at IMR laboratory. At the same time, University Malaya Medical Centre (UMMC) shared RNA extracts obtained from an isolated killed virus that belongs to the same family as COVID-19. The RNA extracts were to be utilized as positive control for the COVID-19 tests.<br>Laboratory officers from IMR successfully optimized the Real-Time RT-PCR for COVID-19 virus detection<br>Institute of Medical Research (IMR) was the sole laboratory involved in conducting diagnostic tests by setting up reagents using coventional RT-PCR method to detect COVID-19 virus<br>IMR sent the optimized reagents to NPHL to enable successful identification of the first confirmed case of 2019-nCoV among close contacts<br>IMR identified the first positive case among patients under investigation (PUI)<br>IMR conducted training on the Real-Time RT-PCR for detection of COVID-19 to 12 other MOH hospital laboratories located in different states namely NPHL Sungai Buloh, PHL Ipoh, PHL Kota Kinabalu, PHL Kota Bharu and PHL Johor Bahru<br>Received samples were processed immediately and was subjected to Real-time RT-PCR for COVID-19 virus. This was to ensure the results could be released within 24 hours even though at most time, the results were released much earlier.<br>As a precaution step to ensure the safety of laboratory and health officers, only those whom undergone competency tests and trained on the appropriate technique of wearing personal protective equipment (PPE) were allowed to conduct tests. Received samples were recorded in the logbook and then be taken to Biosafety Level 2 (BSL 2) laboratory. The samples were processed in small aliquoted volume which will be subjected to inactivation and proceeding with Real-Time RT-PCR COVID-19 testing. The officer on-call is responsible in releasing the result to requesting hospital.<br>Scientists from China shared the genomic information of the virus via a shared database which enabled the virologists in IMR to design reagent sequences such as primers and probes to detect COVID-19 virus.

Ministry of Health Malaysia [7]	23/01/2020	Rapid response team<br>Standby with Rapid Response Team (RRT) and Rapid Assessment Team (RAT) at district level to conduct contact tracing<br>MOH had issued standard operating procedures to all government and private health facilities as references for healthcare staff at ground level to detect, handle and management individuals suspected of COVID-19<br>The MOH has strengthened its health screening activities at all International PoEs, and any suspected travellers referred for further inspection;<br>MOH collaborated with other relevant agencies at International PoEs to enable rapid tracking of travellers with symptoms<br>The use of personal protective equipment (PPE) for all healthcare personnel was emphasised<br>MOH press statement attached photos which showed that MOH carried out training on proper use of PPE, and training on screening of individuals suspected of COVID-19 and routine operation at PoE.<br>Encourage Malaysians who plan to visit China to delay their trip unless the trip could not be avoided.<br>Strengthening the screening activities at primary healthcare and emergency trauma department<br>26 MOH hospitals for managing suspected cases<br>Use existing notifying and reporting procedure. Immediately report to district health office, state health department and CPRC if detect any suspected cases. This applied for all HCWs from both public and private sectors.<br>Laboratory test result for the first 3 suspected individual were tested negative - zero reporting surveillance<br>MOH were monitored current situation continuously<br>IMR and National Public Health (NPHL) Sungai Buloh were the designated labs ready to receive samples for detection COVID-19 virus
Ministry of Health Malaysia [8]	24/01/2020	International collaboration<br>Collaboration and health information sharing between Ministry of Health Singapore and MOH Malaysia. MOH will continue this two-way collaboration.<br>Rapid Response Team<br>JKN Johor conducted contract tracing, did health screening and took clinical samples of contacts.<br>JKN took fast action to tract contact soon after Singapore notified those individuals were resided in Johor Bahru and had contacted with their confirmed cases.<br>JKN monitored those individuals for 14 days or until their departure to their country of origin, whichever came first.<br>No cases were detected by JKN - zero-case reporting
Ministry of Health Malaysia [9]	25/01/2020	We have initiated thermal screenings (since Jan 1 at the airports and from Jan 22 at the borders) to isolate travellers with symptoms of the illness.
[10]	25/01/2020	Reported first 4 imported cases COVID-19 cases<br>Encourage Malaysians who plan to visit China to delay their trip unless the trip could not be avoided.<br>Encourage Malaysians who travel to China to practice several prevention measures:<br>a) Practice high levels of personal hygiene as often as possible by wash hands with water and soap or use hand sanitizer.<br>b) to bring along and use face mask and hand sanitiser during travel.

		c) Avoid visiting public places and do not closely contact with any of those individuals<br>d) Avoid visiting animal farms, markets selling live animals, animal slaughterhouses or touching any kind of animal;present symptoms;<br>e) Avoid consuming any raw animal product / not cooked properly.<br>f) Get medical attention right away if not feeling well (ie, have respiratory tract infections such as fever, cough or difficulty breathing) within 14 days after returning home<br>JKN did contact tracing under same travel agencies after first case detected in Malaysia and conduct health screening for them<br>MOH monitored current situation continuously
Loheswar[11]	25/01/2020	Financing<br>Funding for outbreak management - MOH will work with MOF and Ministry of Economic Affairs
Ministry of Health Malaysia [12]	26/01/2020	DG press statement described latest news, details and actions taken on COVID-19 cases in Malaysia.<br>Encourage Malaysians who plan to visit China to delay their trip unless the trip could not be avoided.<br>Encourage Malaysians who travel to China to practice several prevention measures:<br>a) Practice high levels of personal hygiene as often as possible by wash hands with water and soap or use hand sanitizer.<br>b) to bring along and use face mask and hand sanitiser during travel.<br>c) Avoid visiting public places and do not closely contact with any of those individuals<br>d) Avoid visiting animal farms, markets selling live animals, animal slaughterhouses or touching any kind of animal;present symptoms;<br>e) Avoid consuming any raw animal product / not cooked properly.<br>f) Get medical attention immediately if not feeling well (ie, have respiratory tract infections such as fever, cough or difficulty breathing) within 14 days after returning home<br>MOH were monitored current situation continuously<br>Collaboration<br>MOH is collaborating with various agencies including PDRM to detect suspected and confirmed cases so isolation can be carried out.<br>Regulation / Law<br>Enforcement of Akta 342: Collaboration with police in detaining a Chinese couple in Johor, who had evaded quarantine orders for their child who is suspected of having 2019-nCoV<br>Regulation / Law<br>Enforcement of Seksyen 14 Akta Pencegahan dan Pengawalan Penyakit Berjangkit 1988 (Akta 342) where PUIs are to be quarantined for 14 days from exposure date or departure from China.
Thomas[13]	26/01/2020	Wan Azizah, who chaired a high-level emergency meeting in her capacity as the head of the National Disaster Management Agency (Nadma) here today, said various local and international agencies are cooperating to stem the spread of the outbreak in Malaysia.
Palansamy[14]	26/01/2020	MOH would meet with National Disaster Management Agency and Home Ministry to discuss on intensifying COVID-19 measures.

Yusof and Landau[15]	26/01/2020	Co-ordination<br>Cluster Novel Coronavirus Coordination Meeting held, chaired by Deputy Prime Minister.<br>The ministry, he said, had requested for 12 additional thermal scanners to strengthen health screening at all entry points.
Immigration Department of Malaysia[16]	27/01/2020	Immigration Malaysia released press which stated to restrict entry for holders for all type of entry visas from Wuhan City, Hubei, China following Coronavirus outbreak in the region.
New Straits Times[17]	27/01/2020	Preparedness<br>Hotels in Johor prepares for managing impending coronavirus outbreak risk by implementing standard operating procedures for quarantine and communication, as well as providing masks and hand sanitisers.
Ministry of Health Malaysia [2]	28/01/2020	MOH to collaborate with Immigration Department of Malaysia to refer travellers from China to carry further assessment by health officers at PoE<br>The travel agency is required to inform customers who intend go to China about the situation the latest outbreak of COVID-19 outbreaks and travel advice issued by MOH from time to time are:<br>1) Advise the public to avoid visiting China until further notice.<br>b) Practice high levels of personal hygiene as often as possible by wash hands with water and soap or use hand sanitizer.<br>c) If a visit to China is inevitable, while there:<br>- Avoid visiting public places and do not closely contact with any of those individuals<br>Avoid visiting animal farms, markets selling live animals, animal slaughterhouses or touching any kind of animal; present symptoms;<br>d) Avoid consuming any raw animal product / not cooked properly.<br>e) To medical attention immediately if not feeling well (ie, have respiratory tract infections such as fever, cough or difficulty breathing) within 14 days after returning home<br>Empowering agencies and tourism associations for create a fever screening on travellers and refer to them to the nearest health facility for further examination and treatment.<br>DG press statement described preparedness and response at PoE for COVID-19<br>To tackle COVID-19, MOH increased its screening to all travelers (passengers and crew) from China at all the PoEs (64 international airport, seaports and land entrances in Malaysia).<br>54 thermal scanners currently installed at various PoEs<br>Any passengers were detected to have fever would be assessed paramedics to confirm the symptom, If the passengers with fever had history of travel to China for past 14 days would be treated as PUI and referred to designated hospital for treatment by a special ambulance accompanied by a trained personnel equipped with PPE<br>Passengers who were not classified as PUI would be issued Home Assessment Tool and to serve Home Surveillance for a period of 14 days.Those with cough but without fever will be given a face mask.<br>Asymptomatic travellers would be given Health Alert Card and advised to seek medical attention if they have fever, cough or shortness of breath within 14 days from arrival and bring together the card when getting treatment.<br>Certain PoEs would carry out periodical announcement which advised travellers to go to health assessment office for

		assessment.<br>MOH officers would randomly screen Malaysians traveller who used autogate to cross border.<br>MOH collaborated with Malaysia Airport Berhad to divert all flight from China a designated gate to prevent mixing of passengers from China and passengers from other countries.<br>MOH encourage all Malaysians voluntarily undergo health assessment at PoEs.<br>Passengers from China would undergo temperature screening and those classified as PUI would be referred to hospital using an assigned pathway to prevent exposure to other passengers<br>All ships / cruise from China would be quarantined until MOH officer has carried out examination onboard. Unloading activities and disembarkation of passengers and crews would only be allowed if all passengers and crews are healthy and sanitisation certificate are valid.<br>Airline operators are requsted to identify passengers with symptoms onboard. Health officer would be informed to standby at predetermined gate. Once the flight arrives at the gate, the officer would go onboard and handle the passengers with symptoms.<br>- Travel advisory to travellers from China<br>- Travel agencies to inform travellers from China to declare their health status before arrival to Malaysia. Procedure to undergo monitoring and quarantine -orders including isolation if symptomatic should be explained to clients.<br>- Travellers from China to declare health status to travel agencies before arrival to Malaysia.<br>Empowering agencies and tourism associations for create a fever screening on travelers and refer to them to the nearest health facility for further examination and treatment.<br>Self-declaration for all travellers from China prior entering Malaysia
Ong[18]	28/01/2020	The Ministry of Finance will be expediting the additional allocation for 12 thermal scanners as stated by Minister of Health Datuk Seri Dr Dzulkefly Ahmad.<br>In a statement today, minister Lim Guan Eng said the thermal scanners are to strengthen the capabilities of Ministry of Health staff in identifying individuals with 2019-nCoV (Novel Coronavirus) symptoms at the country's points of entry.<br>Considering that the application is related to public health protection efforts and critical in nature, I have requested the Secretary-General of the Treasury Tan Sri Ahmad Badri Mohd Zahir to give full cooperation so that the allocation can be channelled out as soon as possible.
Ministry of Health Malaysia[19]	29/01/2020	On January 28, 2020, a meeting with the Ministry of Tourism, Arts and Culture Malaysia was held. The meeting has agreed to assist with:<br>a) To provide travel advice issued by MOH to tourists from China;<br>b) Inform travellers from China about health screening requirements at the country's international gateway;<br>c) Remind travellers to maintain a good personal hygiene such as washing their hands with water and soap or using hand sanitizer.<br>d) Advise Malaysians who visit China to refrain from visiting crowded places, do not closely contact with any symptomatic individuals, avoid visits to animal farms, markets selling live animals, slaughterhouse or touching animals of any kind; and avoid eating raw or partially cook animal products<br>To acquire asset at PoE in order to increase screening capacity<br>To update COVID-19 PUI management guideline for the reference of all health front liners

		Epidemiological report for number of cases among PUI and close contact<br><br>Increase number of public health labs and hospital labs for virus testing<br>Monitoring health statis of HCWs who involved managing confirmed and suspected cases<br>Increasing the number of laboratory that is able to conduct COVID-19 virus detection tests by providing trainings and reagents. Monitoring the performance and health of staff involved<br>Supply control and management<br>- Stockpiling of PPE to ensure enough supply for frontline healthcare workers<br>- Ensure medicine supply for treatment of symptoms and complication of 2019-nCoV cases is at optimum level<br>Human resource management<br>Monitor health status of healthcare staff involved in management of 2019-nCoV patients and PUIs<br>Guidelines (clinical)<br>Update guidelines for PUI and 2019-nCoV infection management for medical practitioners' reference
Mok[20]	29/01/2020	Strategies announced by Penang Chief Minister<br>- Health authorities are already conducting strict health checks on tourists arriving at the Penang International Airport (PIA) and Swettenham Pier.<br>- Those who arrived from China will be screened and monitored over a 14-day period through home surveillance system
Palansamy [21]	29/01/2020	International collaboration<br>Prime Minister announced that Malaysia will be discussing with China to airlift Malaysian citizens trapped in the Hubei province back home and those found to be ill will be quarantined before allowed home
Tawie[22]	29/01/2020	Regulation / Law<br>Enforcement of Section 233 of the Communications and Multimedia Commission Act 1998, to be read together with Section 505 of the Penal Code in Sarawak (following alleged fake news on coronavirus patient in a private hospital in Kuching)
Tan [23]	29/01/2020	Johor - Kukup International Ferry Terminal started health screening at entry point
Ministry of Health Malaysia[24]	29/01/2020	Regulation / Law<br>Fees Act<br>- Exemption of all medical charges for non-citizens for symptomatic covid-19 patients and close contacts to positive cases<br>- RM40 for walk in covid-19 testing of asymptomatic non-citizens
Ministry of Health Malaysia[25]	30/01/2020	Co-ordination / Collaboration<br>Special committee for Humanitarian mission was activated, co-ordinated by National Disaster Management Agency (NADMA), Ministry of Foreign Affairs and MOH, in collaboration with AirAsia and Malaysian Airlines to bring back Malaysians from Hubei, China<br>Collaboration<br>Collaboration with Ministry of Education to trace students and lecturers returning from holidays in China. Those

		identified will be on home surveillance and given Health Advice Card and Home Assessment Tool. Those with symptoms will be referred to the health facilities immediately
[26]	30/01/2020	- Apart from that, MoH said it is working closely with the Education Ministry to identify students, teachers and lecturers who are returning from China and those identified will be placed under home surveillance and issued health warning cards as well as home assessment tools.<br>Advise the public to avoid visiting China.<br>Malaysian visiting China to:<br>a) practice high levels of personal hygiene as often as possible by wash hands with water and soap.<br>b) To medical attention immediately if not feeling well (ie, have respiratory tract infections such as fever, cough or difficulty breathing) within 14 days after returning home<br>MOH collaborated with several ministries to bring back Malaysians who work in Wuhan and were monitored for their health for 14 days.<br>Use existing notifying and reporting procedure. Immediately report to district health office, state health department and CPRC if detect any suspected cases. This applied for all HCWs from both public and private sectors.<br>Dzulkefly also said there are plans to add an additional 25 thermal scanners at entry points to Malaysia so authorities can scan everyone who passes through the entry points. Currently, only Chinese nationals are being screened.<br>“We are planning to make sure all entry points, be it land or sea, are checked. We have 61 entry points in Malaysia, and we are getting 25 additional thermal-scanning units. Besides that, we also have laser thermometers or temperature guns that can also scan people for fever.
Ministry of Health Malaysia[25]	30/01/2020	MoH is working closely with special committee for Humanitarian Assistance and Disaster Relief (HADR) mission was activated, coordinated by National Disaster Management Agency (NADMA), Ministry of Foreign Affairs and MOH, in collaboration with AirAsia and Malaysian Airlines to bring 78 Malaysians working in Wuhan.
[27]	30/01/2020	Infection and prevention control<br>Surfaces that come into regular contact with hands, including escalator handrails, trolley handles and signages, were seen being thoroughly disinfected by the cleaners (at KLIA) in a bid to reduce the risk of transmitting the virus.
Arumugan and Yusof[28]	30/01/2020	Infection and prevention control / Health advisory<br>Deputy Health Minister advised schools to ensure there were hand washing facilities and provide hand sanitisers to students and teachers, told there is no need of restricting students and teachers returning from China from attending classes as there is evidence of novel coronavirus incidents in the community.
Ministry of Health Malaysia[29]	30/01/2020	Advise the public to avoid visiting China.<br>Malaysian visiting China to<br>a) practice high levels of personal hygiene as often as possible by wash hands with water and soap.<br>b) To medical attention immediately if not feeling well (ie, have respiratory tract infections such as fever, cough or difficulty breathing) within 14 days after returning home<br>MOH collaborated with several ministries to bring back Malaysians who work in Wuhan and were monitored for their health for 14 days<br>Use existing notifying and reporting procedure. Immediately report to districh health office, state health department

		and CPRC if detect any suspected cases. This applied for all HCWs from both public and private sectors.<br>MOH monitored current situation continuously
Ministry of Health Malaysia[30]	31/01/2020	Measures by other government agencies<br>- MOE decides to postpone registration of students from Wuhan and Hubei Province at all Malaysian education institutions to a date that will be decided later<br>- Students from other Chinese provinces are allowed to register for studies in Malaysia with close monitoring of the educational institutions involved<br>- All teaching and administrative staff including students, both citizens and non-citizens currently in Malaysia with history of travel to Wuhan or Hubei province will be placed under home quarantine for 14 days from the day of departure.<br>Collaboration<br>MOH will continue to collaborate with several agencies including Pengurusan Bencana Negara (NADMA), Jabatan Imigresen Malaysia (JIM), Polis DiRaja Malaysia (PDRM) dan Kementerian Pendidikan Malaysia (KPM)<br>All educators, staffs and students who had been to Wuhan and Hubei were put under home surveillance to minitors for 14 days from date of departure<br>MOH collaborated with several agencies i.e NADMA, Jabatan Imigresen Malaysia and MOE for early detection and contact tracing<br>Infoline CPRC were made available for further enquiries and advices
Tan[31]	31/01/2020	Regulation / Law<br>Authorities have arrested several individuals for spreading fake news about the 2019-nCoV outbreak here.
Povera[32]	31/01/2020	International collaboration<br>- The Health Sector of Asean, together with counterparts from China, Japan and South Korea said that it had mobilised regional cooperation mechanisms to respond to the rapidly-evolving threat of the coronavirus since the first cluster of unexplained pneumonia cases in late Dec 2019 was reported<br>- ASEAN Emergency Operations Centre Network for public health emergencies (ASEAN EOC Network), led by Malaysia, and with the support of the Asean Secretariat, have been sharing daily situational updates including prevention, detection and response measures to Asean Senior Officials for Health Development (SOMHD) of Asean and China, Japan and South Korea (Plus Three Countries); as well as to Contact Points of the ASEAN EOC Network and the ASEAN Plus Three Field Epidemiology Training Network (Asean+3 FETN)"
Ministry of Health Malaysia[33]	01/02/2020	The Ministry of Health (MOH) notes that the World Health Organization (WHO) has convened a second International Health Regulations (IHR) Emergency Committee meeting on January 30, 2020 on the 2019 novel coronavirus (2019-nCoV) outbreak in China with the spread of other countries. It follows the significant increase in the number of 2019-nCoV cases globally as well as the increasing number of countries reporting positive 2019-nCoV cases. Based on the current developments of the epidemic and the outcome of the Second Meeting, the WHO Director General has agreed to declare the 2019-nCoV outbreak as Public Health Emergency of International Concern (PHEIC).<br>With this PHEIC declaration, some of the temporary recommendations issued by the WHO to member states are to strengthen surveillance, early detection, case management and isolation, close contact management and prevention of 2019-nCoV infection. WHO also emphasizes the importance of Member States to share the latest information on 2019-

		nCoV infections with WHO. For information, the preparedness and response measures currently being implemented by the MOH are in line with WHO recommendations. In addition to the existing preparedness and response plan, the MOH also continues to enhance its preparedness to address unforeseen circumstances while working with all relevant agencies.<br>MOH has been informed of a recent spread on social media about a suspected 2019-nCoV patient who refused to be placed under the Monitoring and Observation Order at a hospital in Selangor. Through investigations, the patient is a Malaysian citizen and has a history of visiting Wuhan, China. After the hospital filed a police report, the suspected patient was successfully identified and placed in a ward for investigation and treatment. Results of clinical samples taken on January 31, 2020, reported negative 2019-nCoV. The MOH would like to take this opportunity to thank the Royal Malaysian Police for the cooperation that has been given in detecting this suspected patient.
Ministry of Health Malaysia [34]	03/02/2020	For information, the Humanitarian Aid Mission is led by the National Disaster Management Agency (NADMA) in collaboration with the Ministry of Foreign Affairs (KLN), Ministry of Health Malaysia (MOH), Ministry of Home Affairs (KDN), AirAsia, Malaysia Airport Berhad, Department of Social Welfare (JKM), the 2nd Malaysia Fire and Rescue Department (JBPM), the Royal Malaysian Police (PDRM), the Malaysian Immigration Department (JIM) and other agencies. In an effort to bring Malaysians who are now in Wuhan, China to their homeland - the MOH will ensure that prevention and infection control measures are fully complied with at all times in carrying out this mission. The mission was divided into four (4) phases, before departing for Wuhan, during the mission of returning home, arriving in the homeland and subsequently the entry of all individuals involved in the identified Monitoring Center. The only ones to be taken home are those who have been healthy after the exit screening that will be performed by the China National Health Authority at the Wuhan Tianhe International Airport. Those with symptoms will be taken to a health facility in Wuhan for further treatment.<br>For Malaysians who were brought by Humanitarian Assistance and Disaster Relief (HADR) mission, after the aircraft carrying them landing at the Kuala Lumpur International Airport (KLIA), all passengers will not enter the KLIA terminal building directly, instead after disembarkation, all passengers would directly board a special bus that would take them to Air Disaster Unit building for decontamination and entry screening. Those who were found to have symptoms would be take designated Quarantine Monitoring Centres. All passengers were only allowed to return home after being confirmed healthy and found to be negative for COVID-19 infection.<br>Epidemiological report for number of cases among PUI and close contact<br>Infoline CPRC were made available for further enquiries and advices
Ministry of Health Malaysia[35]	05/02/2020	"Humanitarian Assistance and Disaster Relief (HADR) mission A total of 107 individuals (88 Malaysians and 19 non-Malaysian family members) were evacuated from Wuhan, involving 26 mission officers (6 medical personnel, 1 from the National Disaster Management Agency (NADMA), 1 from Ministry of Foreign Affairs, 12 crew members and 6 officers from the Malaysian embassy in China). They all underwent the 2019-nCoV infection screening process and clinical samples were obtained. As mentioned earlier, two (2) Malaysians were confirmed 2019-nCoV cases, whilst the remaining 131 individuals tested negative. They are all currently under a 14-day quarantine and observation."<br>Laboratory capacity Initially, laboratory testing to detect and confirm 2019-nCoV infections were limited to the Institute for Medical Research (IMR) and the National Public Health Laboratory (MKAK) Sungai Buloh. Currently a total of 12 hospital laboratories and four (4) public health laboratories throughout Malaysia have capacity, following training by

		IMR using the protocol set out by the World Health Organization. The testing capacity will be further extended to include private laboratory networks, that will be undergoing training organised by the MKAK Sungai Buloh on 6 February 2020.
Ministry of Health Malaysia[36]	06/02/2020	Infection and prevention control<br>- MOH advises those celebrating Thaipusam to take precautionary steps by maintaining hygiene by frequent handwashing with soap of sanitiser and don face masks<br>- Medical Operation Centre co-ordinated by Selayang Hospital will carry our activities to prevent and prepare for any potential incidents
Ministry of Health Malaysia[37]	07/02/2020	Health advisory<br>The public especially those with high risk (children, elderly, pregnant women and those with chronic diseases and impaired immunity) are advised to limit visits to hospitals for non-essential purposes. Those who need to visit the hospital are advised to:<br>- avoid touching eyes, nose and mouth without prior handwashing<br>- wash hands with water and soap for 20 seconds frequently or use a hand sanitiser<br>- avoid those with respiratory symptoms such as cough and runny nose<br>- practice cough etiquette<br>- those with respiratory symptoms should wear a face mask and maintain safe distance (at least 1 meter) with others.
Ministry of Health Malaysia[38]	08/02/2020	Health advisory / Infection and prevention control<br>Recommendation for people who need to use mask (adopted from WHO)<br>- People with respiratory symptoms, e.g. cough or difficulty breathing. Including when seeking medical attention;<br>- People providing care to individuals with respiratory symptoms;<br>- Health workers, when entering a room with patients or treating an individual with respiratory symptoms<br>Advice on the use of mask<br>- Should cover nose and mouth<br>- Perform hand hygiene action thoroughly once touching the mask<br>- Proper disposal also requires a specific procedure to prevent contamination<br>- Should change mask per outing<br>General advice for public<br>- Practice proper hand hygiene with an alcohol-based hand rub or soap and water<br>- Practice respiratory hygiene when coughing and sneezing<br>- Maintain social distancing of at least 1 metre particularly if you are coughing, sneezing and have a fever<br>- Avoid touching your face (eyes, nose and mouth)<br>- Avoid consumption of raw or undercooked animal products<br>- Keep distance from those who are sick<br>- Stay indoors if you are sick<br>Advice on the use of masks in the community

Ministry of Health Malaysia[39]	09/02/2020	Contact tracing were continuously conducted.
Abas[40]	09/02/2020	Authorities have inspected 2,103 wholesale and retail premises selling face masks for the past two weeks starting from Jan 25. Domestic Trade and Consumer Affairs Minister Datuk Seri Saifuddin Nasution Ismail said the daily checks were done in the wake of increased demand for face masks following the 2019 novel coronavirus (2019-nCoV) outbreak. As of Sunday (Feb 9), he said the ministry's enforcement officers had inspected 2,103 business premises, and out of the total, 17 offences were detected with a compound value of RM57,400 and a seizure worth RM4,255.50. "Of the 17 offences, 14 were cases of sale above the maximum price and three were cases of no price display. "I want to emphasise that face mask is a regulated price item under the Price Control (Controlled Prices) Order 2009 and the Price Control (Maximum Pricing) (No.2) Order 2009. "This means anyone who sells face masks exceeding the gazetted price can be penalised under the Price Control and Anti-Profiteering Act 2011," he said in a statement today.
Ministry of Health Malaysia[41]	10/02/2020	International collaboration<br>Establishment of collaborative committee with Singaporean MOH, led by Deputy Health Minister of Malaysia and Singapore (via video-conference) to strengthen various cross-border efforts with focus on information sharing, screening and case management across borders and standardisation of indication for antiviral use for positive covid-19 patients
Ministry of Health Malaysia[42]	10/02/2020	Health advisory<br>Travellers from China that developed symptoms within 14 days of arrival to inform healthcare provider of travel history.<br>Infoline CPRC were made available for further enquiries and advices
Ministry of Health Malaysia[43]	12/02/2020	Official Name for the New Coronavirus Disease On 11 February 2020, the World Health Organization (WHO) has officially named 2019-nCoV infection as COVID-19. "CO" – corona "VI" – virus "D" – disease "19" – the year that the disease was first reported i.e. 31 December 2019 This official name was chosen to avoid any stigma associated with a geographical location, an animal, an individual or group of people, while still being pronounceable and related to the disease.
Ministry of Health Malaysia [44]	12/02/2020	Strengthening the surveillance among SARI<br>Strengthening the surveillance among ILI<br>Strengthening of surveillance activities among local community using existing surveillance system for SARI and ILI at all identified hospitals and Public Community Clinic after 2 out 10 positive imported cases were Malaysian<br>Epidemiological report of daily cases
Perimbanayagam [45]	12/02/2020	1. "He said as of today, the cluster episode has been well-managed by the National Disaster Management Agency (Nadma) chaired by Deputy Prime Minister Datuk Seri Dr Wan Azizah Wan Ismail. "The fact that Nadma has not passed the baton to the Prime Minister showed that it's yet to become a national crisis, (which warrants for Tun Dr Mahathir Mohamad's intervention). So it is not a crisis yet and Nadma is managing it well, rest assured," he said in a press conference at the ministry, here, today. Also present were Deputy Health Minister Lee Boon Chye, Health director-general Datuk Dr Noor Hisham Abdullah and deputy Health director-general (Public Health) Datuk Dr Chong Chee Kheong. Dr Dzulkefly said the ministry is currently working to equip more laboratories with the coronovirus testing

		capability/facilities. "We now have 18 government hospital laboratories and 5 private laboratories equipped with the facilities to carry out the test nationwide," he said."
Ministry of Health Malaysia [46]	13/02/2020	MOH monitored current situation continuously<br>Infoline CPRC were made available for further enquiries and advices
Ministry of Health Malaysia [47]	13/02/2020	MOH has also mobilized the Mental Health and Psychosocial Support Team (MHPSS) since February 6, 2020 following the Humanitarian Aid Mission to bring back Malaysians and their families from Wuhan, China. The team provides psychosocial support services to individuals placed under the Monitoring Order, to MOH Officers and other agencies involved. Activities include group and individual psychological first aid (PFA) sessions, as well as counselling and referral to specialists as needed. Currently, two (2) cases have been referred for further treatment due to acute stress and anxiety. They have been given medication and are in stable condition.
Bernama[48]	14/02/2020	MOH had 80 thermal scanners installed at POEs
National Disaster Management Agency [49]	16/02/2020	Westerdam Cruise Ship (after rejected from docking in several countries), docked in Sihanoukville, Cambodia on 13 February 2020.Operator of the Westerdam Cruise, had arranged with Malaysia Airlines for special charter flights to transport some of these passengers from Sihanouk International Airport, Cambodia, to various final destinations through Kuala Lumpur International Airport (KLIA).Based on their original planning, MAS will arrange for four special charter flights from 14 February until 16 February 2020. • As of today, only one special charter flight (MH8763) carrying 145 passengers has landed in KLIA on 14 February 2020, 7 pm. • All 145 passengers underwent health screening upon disembarkation and two (2) passengers (US nationals; husband and wife) were found to be symptomatic. They were brought to Sungai Buloh Hospital for further investigation. • Subsequent test results confirmed COVID-19 for the 83 years old female on 15 February 2020. The patient suffered from a cough but no fever or difficulty breathing. Her chest X-ray showed signs of pneumonia. • The test results for her husband is negative for COVID-19. He is receiving treatment and being monitored in Sungai Buloh Hospital for his symptoms. • MOH has informed MAS on this confirmed COVID-19 case (Case 22). • As a result, all passengers on that flight was considered as close contact. Based on this latest development, MAS has made the decision to cancel the remaining special charter flights. Repeated COVID-19 tests were conducted on the elderly couple late last night and similar results were obtained, positive for the wife and negative for her husband. • Of the remaining 143 passengers of the flight who passed the thermal screening and were asymptomatic, 137 have continued their journey with outbound flights, while 6 are still awaiting their outbound flights. CENTRAL DISASTER MANAGEMENT COMMITTEE - Minister of Health, Director General of Health, Secretary General of the Ministry of Foreign Affairs, Secretary General of Home Affairs, Secretary General of Ministry of Transport, Director General of the Immigration Department, the National Security Council and the National Disaster Management Agency (NADMA) meeting with Deputy Prime Minister, together with Malaysia Airlines Berhad (MAS) to discuss the current status of COVID-19 cluster and related issues.<br>Following the confirmed one (1) COVID-19 case amongst the passengers of Westerdam cruise ship that docked in Cambodia on 13 February 2020, and subsequent flight arrival in KLIA on 14 February 2020, contact tracing for close contacts activities were conducted immediately. This included formal communications between the Malaysian International Health Regulations (IHR) Focal Point with counterparts from all countries involved. Through these

		communications, information of each passenger was conveyed to the National IHR Focal Points of each respective country. This information will be used by the Ministries of Health of these countries to follow up and trace these passengers once they arrive at their final destinations. 4 In particular, communications with the National IHR Focal Point of Cambodia was also conducted. This is to enable more effective management and prevention activities be implemented at their level – being the country where the cruise ship had docked.<br>Communications with the National IHR Focal Point of Cambodia was also done, whereby the Malaysian IHR Focal Point had communicated with the Cambodian IHR Focal Point since 14 February 2020, even before the positive case was confirmed. This evening I had a teleconference with His Excellency Prof. Mam Bunheng, Minister of Health Cambodia to further strengthen collaboration between two countries in combating COVID-19. I informed His Excellency regarding the improving health status of the US national patient. I have also informed His Excellency that the patient underwent repeated COVID-19 tests, and still found to be positive.<br>Government of Malaysia has made the following decisions: We will not allow any cruise ships originating or transiting through any seaports in China from entering Malaysia.<br>All Westerdam cruise ship passengers still remaining in Cambodia will not be allowed entry into Malaysia, taking into account that they are close contacts with a confirmed COVID-19 case (Case 22).<br>The six (6) remaining passengers who were awaiting their outbound flight, will undergo COVID-29 testing. If the results are negative, they will be allowed for their outward journey. However, if found to be positive, they will be brought to the hospital for further management.
Ministry of Health Malaysia[50]	18/02/2020	MOH wishes to inform that all 107 Malaysians and family members who were evacuated from Wuhan, China through the Humanitarian Assistance and Disaster Relief (HADR) mission on 4 February 2020 will be allowed to return back to their respective homes once they have completed the health monitoring period at 2 the Higher Education Leadership Academy (Akademi Kepimpinan Pendidikan Tinggi – AKEPT). • All repeated COVID-19 tests were found to be negative.
Ministry of Health Malaysia[51]	19/02/2020	Launching of Virtual Health Advisory • The Virtual Health Advisory is a digital channel that was created in collaboration between the Ministry of Health (MOH) and DoctorOnCall (a telemedicine platform). • This is a corporate social responsibility (CSR) initiative by DoctorOnCall that enables the general public to access the Virtual Health Advisory program for free for the first three months. • The Virtual Health Advisory is a program to channel information and reduce public misinformation regarding COVID-19 infection. • Through this platform, the general public will also be able to obtain virtual or online consultation with MOH's Family Medicine Specialists and medical officers. • The virtual consultation is available between 8.30 am to 5.00 pm daily. This service is accessible through www.moh.gov.my using any devices with cameras, including mobile phones, tablets, laptops and personal computers.
Ministry of Health Malaysia[52]	20/02/2020	DIAMOND PRINCESS CRUISE SHIP • The Ministry of Health (MOH) has been updated by the Malaysian Embassy in Tokyo, Japan, on the current status of the four (4) Malaysians on the Diamond Princess cruise ship. o Three (3) passengers o One (1) crew • This cruise ship has undergone quarantine at one of the seaports in Japan since 4 February 2020, following the confirmation of COVID-19 infections amongst the passengers. • Two (2) of the Malaysians, a husband (71 years old) and wife (66 years old) were confirmed positive with COVID-19 on 16 February 2020. They are currently receiving treatment at Fujita Hospital in Nagoya and in stable condition. • The other Malaysian passenger, a 61 years old female, was found to be negative for COVID-19 infection and has been allowed to travel back home to

		Malaysia by the Japanese health authority upon completion of the 14-day quarantine period. • The Malaysian crew member is currently being monitored onboard by Japanese health authority.
Ministry of Health Malaysia[53]	22/02/2020	Repeated laboratory confirmatory tests for COVID-19 will be done to all confirmed COVID-19 case once patients show improvement and signs of recovery from their symptoms.<br>This is necessary to ensure that the cases have fully recovered and free from the COVID-19 virus. Only then will the patients be allowed home with no risk of infecting other individuals.
Ministry of Health Malaysia[54]	23/02/2020	The cruise ship's medical representatives also praised the action the government brought home a Malaysian citizen, and inform the health screening process that has been carried out organized and coordinated so well and smoothly. MoH wishes to congratulate and thank all involved agencies such as the Johor State Secretary's Office, Police Royal Malaysia, Ministry of Foreign Affairs, Ministry of Transport, National Security Council, INTAN, Royal Malaysian Customs Department, Department of Immigration Malaysia, Marine Department of Malaysia, Fire Department and Malaysian Rescue, Malaysian Civil Defense Force, Department National Volunteers, and the SMART Team, coordinated by the National Disaster Management Agency (NADMA) in ensuring the screening process was going well.<br>Observation and Surveillance Order was an order under Section 15(1) Prevention and Control of Infectious Diseases Act 1988 (Act 342)<br>Government of Malaysia had coordinated the docking of World Dream cruise which carried 100 Malaysian crews in Tanjung Pengelih, Johor. All of the crew members were screened on 22 February 2020 before being brought toTadbiran Awam Negara (INTAN) Kampus Wilayah Selatan (IKWAS), Kluang, Johor for sampling and surveillance. The results were back on 23 February with all samples tested negative. All crew members were subsequently allowed to return home and serve Observation and Surveillance Order for 14 days.
Ministry of Health Malaysia [55]	23/02/2020	To identify COVID-19 infection transmission in the community in Malaysia, MOH has earlier since 2 February 2020 started sampling from patients with Influenza Like Illness (ILI) and Severe Acute Respiratory Infection (SARI) in MOH hospitals and health clinics, as one of the COVID-19 infection surveillance activities.<br>Samples for COVID-19 testing are taken from all patients with pneumonia. To date, there were 56 positive cases out of 2,669 samples taken (i.e. 2.1% of total SARI cases sampled).<br>ILI sampling began in the 9th epidemiological week (23 February 2020), starting with eight selected MOH health clinics (as the sentinel sites). This is to determine if there is community spread of the infection.<br>A total of eight (8) confirmed COVID-19 cases were identified from 1,280 samples taken (representing 0.6% of total ILI cases sampled). This surveillance activity serves as an early detection and early warning systems for COVID-19 infection in Malaysia. MOH will continue to expand and strengthen COVID-19 surveillance activities in Malaysia.
[35]	25/02/2020	As of February 23, 2020, there were 7,617,964 travellers screened at International PoE nationwide. Of that total, 27 people were referred to the hospital and two (2) COVID-19 positive cases were the 20th and the 22nd case detected fever through thermal scanner during screening at PoE.
Ministry of Foreign Affairs Malaysia [56]	26/02/2020	Announcement of Ummah Suspension to the Malaysian by Embassy of Malaysia in Riyadh and Consulate General of Malaysia in Jeddah

Ministry of Health Malaysia	26/02/2020	Screening centres involved MOH and non-MOH facilities including Klinik Kesihatan opened during office hours. A list of facilities were also operated and put on standby during weekends with most of the screening were arranged by appoinment.
Ministry of Health Malaysia [57]	26/02/2020	case definition of PUI, confirmed case and close contact
Ministry of Health Malaysia[58]	26/02/2020	patient who come to the respective health facilities should be screened for suspected covid-19 at triage using case definition for PUI
Ministry of Health Malaysia [59]	26/02/2020	Patient who are referred to screening centre at Emergency Trauma Department are sent for Screening Purpose
Ministry of Health Malaysia [60]	26/02/2020	The First Meeting of the Malaysia – Singapore Joint Working Group (JWG) for the Coronavirus Disease (COVID-19). Malaysia and Singapore have agreed on the Terms of Reference (ToR) for the JWG to further enhance cooperation to prevent and control the spread of COVID-19. The JWG will be co-led by the deputy health ministers of both countries. The first JWG meeting held in Johor Bahru yesterday was attended by senior government officials from Malaysia and Singapore. YBhg. Dato' Dr. Chong Chee Kheong, Deputy Director General of Health (Public Health) from the Ministry of Health Malaysia and Dr Benjamin Koh, Deputy Secretary (Development) from the Ministry of Health Singapore led their respective delegations. At the meeting, both countries at the technical level, updated each other on the COVID-19 situations in their respective countries, shared existing screening protocols at the two land borders, and began discussion on joint-border cooperation. Delegates also visited the health screening facilities at Sultan Iskandar Customs, Immigration and Quarantine (CIQ) Complex in Johor Bahru. The JWG agreed to continue entry screening at both countries and align health screening protocols at the land borders; exchange information on clinical management of patients between technical experts; activate a bilateral Field Epidemiology Training Network (FETN) to share surveillance data; and exchange information such as national advisories and public messaging on COVID-19. Both countries reaffirmed the importance of working closely together against the on-going outbreak under the newly formed JWG. The second meeting of the JWG is planned in March 2020.
Ministry of Health Malaysia [61]	26/02/2020	Total screening hospitals - 59 MOH and 1 non-MOH; total admitting hospitals - 26 MOH and 1 non-MOH
Bernama[62]	26/02/2020	The special flight carrying Malaysian nationals from Wuhan, China, the epicentre of the Covid-19 outbreak, arrived safely at KL International Airport (KLIA) early on Wednesday (Feb 26). The AirAsia flight chartered specifically for the Humanitarian Assistance and Disaster Relief (HADR) mission initiated by the Malaysian Government landed at 6.50am. The aircraft left for Wuhan from KLIA2 at 5.58pm on Tuesday (Feb 25).
Ministry of Health Malaysia[63]	27/02/2020	2nd Humanitarian Assistance and Disaster Relief (HADR) Mission - A total of 66 individuals (46 Malaysian citizens and 20 non-Malaysian citizens or their children) were deported from Wuhan, China via the 2nd Humanitarian Assistance Mission on February 26, 2020. During their arrival in Malaysia, they underwent a health screening and sample clinical

		studies were performed for COVID-19 (entry screening). As a result of the screening, no individuals were symptomatic and laboratory results for all 66 individuals were found to be COVID-19 negative. They were then placed in a monitoring centre for a 14-day health monitoring.<br>MOH advised Malaysians to postpone travel to the territories or cities declared as COVID-19 outbreaks in China and also to the Republic of Korea, Japan, Italy and Iran.<br>Health advisory for the travellers abroad and after returning to Malaysia:<br>a) Practice high levels of personal hygiene as often as possible by wash hands with water and soap or use hand sanitizer.<br>b) Always practice good cough etiquette, that is to cover mouth and nose with tissue when coughing or sneezing sneeze, then wash hands immediately;<br>c) to bring along and use face mask and hand sanitiser during travel.<br>d) Avoid visiting public places and do not closely contact with any of those individuals<br>e) Avoid visiting animal farms, markets selling live animals, animal slaughterhouses or touching any kind of animal; present symptoms;<br>f) Avoid consuming any raw animal product / not cooked properly.<br>g) Get medical attention right away if not feeling well (ie, have respiratory tract infections such as fever, cough or difficulty breathing) within 14 days after returning home<br>66 individuals brought back from Wuhan. All were screened for symptoms and samples taken were negative for COVID-19. They will be placed and monitored at a quarantine centre for 14 days.
Ministry of Health Malaysia[64]	28/02/2020	Point of entry surveillance / Collaboration (national)<br>Jawatankuasa Kerja Kluster COVID-19 Bersama Pelbagai Agensi meeting:<br>- Temporary entry restriction for visitors from South Korea (Daegu, Cheongdo) including those who have been there within 14 days before arrival to Malaysia and any visitors who will transit through Malaysia<br>- Malaysians, PRs and pass holders who have been at Daegu and Cheongdo within 14 days before arrival in Malaysia has to undergo health screening by KKM.<br>- Special immigration counters for visitors from South Korea, Japan, Italy and Iran<br>To strengthen screening at International Points of Entry, the Malaysian Immigration Department has set up special counters for travellers from the Republic of Korea, Japan, Italy and Iran<br>Malaysians are advised to postpone travel to 3 regions and cities that have been declared with COVID-19 outbreaks in the People's Republic of China (as notified earlier) as well as to the Republic of Korea, Japan, Italy and Iran<br>The public is also advised to inform travel history to the treating doctor when seeking treatment if they develop symptoms of respiratory tract infection within 14 days upon returning from travel to China, Korea, Japan, Italy and Iran<br>Technical Working Committee for COVID-19 Cluster Meeting with other Agencies - The Technical Working Committee for COVID-19 Cluster Meeting with other Agencies was coordinated by the National Disaster Management Agency (NADMA), involving MOH, Ministry of Home Affairs, Malaysian Immigration Department, Ministry of Foreign Affairs and other related agencies

Ministry of Health Malaysia[65]	29/02/2020	With reference to the Press Statement by the Director General of Health Malaysia dated 28 February 2020:<br>1. For Republic of Korea nationals, the travel ban is only applicable to those from Daegu city and Cheongdo county only. However, for Republic of Korea nationals who have left Daegu city and Cheongdo country over 14 days to the date of arrival to Malaysia, there is no travel ban;<br>2. The travel ban also applies to all other foreign nationals if they had travelled to Daegu city and Cheongdo country within 14 days to the date of arrival to Malaysia. However, if they had left Daegu city and Cheongdo county over 14 days to the date of arrival to Malaysia, there is no travel ban;<br>3. For Malaysians, permanent residents and pass holders (long-term social visit and student pass holders) who were in Daegu city and Cheongdo county within the past 14 days, there is no travel ban. However, they will have to undergo health screening as determined by MOH; and<br>MOH again would like to remind the public to postpone non-essential travel to the People's Republic of China, Republic of Korea, Japan, Italy and Iran;<br>1. If the journey cannot be postponed, to ensure that all preventive measures are taken, including maintaining a high-level of personal hygiene at all times;<br>2. Constantly practice good personal hygiene – wash hands frequently with soap and water or use hand sanitizers;<br>3. Constantly practice good cough etiquette – cover mouth and nose with tissue when coughing or sneezing, dispose tissue appropriately after use and wash hands immediately; and<br>4. Seek immediate medical treatment if unwell (with respiratory tract symptoms e.g. fever, cough or difficulty breathing) within 14 days upon return. The treating doctor must be informed of the travel history.<br>2nd Humanitarian Assistance and Disaster Relief (HADR) Mission A total of 66 individuals (Malaysians and non-Malaysian family members) were brought back from Wuhan, China on 25 February 2020. They are currently placed at the Higher Education Leadership Academy (Akademi Kepimpinan Pendidikan Tinggi – AKEPT) and are in good health. The results for health screening through COVID-19 testing upon arrival were all negative. They will continue to stay at AKEPT for health monitoring for 14 days starting from 26 February 2020 until 11 March 2020.
Ministry of Health Malaysia[66]	01/03/2020	Collaboration (national)<br>Meeting on the Management of Suspected and Confirmed COVID-19 Cases in Private Hospitals and Clinics was held at the national level.<br>The main objective of this meeting was to update the private medical practitioners on the latest COVID-19 situation globally and in Malaysia. It was also to ensure a high level of preparedness and response of the private medical practitioners in the face of uncertainties of the current COVID-19 situation.<br>based on the current COVID-19 situation in Malaysia and the latest development globally, all healthcare personnel at all levels and types of healthcare facilities - The mechanism for early detection and isolation of cases must also be strengthened<br>Infoline CPRC were made available for further enquiries and advices<br>Infection and prevention control<br>All healthcare personnel at all levels and types of healthcare facilities are reminded to implement optimal infection prevention and control measures at all times.<br>a Meeting on the Management of Suspected and Confirmed COVID-19 Cases in Private Hospitals and Clinics was held at

		the national level. The main objective of this meeting was to update the private medical practitioners on the latest COVID-19 situation globally and in Malaysia. It was also to ensure a high level of preparedness and response of the private medical practitioners in the face of uncertainties of the current COVID-19 situation.
[67]	01/03/2020	drive-thru testing (testing without getting out of the vehicle), swab & go testing (testing at screening centres) are provided
Prime Minister' Office of Malaysia[68]	03/03/2020	The Prime Minister then held a joint discussion Chief Secretary of State, Secretary General of the Ministry of Foreign Affairs, Chairman Director of National Disaster Management Agency (NADMA), Director General of MOH, Director General of the National Security Council and Director General Immigration Department on the current status of the novel coronavirus outbreak.<br>Malaysians who had visited countries at risk such as China, South Korea, Japan and Italy must comply with the travel and health advisories issued by MOH and immediately go to the hospital / clinic if developed symptoms like cough, fever and breathing difficulties
Ministry of Health Malaysia[69]	04/03/2020	Communication channels<br>MOH would like to inform that for queries on COVID-19, the public can refer to the MOH website www.moh.gov.my (Tanya Doktor) for advice from Family Medicine Specialists. For individuals with a history of travelling overseas, please do not go directly to a government healthcare facility. They are advised to firstly seek medical advice through the Virtual Health Advisory or the National CPRC hotline. visit the DoctorOnCall and BookDoc websites<br>Collaboration (national)<br>MOH is collaborating with the private sector to implement COVID-19 sample collection services at homes of individuals who wish to be tested. There will be charges for this service and the details of implementation will be officially informed in the near future.<br>Collaboration with Virtual Health Advisory (DoctorOnCall) and BookDoc. Thos with history of travelling oversease were advised to seek medical advice through Virtual Health Advisory or National CPRC online prior to visit to government healthcare facility
Ministry of Health Malaysia[70]	05/03/2020	Health Advisory - To avoid unnecessary panic and anxiety at workplaces when a confirmed or suspected case is identified amongst the employees, MOH advises organisations that it is unnecessary to close work premises. It is adequate to conduct more intensive cleaning and disinfection at common areas, particularly at frequently touched surfaces. Individuals identified as close contacts by MOH will undergo health screening. MOH advises individuals identified as close contacts to remain calm and to cooperate fully in providing accurate information when contacted<br>Malaysians abroad are also encouraged to register at the nearest Malaysian embassies. Information on Malaysian Embassies and High Commissions are available at the website of the Ministry of Foreign Affairs at kln.gov.my.<br>Outcome of the Technical Working Committee for COVID-19 Cluster Meeting No.2/2020<br>Taking into account the current COVID-19 situation in Malaysia that is currently in the second wave, the Committee agreed on the following measures:<br>Travel advisory:Lombardy, Veneto and Emilia-Romagna, Italy;Hokkaido, Japan; and Tehran, Qom and Gilan, Iran<br>Travel restriction of all visitors (except citizens, permanent residents, social pass holders and Malaysian student passes) who were present or visited the above town or territory within 14 days prior to arrival to Malaysia.<br>To gazette facemasks as a controlled item under the Control of Supplies Regulations (Prohibition of Export) 2020 to

		ensure adequate supply of facemasks for all Malaysians.<br>Coordination<br>Coordination by Ministry of Foreign Affairs: Malaysian Embassies to register Malaysians abroad<br>Regulation / Law<br>Face masks gazetted as a controlled item under the Control of Supplies Regulations (Prohibition of Export) 2020
Ministry of Health Malaysia[71]	06/03/2020	Tracing close contact of confirmed covid-19 by District Health Officer's team for further investigation and testing<br>MOH were monitored current situation continuously<br>Infoline CPRC were made available for further enquiries and advices<br>Detection for sporadic and local cases - surveillance among patients with ILI/SARI with no history of travelling to affected countries or contact with confirmed covid-19 cases.<br>Health advisory (organising public programs/activities)<br>Advice for organisers of public programs and activities<br>- encouraged to conduct an assessment then determine the suitability of continuing as planned<br>- implement appropriate preventive and precautionary measures to reduce the risk of COVID-19 infection amongst the participants - conduct screening for symptoms, adequate hand sanitiser<br>Code of Professional Conduct under the Medical Act 1971 (Amendments 2012) and the Medical Regulations<br>- all medical practitioners must strictly ensure confidentiality of patients' personal and medical information, unless voluntarily revealed by the patients themselves
Ministry of Health Malaysia[72]	07/03/2020	Tracing close contact of confirmed covid-19 by District Health Officer's team for further investigation and testing<br>MOH were monitored current situation continuously<br>Infoline CPRC were made available for further enquiries and advices<br>Detection for sporadic and local cases - surveillance among patients with ILI/SARI with no history of travelling to affected countries or contact with confirmed covid-19 cases<br>Based on MOH's current assessment, the COVID-19 situation in Malaysia is still under control and in the early containment phase.
Ministry of Health Malaysia[73]	08/03/2020	Detection for sporadic and local cases - surveillance among patients with ILI/SARI with no history of travelling to affected countries or contact with confirmed covid-19 cases<br>MOH monitored current situation continuously
Ministry of Health Malaysia[74]	09/03/2020	Detection for sporadic and local cases - surveillance among patients with ILI/SARI with no history of travelling to affected countries or contact with confirmed covid-19 cases<br>MOH is currently collaborating with the private sector for collection of samples for COVID-19 at home through training of private staffs in managing PUI cases, training in collection and managing of samples and quality control of testing. Sample collection services are by trained private staff and tested at private labs.<br>Collaboration with private sector to provide fee-for-service COVID-19 home sampling for asymptomatic individuals.<br>MOH advises the public the importance of self-care, and if symptomatic to avoid from going to crowded public places. These two simple measures have been proven to reduce the risk of infection through respiratory droplets in the community. These measures were also implemented in the People's Republic of China for the COVID-19 infection and were shown to contribute to the reduction of cases.

		MOH Launching of collaboration with private sector in COVID-19 sampling at home using Uberisation in Health approach. For those who do not fulfil PUI criteria but wish to be tested
Prime Minister's Office of Malaysia[68]	10/03/2020	Get medical attention immediately if not feeling well (ie, have respiratory tract infections such as fever, cough or difficulty breathing) within 14 days after returning home. Inform the doctor about history of travel in affected countries.
Ministry of Health Malaysia[75]	11/03/2020	Guidelines<br>Covid-19 Guidelines for Workplaces released, which include advise to consider<br>- alternate communication methods e.g. virtual meetings in place<br>of face to face meetings, group chats etc.<br>- deferring large meetings or events
Ministry of Health Malaysia[76]	11/03/2020	The International Health Regulations (IHR) Focal Point for Brunei Darussalam has notified the Malaysian IHR Focal Point of the confirmed COVID-19 case detected in their country. This case was reported to have a history of travelling to a Tabligh (religious) gathering in Seri Petaling mosque in Selangor from 27 February 2020 until 1 March 2020.Based on preliminary information, an estimated 10,000 individuals from several countries, including Malaysia, participated in this gathering. The participants from Malaysia were estimated to be over 5,000 individuals. All State Health Departments are currently conducting investigations in their respective states.<br>All individuals who attended to Tabligh gathering with symptoms to immediately contact nearest district Health Office or State CPRC or contact Virtual Health Advisory. Those without symptoms to practice social distancing<br>600 samples had been tested. One tested positive. Daily reporting<br>MOH advised the public not to travel to countries affected by the COVID-19 infection<br>MOH monitored current situation continuously<br>Infoline CPRC were made available for further enquiries and advices<br>Health advisory<br>- Asymptomatic attendees advised to practice social distancing by maintaining distance of at least 1 metre from other individuals for 14 days from the last date of attending the gathering.<br>- MOH strongly urges all individuals who attended the gathering to give their full cooperation with MOH to ensure that the COVID-19 infection does not spread further in the community.<br>MOH also strongly advises that:<br>- To postpone all mass gatherings involving the public to reduce the risk of COVID-19 infection;<br>- If unwell with respiratory tract infection symptoms, not to attend events or programs involving the public or in crowded public places, including religious activities;<br>- Individuals with symptoms are advised to practice social distancing, of at least 1 metre distance from other individuals;<br>- The public is advised not to travel to countries affected by the COVID-19 infection; and<br>- Malaysians with respiratory tract infection symptoms not to visit or travel to other parts of Malaysia during the upcoming school break.
Ministry of Health Malaysia[77]	12/03/2020	IPC: Social distancing<br>COVID-19: Social distancing guidelines for workplace, homes and individuals released (includes schools and childcare, assisted or senior living facilities)

Ministry of Health Malaysia[78]	12/03/2020	MOH has also notified the International Health Regulations (IHR) Focal Points of countries whose citizens participated in this gathering<br>The public was urged not to disseminate the unverified information related to the COVID-19 infection involving the tabligh assembly in order to maintain public harmony.
Ministry of Housing and Local Government [79]	12/03/2020	(PBT) to ensure that all supermarkets/ hypermarkets / grocery stores practice IPC
Zainul [80]	12/03/2020	When the pandemic first started, the daily testing capacity was at 3,500.
[81]	13/03/2020	Infection and prevention control<br>- Public discouraged to attend gatherings or go to crowded places including religious places, especially those with respiratory symptoms.<br>- All public mass gatherings to be postponed.
Bernama [82]	13/03/2020	Meanwhile, Health Minister Datuk Seri Dr Adham Baba, at a ceremony at the Higher Education Leadership Academy (Akept) in Nilai, said individuals without fixed income or salary would be given aid of RM100 a day during the quarantine period. The fund, which is managed by the National Disaster Management Agency (Nadma), covers those placed under 14-day home surveillance as well as Covid-19 patients being treated at hospitals.
Kaos[83]	13/03/2020	Foreign Minister Datuk Seri Hishammuddin Hussein said currently 11 Malaysians abroad had been identified as Covid-19 positive, but there were concerns that there might be more among those who had not registered with Wisma Putra."Considering there is a large number of Malaysians abroad, I am urging those who have not registered themselves with our embassies to do so immediately. "Registering is important to ensure that our representatives can provide consular assistance to those who need them."At the same time, I want to advise Malaysians to always take preventive measures as recommended by the Health Ministry."Obey the instructions of the local authorities at all times. If you have Covid-19 symptoms, immediately seek treatment at the nearest health facility.
Ministry of Health Malaysia [84]	14/03/2020	The MOH would like to affirm the Government's decision announced through the Prime Minister's Message of the Prime Minister of Malaysia that all assemblies including international meetings, sports, social and religious events be postponed or cancelled as of April 30, 2020. This decision will be reviewed as the COVID-19 situation improves.<br>To reduce the risk of COVID-19 infection in vulnerable communities and vulnerable populations especially in remote areas such as the Orang Asli community, the MOH recommends that any organization or group that wishes to carry out activities with these communities to postpone meeting with the community.<br>MOH welcomes all forms of medical or health support, contribution of medications and medical collaboration from any organisation, including Non-Governmental Organisation (NGO). MOH requests that any offers or proposals be submitted to the MOH via email cprc@moh.gov.my for further discussion.<br>Screening activity for Tabligh members at Seri Petaling mosque and the mosque was used as a facility for 14-day home surveillance<br>Health advisory

		Risk reduction in hospital:<br>- Visitors to hospitals will be limited to two (2) individuals per patient at any one time;<br>- Children under the age of 12 years are not allowed as visitors to the wards;<br>- Individuals with influenza-like symptoms eg. sore throat, cough, runny nose, fever, vomiting and diarrhoea are not allowed as visitors to the wards; and<br>- Individuals with high risk of infection, particularly those with chronic diseases or with low immunity (e.g. cancer patients on treatment) are not allowed as visitors to the wards, except under extenuating circumstances.<br>All State Health Department were conducting contact tracing in their respective states to identify individuals participated in the Tabligh gathering.
Hammim [85]	14/03/2020	The Health Ministry will deploy 1,000 housemen to hospitals nationwide in an effort to ease the burden of doctors who are busy dealing with the Covid-19 outbreak.
Ministry of Health Malaysia, [86]	15/03/2020	Addressing public concern<br>- MOH notifies the public that all State Health Departments and District Health Offices are conducting activities to control COVID-19 cases among attendees of the tabligh event as well as other cases.<br>- Public are advised to refrain from sharing or disseminating unverified news or information.
Ministry of Health [87]	15/03/2020	MOH urges all individuals attended to Tabligh gathering to contact the nearest District Health Office for further management<br>All state health department and district health office were observing covid-19 activities amongst tabligh members and other suspected individuals
[88-97]	13/0[98]3/2020	1309 buildings nationwide were officially gazetted (through 11 published Federal Gazettes since 16 March) as quarantine stations
[99]	16/03/2020	Collaboration (national)<br>Collaborate with Non-Governmental Organisations (NGOs) and private healthcare facilities in managing COVID-19 case<br>Local event<br>DORMM (Disease Outbreak Response Matrix Malaysia) Late containment phase. MOH communicates preparation to enter into mitigation phase:<br>- Designate Sungai Buloh Hospital as the dedicated admitting hospital for confirmed COVID-19 cases;<br>- To strengthen the capacity and capabilities of laboratories for COVID-19 testing;<br>- Identify quarantine centres throughout the country; and<br>- Collaborate with Non-Governmental Organisations (NGOs) and private healthcare facilities in managing COVID-19 case<br>Postpone travel overseas particularly to regions and cities that have been declared with COVID-19 infection in the People's Republic of China (as announced earlier), the Republic of Korea, Japan, Italy and Iran.<br>The strategy taken to mitigate the spread of COVID –19 virus was strengthening the capacity and capabilities of national laboratories involved. A laboratory testing protocol for both PUI and asymptomatic contact was published following WHO guidelines<br>Health advisory<br>Health advisory extended to the public:<br>- Wash hands frequently

		- Practice good cough etiquette<br>- Postpone organising mass gatherings<br>- Avoid mass gatherings<br>- Practice social distancing of 1 metre<br>- Postpone travel overseas, particularly to affected areas
Prime Minister’s Office of Malaysia [100]	16/03/2020	Local event<br>Announcement of MCO (18 March 2020 - 31 March 2020)<br>Malaysia PM announces Movement Control Order will be enforced from 18 March 2020 until 31 March 2020.Complete restriction of movement and assembly nationwide, A complete travel restriction for all Malaysians going overseas. A complete restriction of foreign visitors and tourists into Malaysia. Closure of all kindergartens, public and private schools, Closure of all public and private institutions of higher learning nationwide, including skills training institutes. Closure of all government and private premises except those involved in essential services<br>"I would also like to announce that a special session of the National Security Council will be convened daily to monitor the COVID-19 situation. I will be chairing this session and will be updating all of you, my fellow Malaysians, from time to time.<br>Should any of you have any questions regarding this Restriction of Movement Order, you may contact the National Operation Management Centre at their hotline 03-8888 2010 beginning 12 noon tomorrow."
Aziz[101]	16/03/2020	The Covid-19 outbreak has now come under the purview of the National Security Council (MKN), said Prime Minister Tan Sri Muhyiddin Yassin.<br>At a press conference today, Muhyiddin said all ministries and relevant agencies will have a meeting this evening, to be followed by live broadcast by him at 9pm.<br>“We will discuss what are the measures and steps that need to be taken, and how we manage this crisis.
Attorney General's Chambers of Malaysia [102]	17/03/2020	All states and federal territories in Malaysia were declared as infection local areas under Prevention and Control of Infectious Disease Order 2020, Prevention and Control of Infectious Disease Act 1988
Attorney General's Chambers of Malaysia [103]	17/03/2020	Under Prevention and Control of Infectious Disease (Measures Within the Infected Local Areas) Regulations 2020 - A citizen or permanent resident of Malaysia returning from overseas shall undergo health examination upon arrival in Malaysia before proceeding for immigration clearance at any point of entry and shall comply with any order issued by an authorised officer.<br>Under Prevention and Control of Infectious Disease (Measures Within the Infected Local Areas) Regulations 2020 - Where an authorised officer requests for any information relating to prevention and control of infectious disease from any person, the person shall comply with the request.
Ministry of Health Malaysia[104]	17/03/2020	Those who were unwell were encouraged to get in touch with virtual health advisory or CPRC Infoline. Infoline CPRC were made available for further enquiries and advices.

National Security Council Malaysia [105]	17/03/2020	Country-level operational plan<br>MKN releases FAQs for MCO
Ministry of Health Malaysia[106]	18/03/2020	On16March 2020, the Prime Minister announced the enforcement of the Movement Control Order (MCO) under the provisions of the Prevention and Control of Infectious Diseases Act 1988 [Act 342]and the Police Act 1967 [Act 344], which comes into force on18March 2020.At that point in time, the total number of confirmed COVID-19 cases had reached 790 cases with the first two (2) deaths reported on17March 2020
National Registration Department of Malaysia [107]	18/03/2020	Infection and prevention control the prevention measures taken during MCO
Ministry of Health Malaysia[108]	18/03/2020	Support for healthcare workers (remuneration)<br>Extra allowance for healthcare staffs who involve with covid-19<br>- rm400 for those who involve at least 15 days in a month<br>- rm200 for those who involve less than 15 days in a month
National Disaster Management Agency[109]	18/03/2020	Guideline for Bantuan Khas COVID-19 for those required by regulations to be quarantined and those warded for COVID-19 who lost their income as a result.
National Security Council Malaysia [110]	18/03/2020	The public is aware that the Government has declared the Movement Control Order from March 18 to 31, 2020. 2. In addition to the declaration, follow-up measures have been taken to coordinate and detail the implementation of the Movement Control Order. National Security Council, Prime Minister's Department has a coordination meeting between government agencies on March 17, 2020 to detail the implementation of the Movement Control Order so that the Order can operate smoothly and efficiently To ensure that the public's need for basic services is not affected during the duration of this Movement Control Order, certain services such as the Appendix A (Essential) and in Appendix B (Non-Essential) are permitted to operate.<br>To implement the Movement Control Order, the Regulation of the Prevention and Control of Infectious Diseases (Measures Within the Local Area of Infections) 2020 was published on March 18, 2020. The Royal Malaysian Police and RELA were appointed as authorized officers under Section 3 of the Prevention and Control Act Infectious Diseases (Act 342).
Ministry of Health Malaysia[111]	18/03/2020	MOH wishes to inform that for the duration of the Movement Control Order, no visitors will be allowed in the hospital wards, except for emergency cases or exceptional circumstances. During this same time period, only one individual will be allowed to accompany a patient for clinic appointments, except for children, those requiring additional assistance or emergency cases.<br>Regulation / Law Regulations Following the announcement of the Movement Control Order by the Prime Minister of Malaysia on 16 March 2020, a Federal Government Gazette was issued [P.U.(A) 87, dated 17 March 2020] declaring that all States and Federal Territories in Malaysia are infected local areas. This is in line with the provisions under Section 11 of the Prevention and Control of Infectious Diseases Act 1988 [Act 342]. Prevention and Control of Infectious Diseases

		(Measures Within The Infected Local Areas) Regulation 2020 was also gazetted [P.U.(A) 91, dated 18 March 2020]. This Regulation contains provisions that require attention and action by the public with regards to the Movement Control Order to prevent further spread of the COVID-19 infection<br>IPC in hospitals - No visitors will be allowed in the hospital wards, and one individual allowed to accompany patients for clinic appointments<br>If unwell, please contact the Virtual Health Advisory or the National CPRC MOH hotline (8 am to 5 pm). The public can also obtain updated information on the COVID-19 situation in Malaysia through the CPRC MOH Telegram channel https://t.me/cprckkm.a
Ministry of Women, Family and Community Development[112]	18/03/2020	Essential services<br>Talian Kasih, co-ordinated by the Ministry of Women, Family and Community Development continues it's operations.
National Security Council Malaysia [113]	18/03/2020	Essential services<br>Manufacturing industry will continue operations, monitored by the Ministry of International Trade and Industries (MITI)
Ministry of Health Malaysia[114]	20/03/2020	While staying at home, Malaysians are encouraged to stay active, whether through exercise or physical activity.<br>Nowadays, there are various indoor exercises that can be done, for example: Cardio-based exercises for those who have exercise bicycles or treadmills at home. Exercises that use your own body weight without the need for specialised equipment such as push-ups, squats, planks, burpees and lounges, Tabata, and lightweight exercises such as yoga or tai chi. Malaysians can also stay active by doing various forms of dances and taking the opportunity to do spring cleaning in and around the house. Studies have shown that exercise and physical activity are important for maintaining mental health by improving mood and relieving anxiety and stress. It helps to sharpen the mind, improve muscle strength and aerobic strength<br>Those who were unwell were encouraged to get in touch with virtual health advisory or CPRC infoline.
Palansamy[115]	20/03/2020	"In the meeting today, it was decided that the military help the police in monitoring the events happening currently. Especially in ensuring that the people abide by the order to stay put in their homes. "The military will be deployed beginning this Sunday. So, we are confident that with the help from the military, the control order would be enforced better and we hope the people would continue to abide by the government order," Ismail said.
Ministry of Health Malaysia[116]	21/03/2020	To date, a total of 19 MOH healthcare workers (HCWs) and 5 HCWs from private healthcare facilities have been diagnosed with COVID-19. Of the 19 MOH HCWs, two (2) are currently in the ICU. The sources of infection for all these HCWs were not due to non-compliance to guidelines in managing COVID-19 cases in their respective healthcare facilities. Investigations found that they were all the first- and second-generation close contacts to the Tabligh gathering cluster.<br>MOH reminds all HCWs managing patients on the importance of using facemasks, maintaining good personal hygiene, particularly washing hands correctly and more frequently.<br>MOH is working closely with the United Nations High Commission for Refugees (UNHCR) to ensure that refugees and asylum seekers are also covered in the preventive and control activities undertaken by the Government. This is to

		prevent any potential spread of infection in the community. To track down individuals involved with the Tabligh gathering within this group of people, UHNCR has collaborated with IMARET, the Malaysian Relief Agency and MERCY Malaysia as their Non-Governmental Organisation (NGO) partners. MOH is also involved through the involvement of HCWs at the District Health Office level. These joint teams will channel information about the COVID-19 infection to refugees and asylum seekers. This includes the importance of seeking early treatment if unwell with respiratory tract infection symptoms, even if they did not attend the gathering. This takes into account that they live in small communities and have close interaction<br>To track down individuals involved with the Tabligh gathering within this group of people, UHNCR has collaborated with IMARET, the Malaysian Relief Agency and MERCY Malaysia as their Non-Governmental Organisation (NGO) partners. MOH is also involved through the involvement of HCWs at the District Health Office level<br>Those who were unwell were encouraged to get in touch with virtual health advisory or CPRC infoline.<br>Infoline CPRC were made available for further enquiries and advices<br>If unwell, please contact the Virtual Health Advisory for advice, and join the webinar session that is held daily starting at 9:00 am via the following link: http://www.doctoroncall.com.my/coronavirus The public can also contact the National CPRC MOH hotline. Further information on the COVID-19 situation in Malaysia is also available through the National CPRC MOH Telegram channel https://t.me/cprckkm.
Daim[117]	21/03/2020	Its director-general Datuk Dr Noor Hisham Abdullah said currently there were about 925 ventilators being used at 26 general hospitals designated for the Covid-19 treatment.<br>"We need 500 more ventilators to prepare for the rising number of cases," he told a Press conference here today. He said the 26 designated hospitals currently had the capacity of 3,400 beds and 300 in Intensive Care Units (ICU).
Yusof[118]	21/03/2020	KUALA LUMPUR: A total of 212 Malaysians arrived home last night on a flight sponsored by the Uzbekistan government. In a statement, Foreign Ministry Deputy Minister Datuk Kamaruddin Jaafar said all 212 Malaysians, who were stranded in Tashkent, Uzbekistan, departed the country on a Uzbekistan Airways flight on March 21. “The passengers arrived safely at the Kuala Lumpur International Airport at 9pm. “The flight was sponsored fully by the Uzbekistan government and the same aircraft will be used to fly home around 200 Uzbekistan nationals who have been stranded here, at 11.30pm. This effort was coordinated by the Uzbekistan Embassy in Kuala Lumpur.
Ministry of Health Malaysia[119]	21/03/2020	Started daily press conference
Ministry of Health Malaysia[120]	22/03/2020	Screening in surgery - every patient was screened at all possible entry point of the department. 3 questions were asked to all patient
Ministry of Health Malaysia[121]	22/03/2020	Screening for pregnancy - every patient was screened at all possible entry point of the department. 3 questions were asked to all patient
Ministry of Health Malaysia[122]	22/03/2020	All patients and visitors were screened for fever at entrance to HDU - 3 questions were asked

Ministry of Health Malaysia[123]	22/03/2020	Home sampling can be done for asymptomatic, non PUI, no history of contact with confirmed case.
Ministry of Health Malaysia[124]	22/03/2020	All patients attending ORL clinic were going for triage for temperature check upon entry to specialist clinic complex, had overseas visits in the last 14 days, attended itjimak gathering symptomatic, had close contact
Ministry of Health Malaysia[125]	22/03/2020	Notification form to be filled up by HCWs to report the suspected and confirmed cases.
Ministry of Health Malaysia[126]	22/03/2020	Surveillance at POE during MCO
Ministry of Health Malaysia[127]	22/03/2020	Those who had travelled or stayed in affected countries over the past 14 days were requested to declare their health status.
Ministry of Health Malaysia[128]	22/03/2020	Passenger Locater form - traveller need to complete the form and would be contacted if public health officer detect suspected or confirmed covid-19 onboard a flight.
Ministry of Health Malaysia[129]	22/03/2020	Opening Times of Primary Healthcare Facilities During the period of Movement Control Order - MOH emphasises that despite the Movement Control Order, MOH will continue to deliver healthcare services to all Malaysians. Therefore, starting from 22 March 2020 (Sunday), the opening times of MOH primary healthcare facilities are as follows:<br>Klinik Desa (Rural Health Clinics) - Attendances less than 20 patients per day - 8.00 am – 1.00 pm<br>Community Clinics: 9.00 am – 6.00 pm<br>UTC Health Clinics: Closed<br>Slight change on the opening times of primary healthcare facilities for continuation to deliver healthcare services to public during the period of MCO.
Ministry of Health Malaysia[130]	22/03/2020	non-Hospital Quarantine Centre - At the national level, the National Security Council is in-charge, the State Secretary at the state level and the District Officer at the district level in overall management of the centre. However, it involves the cooperation of multiple agencies which include District Health Office, District Welfare Department, Malaysian Royal Police, Army, Local Council and RELA, PGA etc.
Bernama[131]	22/03/2020	The government is bringing home 46 Malaysians stranded in Iran on a special flight that is scheduled to touch down at Kuala Lumpur International Airport 2 (KLIA2) at 6.10am Sunday.
Ministry of Health Malaysia[132]	23/03/2020	Secured 33 million PPE and 3 million from donation<br><br>MOH advises the public to remain at home for the duration of enforcement of the Movement Control Order. The public must continue to practice a healthy lifestyle and always maintain good personal hygiene.<br>The public is also encouraged to clean the areas in and around their homes and premises, including disinfection.

		If unwell, please contact the Virtual Health Advisory for advice, and join the webinar session that is held daily starting at 9:00 am via the following link: http://www.doctoroncall.com.my/coronavirus The public can also contact the National CPRC MOH hotline. Further information on the COVID-19 situation in Malaysia is also available through the National CPRC MOH Telegram channel https://t.me/cprckkm .
Povera[133]	23/03/2020	Members of the Malaysian Armed Forces (MAF) and policemen operating in three shifts had been deployed to several parts of the Federal Territory to ensure compliance of the two-week long MCO that is expected to last until end of this month. Additionally, enforcement officers from the Kuala Lumpur City Council (DBKL), doctors and nurses are monitoring the situation.
Povera and Yunus[134]	23/03/2020	On boosting the funding for the country's healthcare facilities and medical front liners in the fight against Covid-19, Muhyiddin said the additional RM500 million allocation would be used for procurement of additional equipment such as ventilators, personal protective equipment (PPE) as well as equipment and facilities needed for intensive care unit (ICU) and Covid-19 screening laboratories.<br>"The government has approved emergency procurement procedures to hasten the procurement of these critical items," he said.<br>The government, he said, has also set aside RM100 million for the ministry to make 2,000 new appointments especially nurses on contractual basis in the fight against Covid-19.<br>"The government is aware that there are many nurses and hospital staff who are tired from working day and night performing Covid-19 infection screening and providing treatment to those tested positive for the infection.<br>"I hope the new appointments could be made immediately to ensure hospitals in the country have sufficient number of workers to prevent the spread of Covid-19," he said.
Ministry of Health Malaysia[135]	24/03/2020	Guidelines (clinical)<br>Guideline COVID-19 Management No.5/2020 update on 24 March 2020<br>Annex 1 : Case Definition - 22 MAC 2020<br>Annex 2 : Management of PUI and Confirmed Case<br>Annex 2a : Management of PUI Not Admitted - 22 MAC 2020<br>Annex 2b : Management of PUI Admitted - 22 MAC 2020<br>Annex 2c : Screening And Triaging - 22 MAC 2020<br>Annex 2d : Work Process Of Pre Hospital Care And Emergency And Trauma Department - 22 MAC 2020 Annex 2e : Clinical Management Of Confirmed Case<br>Annex 2f : Flow Chart For Home Sampling Of COVID-19 - 22 MAC 2020<br>Annex 2g: Surveillance for COVID-19<br>Annex 3 : List of COVID Hospitals and Screening Centres<br>Annex 4b : Senarai Pegawai Untuk Dihubungi Untuk Penghantaran Sampel Di Luar Waktu Pejabat, Hujung Minggu Dan Cuti Umum<br>Annex 5a : Guidelines on Laboratory Testing For Novel Coronavirus For Patients Under Inevestigation<br>Annex 5b : Laboratory Testing For Inpatient<br>Annex 5c: Triple Layer Packaging (For Sample Transportation)<br>Annex 6a: Health Alert Card

		Annex 6b: Mental Health Alert Card<br>Annex 7: Notification Form<br>Annex 8 : The Infection Prevention And Control (IPC) Measures<br>Annex 9 : Management Of COVID-19 At Point Of Entry<br>Appendix 1 : Health Declaration Form COVID-19<br>Appendix 2 : Passenger Locator Form (as per WHO/ICAO)<br>Appendix 3 : Report Of Measures Taken On Board The Flight<br>Appendix 4 : Flow Chart For Screening Of Travellers And Crews Arriving From China At International Point Of Entry<br>Annex 10a : Home Assessment Tool dalam Bahasa Malaysia<br>Annex 10b : Home Assessment Tool dalam Bahasa Inggeris<br>Annex 11: Protocol For Ambulance Transfer For Patient Under Investigation (PUI) COVID-19<br>Annex 12 : Management Of Close Contacts of Confirmed Case<br>Annex 13 : Field Response Activity<br>Annex 14a : Order For Supervision And Observation At Home<br>Annex 14b : Perintah Pengawasan & Pemerhatian Di Rumah Kediaman<br>Annex 15 : Borang Pemantauan Harian Bagi Kontak Rapat Kepada Kes Yang Berpotensi Dijangkiti COVID-19<br>Annex 16 : Borang Senarai Kontak rapat Kepada Kes Confirmed COVID-19<br>Annex 17 : Pelepasan Dari Menjalani Perintah Pengawasan Dan Pemerhatian Di Rumah Kediaman<br>Annex 18 : Borang Pemantauan Person Under Surveillance<br>Annex 19 : Borang Senarai Person Under Surveillance<br>Annex 20 : Interim Guideline For Handling Dead Bodies Of Suspected / Probable /Confirmed COVID-19 Death<br>Annex 21 : Management of Healthcare Workers During COVID-19 Outbreak<br>Annex 22 : Guidelines On Management Of Coronavirus Disease 2019 (COVID-19) In Surgery<br>Annex 22 : Guidelines On Management Of Coronavirus Disease 2019 (COVID-19) In Surgery<br>Annex 24 : Workflow And Work Process For Radiological Examination During COVID-19 Outbreak<br>Annex 27 : COVID guide for special settings<br>Annex 28 : Dialysis and Nephrology Units<br>Annex 29 : Intensive Care Preparedness and Management For COVID-19<br>Annex 30 : COVID 19 ORL Services<br>Annex 31 : Management COVID 19 in Neonates<br>Annex 32 : Quarantine centre<br>Annex 33 : Mental health and Psychosocial support
Ministry of Health Malaysia[136]	25/03/2020	MOH published COVID-19 : Management Guidelines for Special Settings<br>Every person entering a correctional facility should be screened for symptoms such as fever and cough, recent travel and exposure to COVID-19 positive patients. Symptom screening should be carried out every day for inmates<br>Every person going offshore should be screened for symptoms at embarkation points. Employees should be screened daily while offshore for symptoms and those displaying symptoms should be given mask, isolated and medical advice sought

Ministry of Health Malaysia[137]	24/03/2020	MOH is also collaborating with MERCY Malaysia in providing psychosocial support services to all Malaysians. Therefore, all front-liners and individuals emotionally affected by COVID-19 can access the service through these following telephone numbers:<br>011-63996482 / 011-63994236 / 03-29359935<br>The operating hours for this psychosocial support services are from 8.00 am to 5.00 pm daily. This service is managed by MOH's psychology team and MERCY Malaysia volunteers.
Reuters[138]	24/03/2020	7000 tests carried out on a daily basis
Bernama[139]	24/03/2020	The Ministry of Health has identified some 3,000 contract nurses and medical officers to help it deal with the Covid-19 outbreak.<br>Health director-general Datuk Dr Noor Hisham Abdullah said the ministry had the names of those to be called to serve. "... we are in the process of hiring and they will be placed in the areas according to their expertise," he told a daily media conference on the development of Covid-19, here today.<br>Yesterday, Prime Minister Tan Sri Muhyiddin Yassin announced that the government had agreed to set aside an additional allocation of RM600 million to the MOH to combat Covid-19. Of the total, RM500 million is to purchase medical equipment, such as ventilators, essential intensive care unit (ICU) equipment, extra personal protection equipment (PPE) for general medical staff and laboratory equipment for the screening of Covid-19. The remaining RM100 million is for the MOH to appoint 2,000 new staff on a contract basis, especially nurses. He said the ministry was planning to convert training centres or hostels into temporary wards to treat affected patients. The move would provide an additional of 3,000 beds for Covid-19 cases, he said. Dr Noor Hisham added that the ministry would also mobilise health personnel from other states to hospitals designated for Covid-19 cases. "We can't build a hospital in 10 days, (thus) by doing so, we hope we can provide the best treatment to Covid-19 patients," he said.
Attorney General's Chambers of Malaysia[140]	25/03/2020	PREVENTION AND CONTROL OF INFECTIOUS DISEASES (DECLARATION OF INFECTED LOCAL AREAS) (EXTENSION OF OPERATION) ORDER 2020ACT 342 - Prevention and Control of Infectious Diseases Act 1988
Ministry of Health Malaysia	25/03/2020	Infection and prevention control<br>HCWs and front liners advised to constantly practice preventive and precautionary measures as recommended, in particular always maintain hand cleanliness, practice social distancing and use personal protective equipment (PPE) as required.<br><br>All HCWs and front liners who attended to or had close contact to confirmed case from any related cluster events had to informed their respective head of department<br><br>For medical or health advice, the public can contact the Virtual Health Advisory from 8.30 am to 5.00 pm daily, and also join the MOH Facebook Live session from 10.00 am to 10.30 am and DoctorOnCall Facebook Live sessions from 3.00 pm to 3.30 pm, Monday to Friday.<br>The public can also contact the National CPRC MOH hotline. Further information on the COVID-19 situation in Malaysia

		is also available through the National CPRC MOH Telegram channel:<br>https://t.me/cprckkm.
	25/03/2020	1. Loans/financing to individuals and SMEs<br>To ease the cash flow of individuals and SMEs that are likely to be the most affected by Covid-19, banking institutions will offer a deferment of all loan/financing repayments for a period of 6 months, with effect from 1 April 2020. This offer is applicable to performing loans, denominated in Malaysian Ringgit, that have not been in arrears for more than 90 days as at 1 April 2020. For credit card facilities, banking institutions will offer to convert the outstanding balances into a 3-year term loan with reduced interest rates to help borrowers better manage their debt.<br>2. Loans/financing to corporations<br>Banking institutions will also facilitate requests by corporations to defer or restructure their loans/financing repayments in a way that will enable viable corporations to preserve jobs and swiftly resume economic activities when conditions improve. Corporations should approach their banking institutions to discuss their repayment plans and the restructuring of credit facilities.
	25/03/2020	Dr Noor Hisham said the MOH also identified 3,000 contract nurses and medical staff to assist. MoH is targeting an additional 5,000 beds with 800 ventilators, with 500 of them specifically for Covid-19 patients. At present, the country has only 219 respiratory aids. "We will receive 40 units by Friday and another 200 by next week," he said, adding that 60 respiratory aids were lent by the private sector.
	26/03/2020	PREVENTION AND CONTROL OF INFECTIOUS DISEASES (COMPOUNDING OF OFFENCES) (AMENDMENT) REGULATIONS 2020<br>ACT 342 - Prevention and Control of Infectious Diseases Act 1988
Ministry of Health Malaysia[141]	26/03/2020	For medical or health advice, the public can contact the Virtual Health Advisory from 8.30 am to 5.00 pm daily, and also join the MOH Facebook live sessions from 10.00 am to 10.30 am and DoctorOnCall Facebook live sessions from 3.00 pm to 3.30 pm, Monday to Friday. The public can also contact the National CPRC MOH hotline. Further information on the COVID-19 situation in Malaysia is also available through the National CPRC MOH Telegram channel https://t.me/cprckkm. MOH will continue to monitor the development of the COVID-19 situation based on all available information, and the public will be continuously updated on the latest information through MOH's website http://www.moh.gov.my/index.php<br>MOH requested all HCWs and supporting staffs from Hospital Teluk Intan to be tested as the outbreak occurred.<br>Healthcare Workers and Risk of COVID-19 Infection. As of 26 March 2020, 12 pm, a total of 80 MOH health care workers (HCWs) have been reported to be positive for COVID-19. Currently, NONE of these cases were from managing confirmed COVID-19 cases in MOH healthcare facilities.<br>Of these 80 cases, 39 HCWs are from the Teluk Intan Hospital, Perak cluster. Based on this current situation, MOH has ordered that all HCWs and support staff of Teluk Intan Hospital to be tested for COVID-19.<br>MOH advises all HCWs and front liners to constantly practice preventive and precautionary measures as recommended, in particular always maintain hand cleanliness, practice social distancing and use personal protective equipment (PPE) as required.<br>Medical practitioners at private hospitals and clinics are also front liners in delivery of healthcare to the public. MOH advises that all private medical practitioners to also constantly practice preventive and precautionary measures as

		recommended above, in particular on the use of face masks during consultations and to use appropriate PPE for any clinical procedures.<br>Maternal and Child Health Services in Health Clinics, Maternal and Child Health Clinics and Klinik Desa (Rural Health Clinics) -<br>Even in the current COVID-19 situation, MOH emphasises that Maternal and Child Health (MCH) Services are functioning as usual.<br>HCWs will still conduct post-natal home visits to ensure that mothers and infants are in good health. For pregnant women or parents currently away from home, they can obtain MCH services from the nearest health clinic (government or private clinic).<br>MOH urges that pregnant women to comply to their appointment dates for antenatal check-ups. Immunisation services for infants and children will also continue as usual. Parents are requested to adhere to the immunisation schedule and appointment dates. MOH emphasises the importance of adhering to the immunisation schedule for children to prevent the occurrence of vaccine-preventable diseases.
Sarawak Multimedia Authority[142]	26/03/2020	Sarawak Multimedia Authority (SMA) today announces the roll-out of the digital surveillance solution to curb the spread of Covid-19 in Sarawak. The solution will give the State Disaster Management Committee the scalable capability to monitor this form of infectious disease at all Sarawak's Points of Entry (POE) On top of the mandatory health declaration, approved persons entering Sarawak will be issued a QR-coded wristband based on the categories they fall under. These categories are known as Person Under Investigation (PUI) and 14-day Stay Home Notice (SHN). There are two within the PUI categories; Home Quarantine and Hospital Quarantine. On a twice-daily basis, wearers are required to report their situation by scanning their wristband's QR code to submit a set of information. Data collated will allow the State Disaster Management Committee to make informed decisions as well as to conduct random checks on the wearers. The wearers' location will enable the Disaster Management Committee to establish hotspots, a key strategy to isolate further spread of the disease.
Ministry of Health Malaysia [143]	27/03/2020	Implemented EMCO due to 74 cases are from Kampung Dato' Ibrahim Majid and Bandar Baharu Dato' Ibrahim Majid in Simpang Renggam, Kluang, Johor.<br>MoH aims to have 300 test samples taken daily starting today. Sampling will be prioritised to close contact groups, symptomatic individuals, and at-risk groups or groups.<br>For information, cleaning and disinfection in public places will be carried out in collaboration with the Ministry of Housing and Local Government and Simpang Renggam District Council. The cleaning work will focus on public areas. Information will also be given to all residents regarding proper home disinfection procedures.<br>Health services at the Dato 'Ibrahim Majid Rural Clinic continue as usual while emergency services will be coordinated by the Medical Emergency Coordination Centre (MECC).<br>MOH closely monitored all positive cases<br>MOH advised the public to constantly practice the various health advisories issued by MOH. Each and every Malaysian can play an important role in reducing the spread of COVID-19 in the country. All Malaysians must: Remain at home Practise social distancing Wash hands frequently with water and soap<br>For medical or health advice, the public can contact the Virtual Health Advisory from 8.30 am to 5.00 pm daily, and also join the MOH Facebook live sessions from 10.00 am to 10.30 am and DoctorOnCall Facebook live sessions from 3.00 pm

		to 3.30 pm, Monday to Friday. The public can also contact the National CPRC MOH hotline. Further information on the COVID-19 situation in Malaysia is also available through the National CPRC MOH Telegram channel https://t.me/cprckkm.
Prime Minister's Office of Malaysia [144]	27/03/2020	A budget of RM500 million was allocated to MOH to implement immediate action in order to curb the spread of COVID-19 virus. Additional RM1 billion was allocated to purchase equipment and services which includes obtaining medical expertise from private healthcare services. To support MOH's effort towards conducting more COVID-19 tests, insurance and takaful industry created a special fund of RM8 million to cover the costs incurred for screening that takes up to RM300 per diagnostic test at private hospitals or laboratories for policy holder and takaful medical certificate. As a recognition to the sacrifices of front liners, Government decided to increase the special allowance to RM600 effectively 1st of April 2020.<br>In the previous package, the Government provided special allowances to doctors, nurses and medical staffs involved directly in curbing and preventing the outbreak. I am aware that in this difficult situation, our healthcare staff are working tirelessly under stress and enormous pressure. Nevertheless, they continue to battle against this great challenge.<br>Recognising their sacrifices, the Government will increase the special allowance from RM400 to RM600 per month effective 1 April 2020 until the outbreak ends. At the same time, the Government also agrees to extend a special allowance of RM200 a month to military, police, customs, civil defence and RELA members who are directly involved in enforcing the MCO. This allowance will also be paid beginning 1 April 2020 until the COVID-19 outbreak ends. About 169,000 additional front liners are expected to benefit from this initiative.
Ministry of Health Malaysia [145]	27/03/2020	The government has also repatriated 162 Malaysians from Ho Chi Minh, Vietnam on Thursday (March 26).
	28/03/2020	Returning Malaysians must undergo Voluntary Quarantine<br>MOH provides channel for donation (bank account and contact for Under-Secretary, Procurement and Privatisation Division, MOH<br>MOH releases bank and contact details for public contributions and donations.
Bernama[146]	28/03/2020	A consignment of medical equipment donated by China, comprising test kits, face masks and protective equipment, has arrived in Malaysia. The consignment consisted of 100,000 test kits, 100,000 N95 medical face masks, 500,000 surgical masks, 50,000 sets of personal protective equipment and 200 ventilators. The consignment was handed over by Chinese Ambassador to Malaysia, Bai Tian to Foreign Minister Hishammuddin Hussein, at Wisma Putra here today. Hishammuddin said Malaysia would make full use of the equipment in the battle to contain the Covid-19 pandemic. "They will be immediately handed over to the National Disaster Management Agency so that our frontliners will be prioritised in their distribution.
Ministry of Health Malaysia[147]	29/03/2020	There are 43 functioning laboratories nationwide which consist a network of 18 MOH laboratories, 12 MOH hospital laboratories, 4 state Public Health Laboratories and 7 private laboratories namely Lablink (M) Sdn.Bhd., Pantai Premier Pathology and Neogenix Laboratories Sdn.Bhd. Collaboration with Ministry of Higher Education (MOHE) and Ministry of Science, Technology and Innovation (MOSTI) resulted in 10 additional diagnostic laboratories at institutions of higher learning including a mobile laboratory that can be deployed at hotspot areas to maximize the number of patients tested

		daily. All ten additional labs were able to carry out 1414 tests on a daily basis.<br>MOH calls out for volunteers to join the health and medical support teams managing COVID-19. Those interested fill out the Registration Form for Volunteers of the COVID-19 Health and Medical Support Team (Borang Pendaftaran Anggota Sukarelawan Pasukan Bantuan Kesihatan Dan Perubatan COVID-19) on the MOH's Facebook page<br>Health advisory<br>For high risk groups (elderly, those with chronic conditions)<br>- ensure adequate and undisrupted supply of medicines<br>- take extra precautions to prevent infection<br>currently there are 157 volunteers and there will be 1008 volunteers from various fields joining<br>Health Advisory on COVID-19 Of the 34 COVID-19-related deaths to date, 55.6% were aged 60 years and above, while 67.6% had a history of chronic diseases such as diabetes, hypertension, kidney disease and heart disease. These individuals are at greater risk of serious COVID-19 infection, with more severe symptoms and complications. Therefore, it is important for them and their family members to take extra precautions to prevent COVID-19 infection. Please ensure that there are adequate and undisrupted supply of medicines for the patients. Healthcare services are running as normal at all MOH healthcare facilities, especially for follow-up appointments and supply of medicines.
Prime Minister's Office of Malaysia[148]	29/03/2020	Second EMCO - Mile 21 to 24 at Sungai Lui, Hulu Langat, Selangor from 30 March 2020 to 13 April 2020.<br>MOH announced Second Enhanced Movement Control Order (EMCO) covering Mile 21 to 24 at Sungai Lui, Hulu Langat, Selangor from 30 March 2020 to 13 April 2020.<br>This is due to the high number of COVID-19 cases at that area: 71 positive cases from 274 students at Maahad Tahfiz AnNabawiyyah at Mile 23, Sungai Lui in Hulu Langat, Selangor. The purpose of EMCO was to contain the COVID-19 infection in the area.
Ministry of Health Malaysia[149]	30/03/2020	Preparedness of Hospitals in Managing COVID-19 Patients MOH has undertaken various preparedness measures for hospital services in managing the COVID-19 situation. Since February 2020, prior to the enforcement of the Movement Control Order (MCO) by the Government on 18 March 2020, MOH has already identified 57 screening hospitals and 26 hospitals for managing COVID-19 patients. At that time, the number of confirmed COVID-19 cases were only 790 cases compared to 2,626 reported today. Presently, through various efforts, 70 hospitals are able to provide COVID-19 screening throughout the country compared to 57 hospitals previously. The number of hospitals for managing COVID-19 cases have also increased from 26 to 38 hospitals in collaboration with the Malaysian Armed Forces and the University of Malaya Medical Centre. Of these, seven hospitals have been assigned as COVID-19 hospitals. As a result, the number of beds for COVID-19 patients have increased to 3,994 beds. MOH is planning to increase the number of beds through the identification and renovation of MOH's Training Institutions (ILKKM) to be designated quarantine and treatment centres with a total of 1,937 beds. The capacity of ILKKM can be further increased if necessary. Other facilities are also being identified, such as the Malaysia Agro Exposition Park Serdang (MAEPS), capable of providing spaces for 600 beds.<br>Individuals who have been ordered to undergo "home or self-surveillance" are required to fully comply to the Observation and Surveillance Order at the pre-determined premises, under Prevention and Control of Infectious Diseases Act 1988 (Act 342).<br>MOH advises individuals with a history of travelling abroad to isolate themselves from close contact with family members and other household occupants for 14 days, 24 hours per day from the date of arrival back in Malaysia. During

		this time period, these individuals are advised to take preventive measures such as good hygiene practices, frequent hand washing and social distancing (a distance of at least one metre) from others. During this 14-day period, these individuals are also required to self-monitor for symptoms such as fever, cough, colds, sore throat or difficulty breathing. Seek immediate treatment if symptoms develop by calling the nearest health facility and notify the healthcare providers of the self-quarantine status. The list of contact details of healthcare facilities can be obtained from the MOH's website.<br>70 hospitals offered screening activities at the moment, previously were 57 hospitals
Prime Minister's Office of Malaysia[150]	30/03/2020	"We will treat COVID-19 positive patients who are asymptomatic or have mild symptoms here," he said.<br>The centre has the capacity to place up to 600 patients at any one time and it will be coordinated by Nadma with the cooperation of the MOH, the Armed Forces, the Fire and Rescue Department, the Civil Defence Force, the Social Welfare Department and the Public Works Department.<br>Among others, it will provide clinical services, COVID-19 wards, resuscitation areas to stabilise patients during emergencies, as well as ambulance, pharmacy and pathology laboratory services.<br>Facilities for patients include beds, tables and chairs, lockers, television, lounges, WIFI access, computers, surau, closed-circuit television (CCTV), toilets and baths, changing rooms and rest areas.<br>For those on duty, there are nurse stations, treatment/resuscitation room, rest areas, changing rooms and baths, including for the 'donning and doffing' of personal protective equipment (PPE) as well as meeting and seminar rooms."
The Malaysian Insight[151]	30/03/2020	Minister Hishammuddin Hussein thanked the United Arab Emirates (UAE) for their contribution of medical equipment to Malaysia today.
Mohsen[152]	30/03/2020	Yesterday (Monday) alone, we brought home 179 Malaysians stranded in Amritsar, India, while another 144 citizens have just arrived from Myanmar today," he said in a live streaming on Facebook yesterday.
Ministry of Health Malaysia[153]		Coalition launched to accelerate research on the prevention and treatment of COVID-19 in low- and middle-income countries<br>MOH Malaysia joined the coalition.
Attorney General's Chambers of Malaysia[154]	31/03/2020	PREVENTION AND CONTROL OF INFECTIOUS DISEASES (MEASURES WITHIN INFECTED LOCAL AREAS) (NO. 2) (AMENDMENT) REGULATIONS 2020<br>ACT 342 - Prevention and Control of Infectious Diseases Act 1988
Ministry of Health Malaysia[155]	01/04/2020	MOH would like to thank concerned parties for sharing nutritional information relating to the SARS-CoV-2 virus and the COVID-19 infection. Unfortunately, MOH has found that these consisted of claims and testimonies of certain types of food, supplements and health practices alleged able to cure and prevent COVID-19 infection. To date, no scientific study has proven the effectiveness of any kind of food, supplements and traditional therapies against the SARS-CoV-2 virus and the COVID-19 infection. To ensure that our body remains healthy and able to fight the infection optimally, we must take a healthy, balanced and varied diet every day. For verified and up to date information, please visit the MOH website http://moh.gov.my/index.php or the Nutrition Division, MOH website http://nutrition.moh.gov.my/.<br>List of volunteers at MOH Hospitals, Quarantine and Low-Risk COVID-19 Patients Treatment Centres according to category

Bernama[156]	01/04/2020	The Ministry of Health (MOH) has received 5,000 units of flocked swabs and Universal Transport Medium (UTM) for COVID-19 sample collection and testing from the Singapore government. The contribution was handed over to Health Minister, Datuk Seri Dr Adham Baba, by Singapore's High Commissioner to Malaysia, H.E. Vanu Gopala, today.
Hariz Mohd[157]	01/04/2020	Director-General of Health Dr Noor Hisham Abdullah said at present, the public hospital has a total of 219 ventilators and another 68 are borrowed from the private sector.<br>However, Noor Hisham said the number would increase to 487 units after Beijing donated 200 ventilators to the Malaysian government.<br>Noor Hisham added that an additional 956 units were acquired, bringing the total number of existing units to 1,443. "
Ministry of Health Malaysia[155]	01/04/2020	A total of 2,359 healthcare workers, including 65 specialists and 159 medical officers, volunteered to manage COVID-19. Two separate Google forms were distributed to recruit volunteers for COVID-19 health and medical support team in hospitals and other healthcare facilities.<br>2nd phase MCO: Reduction of supermarket, convenience store and petrol station hours. MOH advised: To date, no scientific study has proven the effectiveness of any kind of food, supplements and traditional therapies against the SARS-CoV-2 virus and the COVID-19 infection.<br>MOH would like to thank concerned parties for sharing nutritional information relating to the SARS-CoV-2 virus and the COVID-19 infection. Unfortunately, MOH has found that these consisted of claims and testimonies of certain types of food, supplements and health practices alleged able to cure and prevent COVID-19 infection. To date, no scientific study has proven the effectiveness of any kind of food, supplements and traditional therapies against the SARS-CoV-2 virus and the COVID-19 infection. To ensure that our body remains healthy and able to fight the infection optimally, we must take a healthy, balanced and varied diet every day. For verified and up to date information, please visit the MOH website http://moh.gov.my/index.php or the Nutrition Division, MOH website http://nutrition.moh.gov.my/.
Ministry of Health Malaysia[158]	02/04/2020	MOH is very concerned that the current situation under the Movement Control Order (MCO) may cause people to experience various negative emotions such as anxiety, panic, sadness and depression. MOH would like to take this opportunity to call on the public to maintain their mental health by taking the following steps: Avoid being alone, and share your feelings with someone you trust. Speak positive words to yourself, family members and friends. Keep in touch and interact with loved ones and close friends, using a variety of communication mediums. Pray according to your beliefs. Practice relaxation techniques Be physically active at home Eat healthily. Psychosocial support services are available to the public by calling the following numbers: 011-63996482 / 011-63994236 / 03-29359935 (8.00 am to 5.00 pm daily)
Ministry of Health Malaysia[159]	03/04/2020	HCWs must also take all recommended precautionary measures, especially hand hygiene, social distancing and use of personal protective equipment (PPE), especially when performing clinical procedures. It is very important for all HCW and front liners to take care of your physical and mental health, by:<br>Take time to rest in between work shifts and assignments<br>Make sure you eat healthily<br>Don't keep to yourself; share your feelings and emotions with someone you trust<br>Give each other words of encouragement and support<br>Interact constantly with family members<br>Get help from psychosocial support when needed

		MOH is also very grateful to the family members of HCWs for their constant support.<br>Taking on the commitment and enthusiasm these patients have shown in the process of recovering from the COVID-19 infection, MOH hopes this will give hope to other patients and the public to remain optimistic in facing the COVID-19 situation. The public is also urged to avoid stigma against these individuals and instead provide moral support to those affected.<br>MOH urged the public who come for treatment to provide honest information about any history of close contact with confirmed COVID-19 case(s), attendance at any large gatherings and history of travelling overseas.
Nik Anis[160]	03/04/2020	All Malaysians who return to the country will be immediately quarantined for 14 days, says Senior Minister Datuk Seri Ismail Sabri. This ruling will take effect from April 3, he said. He said those coming back – either on chartered or commercial flights – will immediately sent to government quarantine centres as soon as they step off the plane. “We noted that some countries are experiencing an increase in Covid-19 cases because its citizens are returning from abroad.
Ministry of Health Malaysia[161]	04/04/2020	Surveillance (contact tracing)<br>- MOH continued to work closely with various agencies for contact tracing activities.<br>- Those who had contact with the Seri Petaling cluster was urged to contact the nearest District Health Office for screening.
Bernama[162]	04/04/2020	On April 4, 101 Malaysians, including students in Thailand, took the Malaysia Airlines special flight to return home.
Khairulrijal[163]	04/04/2020	Three days is all the government needed to transform the exhibition halls of the Malaysia Agro Exposition Park (Maeps) into a gigantic temporary makeshift hospital for Covid-19 patients. The hospital, which is the brainchild of Prime Minister Tan Sri Muhyiddin Yassin, can accommodate a maximum of 604 patients. It was set up to ease congestion at gazetted Covid-19 hospitals by accepting low risk patients who only show mild symptoms of the virus. The Health Ministry and other government agencies, such as the police and army, rolled up their sleeves and worked day and night to ensure the project was completed within the stipulated time.
Ministry of Health Malaysia[164]	05/04/2020	Implications of Withholding History of Close Contact or Travelling Overseas MOH emphasises the importance of the public to provide honest and accurate information of any history of close contact with confirmed COVID-19 cases, history of travelling overseas or history of attending any gatherings as announced by MOH to be closely related to COVID-19 infection transmission. For example, investigations have shown that a COVID-19 cluster was due to an index case (Case 1,580) with a history of travelling to Italy. So far, in this cluster there are 37 confirmed COVID-19 cases, of which there were five deaths (Cases 1,031, 1,032, 1,006, 2,850 and 2,210) and one case currently in a critical condition requiring ventilation support in the ICU. There are already three generations of infection in this cluster.<br>In accordance with the Movement Control Order (MCO), Malaysians returning from overseas will be placed at quarantine stations provided. They will undergo a health screening, (temperature and COVID-19 symptoms) at all international ports of entry. COVID-19 testing will be conducted, and they will be monitored for 14 days at the quarantine stations.<br>If any individual is symptomatic upon arrival at the international ports of entry or while at the quarantine stations, they will be sent to designated hospitals for further investigation and treatment. In addition, those found to be positive for COVID-19 for the screening tests will be referred to designated hospitals for isolation, further investigation, treatment

		and monitoring. This is done to prevent further spread of COVID-19 infection in the community and break the transmission chain in the country.
Ministry of Health Malaysia[165]	06/04/2020	Malaysia starts global “Solidarity Trial” – a research effort to test possible treatments for COVID-19
Bernama[166]	06/04/2020	“To lighten the burden of patients, from April 6 to June 30, the cost of medication delivered by post, estimated to be RM638,000, will be borne by the MOH.<br>The patient’s adherence with — and preventing interruption to — the medication regimen is important, including practising social distancing, during the MCO,” he said in his Facebook page today.<br>He said normally the patient would pay Pos Malaysia between RM5.30 and RM10.60 in cash upon delivery depending on weight and zone of destination.
Khairulrijal[167]	06/04/2020	The National Security Council (MKN) is the only government agency which can issue directives during the Movement Control Order (MCO), said Senior Minister (Security Cluster) Datuk Seri Ismail Sabri Yaakob.
Prime Minister's Office of Malaysia[168]	07/04/2020	The COVID-19 Fund was launched on March 11 as a government effort to help people affected by the outbreak.
Ministry of Health Malaysia[169]	08/04/2020	MOH strongly emphasises the importance of all Malaysians in complying to all health advisories that have been issued. Every level of the society must play their respective roles responsibly. Compliance to the MCO and all health advisories is very crucial in preventing widespread infection transmission in the community. MOH has found that unauthorised movements outside homes are still taking place, involving visits to relatives’ homes including visiting family members who are unwell, as well as those undergoing home- or self-surveillance or monitoring.
United Nations High Commissioner for Refugees[170]	08/04/2020	UNHCR-GoM joint action to prevent, manage COVID-19 infections among refugees<br>- UNHCR has been coordinating closely with the MOH and the CPRC to ensure that all refugee and asylum-seeking communities are included in the Government’s national response measures, and to prevent infection from spreading among the communities<br>- Joint teams: UNHCR, district health officers, UNHCR’s NGO partners (IMARET, Muslim Relief Agency, and MERCY Malaysia)<br>Measures<br>- Free testing and treatment for any foreigners with Covid-19 symptoms.<br>- Undocumented foreigners who come forward for testing and treatment would not face arrest for Immigration offences.
Ministry of Health Malaysia[171]	09/04/2020	Deputy Secretary General (Management) Circular - Notification About MOH Fwd My Heroes Fund By Fwd Takaful Berhad<br>Provided extra insurance coverage for MOH staff
Ministry of Health Malaysia[172]	09/04/2020	MOH is working closely with the United Nations High Commission for Refugees (UNHCR) to ensure that refugees and asylum seekers are also included in the prevention and control activities undertaken by the Government.

		NGOs and volunteers assisted in field surveillance activities.<br>Non-compliance to Movement Control Order is punishable under Section 24 of Act 342.
Ministry of Health Malaysia[173]	10/04/2020	National Institute of Forensic Medicine, MOH had conducted an autopsy on a COVID-19 patient. This autopsy was done under Section 16 of the Prevention and Control of Infectious Diseases Act 1988 (Act 342). The autopsy revealed that the COVID-19 virus was detected on the surface of the body through real-time Polymerase Chain Reaction (rt-PCR) analysis conducted by the Institute for Medical Research (IMR). This finding supports the views submitted to MOH previously and approved by the "Mesyuarat Jawatankuasa Muzakarah Khas Majlis Kebangsaan Bagi Hal Ehwal Ugama Islam Malaysia" (Meeting of the National Special Council for Islamic Religious Affairs of Malaysia) that convened on 15 March 2020, as well as the Malaysian Consultative Council for Buddhists, Christians, Hindus, Sikhs, and Taoists Religions Meeting that convened on 16 March 2020. This includes the management of bodies and burial. For those of Islamic faith, only the tayammum (dry ritual purification using purified sand or dust, instead of ritual washing with water) is allowed on the surface of the body bag. Whereas for those of other religions, any religious or traditional rituals can only be conducted on the surface of the body bag.<br>MOH welcomes the further two-week extension of the Movement Control Order (MCO) as announced by the Prime Minister. These will be in line with the statement made by the Director General of the World Health Organization (WHO), Dr Tedros Adhanom Ghebreyesus on 25 March 2020, that called upon aggressive measures, such as the Movement Control Order, that will provide an opportunity for countries to better manage the COVID-19 infection. Dr Tedros had also warned that ending the movement restrictions too early can result in a rebound and an increased rate of COVID-19 infection transmission in the country and within the community.<br>Active case detection via house-to-house testing for high risk areas where EMCO had executed.
Soo[174]	10/04/2020	"In terms of MCO, phase one and phase two, we have calculated our infectivity, and the R0 was 3.55, which means, when one person was infected, he will infect 3.55 people. That was the initial R0 before the MCO was enforced," he said.<br>"Now when you look at it today, we managed to bring down the R0, which means that the MCO phase one and two is very effective to bring down the R0.<br>"Because when everybody stays at home, the infection will be less, and the infectivity will be lesser as well," he added.<br>He said the MOH is now expecting the R0 brought down further to 0.9 on April 14, which as the original end date of the MCO. It has since been extended by two weeks to April 28.
Bernama[175]	10/04/2020	"MAEPS will receive low-risk Covid-19 patients from around the Klang Valley, Melaka and several areas in Perak," he said in an interview via Skype that broadcast live on Bernama 7.45.On the challenges faced throughout the process of transforming MAEPS into a quarantine centre in just a short period of time, Zaidi said it was difficult to get supplies from contractors as many were 'bound' by the movement control order (MCO)."We received the instruction during the first phase of the MCO but Alhamdulillah, with the close cooperation between various government agencies such as the Ministry of Health (MOH), NADMA (the National Disaster Management Agency), the Fire and Rescue Department and private companies, we managed to realise it within four days," he added.
Ministry of Health Malaysia[176]	11/04/2020	MOH will continue to give priority on the safety of HCWs, especially the front liners who continue to work hard in managing the COVID-19 situation in the interest of the nation.<br>Various measures have been taken to ensure continued safety and health of HCWs, including:

		Assessment of the needs and stocks of personal protective equipment (PPE) in all healthcare facilities to ensure adequate supply of PPE<br>All cases of SARI and pneumonia will be assumed as COVID-19 infection until proven otherwise<br>Strengthening triaging i.e. the evaluation and isolation of patients suspected of having COVID-19 symptoms when they attend healthcare facilities, and by providing them with facemasks<br>Ensuring that HCWs treating confirmed COVID-19 cases use the recommended PPE, while HCWs treating patients not suspected to be COVID-19 to wear facemasks<br>Ensuring that HCWs practice all precautionary and preventive measures such as hand hygiene and practice social distancing of at least 1 metre, based on needs and suitability<br>MOH strongly advises that all HCWs and front liners, including private medical practitioners and NGOs, must wear facemasks in managing and treating patients.<br>Some 110 Malaysians stranded in Pakistan due to flight cancellations and travel restrictions imposed to contain Covid-19 have safely returned home yesterday morning, said the Malaysian High Commission in Islamabad, Pakistan. The High Commission said all of them departed from Islamabad on the Pakistan International Airlines (PIA) flight referenced as PK894, and arrived at the KL International Airport (KLIA) at 11.20am (April 11).
Ministry of Health Malaysia[177]	12/04/2020	Madrasah (Religious School) Sub-Clusters in Several States - MOH has intensified activities for detection, screening, testing, isolation and treatment especially for high-risk groups via a targeted approach. These activities are done in collaboration with various government agencies and non-governmental organisations. For example, through close cooperation with the Department of Islamic Development, Malaysia (JAKIM) and the Majlis Syura Jemaah Tabligh (Tabligh Council) of Seri Petaling mosque, MOH was able to obtain the latest list of madrasah (religious schools) and students, and these have assisted in investigations and contact tracing of close contacts to the index cases of the Seri Petaling gathering cluster.<br>MOH urges the public to avoid blaming or discriminating against any patients and groups associated with the COVID-19 infection clusters. Do not stigmatise them. What is important is for all of us to help detect and treat COVID-19 infection amongst those involved, their family members and close contacts.<br>MOH advices the public to maintain a high level of hygiene at all times by regularly washing their hands with water and soap, and to always practise social distancing of at least 1 meter from others.
The Star[178]	12/04/2020	The Transport Minister said with this shipment, all 100 ICU beds ordered from Hebei Pukang Medical Treatment Equipment Co. Ltd, the largest medical equipment manufacturer in China, have arrived and distributed to government hospitals.<br>Dr Wee also thanked Yayasan Petronas for their generous donation in sponsoring the cost of the 100 ICU beds for the Health Ministry to treat Covid-19 patients.
Ministry of Health Malaysia[179]	14/04/2020	PPE is a key requirement for frontline HCW in the face of the COVID-19 pandemic. There are several types of PPE used by HCWs, for example: Footwear or boot cover – 78 days stock balance Protective head cover – 52 days stock balance 3-ply face mask – 47 days stock balance Surgical N-95 face mask – 37 days stock balance Disposable face shield (eye visor, face shield or goggles) – 25 days stock balance Disposable fluid resistant apron (long sleeves with cuffs) – 23 days stock balance Jumpsuit or protective coverall – 19 days stock balance Disposable plastic gown and other types of PPE To ensure that PPE stocks are sustainable, MOH has and will continue to carry out PPE procurement in bulk at central and

		state levels. MOH is working closely with the National Security Council, National Disaster Management Agency (NADMA) and other central agencies to ensure that issues relating to the stocks are specifically addressed. The contributions of PPE by various private entities, non-governmental organisations and philanthropists will continue to be welcomed and much appreciated. To closely monitor stock levels and consumption rates for each type of PPE in each state, MOH uses a simple online reporting and database system that is administered by Officers at all State Health Departments, hospitals and District Health Offices (including all health clinics under the respective District Health Office). This reporting system is closely monitored, and rapid mobilisation of stocks will be undertaken as required.
Ministry of Health Malaysia[180]	15/04/2020	Use of Disinfection Box, Chamber, Tunnel, Booth, Partition or Gate to Reduce COVID-19 Infection - MOH would like to inform that several endorsement applications have been received for the use of disinfection box/chamber/tunnel/booth/partition/gate to reduce COVID-19 infection, whereby their use have been evaluated by the Health Technology Assessment (HTA) Unit, MOH. This is because the use of such disinfection devices has been reported in several countries. Evaluations have been conducted on several models of such devices in several countries, as well as existing devices in Malaysia. It was found that the disinfection processes of these devices were evaluated involving a person passing through the device for a period of 20 to 30 seconds, during which the person is sprayed with disinfectant solutions. All the devices evaluated used various disinfectant solutions.
Ministry of Health Malaysia[181]	16/04/2020	Current status of PPE supply updated to the public. MOH continued PPE procurement in bulk at central and state level. The type of PPEs used must be in accordance with the duty requirements of the respective units where the HCWs are assigned to, as stated in the "Garis Panduan Pengurusan COVID-19 di Malaysia No.5/2020" or the COVID-19 Management Guidelines in Malaysia No.5/2020, in Annex 8: The Infection Prevention And Control (IPC) Measures (Refer Row 125, Gov ID 51). Not all HCWs are required to wear all types of PPE at their respective duty stations.<br>MOH worked closely with the National Security Council, National Disaster Management Agency (NADMA) and other central agencies to ensure that issues relating to the stocks are specifically addressed.<br>Contributions / Supply control and management<br>MOH sought contributions of PPE from various private entities, non-governmental organisations and philanthropists.<br>Monitoring of stock and consumption rate at state level done through online database.<br>Contact of responsible officers in each state made available to assist in providing feedback in the event of PPE shortage.<br>Immediate verification and rapid mobilisation of stocks will be undertaken based on needs and priorities<br>Contributions / Supply control and management<br>MOH sought contributions of PPE from various private entities, non-governmental organisations and philanthropists.<br>"The contributions of PPE by various private entities, non-governmental organisations and philanthropists will continue to be welcomed and much appreciated."
National Disaster Management Agency[182]	17/04/2020	MySejahtera is an application developed by the Government of Malaysia to assist in monitoring COVID-19 outbreak in the country by empowering users to assess their health risk against COVID-19.
Ministry of Health Malaysia[183]	17/04/2020	As reported by the mass media, there has been an increase in COVID-19 cases in Singapore associated with the spread of the infection in the workers' dormitories. This has contributed to over 50% of all COVID-19 positive cases in the country. Cognisant of this situation and given that Malaysia also have a large population of foreign workers, measures

		must be taken to prevent the spread of COVID-19 among this group of people.<br>MOH is also working closely with the embassies of relevant countries in Malaysia as well as relevant non-governmental organisations to assist MOH to share and disseminate information and health education messages to foreign nationals in Malaysia in their respective national languages.<br>MOH worked closely with the embassies of relevant countries in Malaysia as well as relevant non-governmental organisations to assist MOH to share and disseminate information and health education messages to foreign nationals in Malaysia in their respective national languages. The National IHR Focal Points from related countries were also notified of confirmed COVID-19 cases amongst foreign nationals in Malaysia.<br>For industries employing large numbers of workers, including foreign workers, employers are responsible for ensuring the following measures are implemented in the workplace:<br>- Screening for body temperature and symptoms before entering the premises or workplaces<br>- Provision of hand washing facilities and hand sanitisers<br>- Regularly carry out cleaning and disinfection, particularly in shared spaces<br>- Practice social distancing of at least 1 metre, in workspaces as well as in dining areas:<br>- Prevent employees from congregating in confined spaces<br>- Prevent the congregation of large numbers of workers<br>- Keep a distance of at least 1 metre during face-to-face conversations<br>When dormitories or living quarters are provided, preventive measures such as safe social distancing as well as personal and hand hygiene must be emphasised. Employees with their own accommodations must also comply and practise all of these preventive measures, as well as take their own initiatives to regularly clean and disinfect their own accommodations.
Prime Minister's Office of Malaysia[184]	17/04/2020	Yang di-Pertuan Agong Al-Sultan Abdullah Ri'ayatuddin Al-Mustafa Billah Shah today made a personal donation to the COVID-19 Fund.<br>Istana Negara, in a posting on its official Instagram page, said that the donation was presented to Prime Minister Tan Sri Muhyiddin Yassin through the Comptroller of the Royal Household of Istana Negara Datuk Ahmad Fadil Shamsuddin.<br>On March 31, Al-Sultan Abdullah and Raja Permaisuri Agong Tunku Hajah Azizah Aminah Maimunah Iskandariah agreed to forgo their Royal Emoluments for six months from March to August this year to ease the burden of the government in handling the COVID-19 pandemic in the country.
Tariq[185]	17/04/2020	The Malaysian Communications and Multimedia Commission (MCMC) has released a Covid-19 contact tracing app, Gerak Malaysia. The app is designed to help the authorities in tracing and analysing users' movement nationwide to contain the spread of the pandemic.
Ministry of Health Malaysia[186]	19/04/2020	As announced in a joint statement by MOH and the World Health Organization (WHO) on 6 April 2020, the "Solidarity Trial" launched by WHO will see Malaysia's involvement in an international multi-centre drug trial for the treatment of COVID-19. This worldwide WHO-coordinated research is on the efficacy and safety profiles of four treatment regimens using a combination of Remdesivir, Lopinavir/Ritonavir, Interferon beta, Chloroquine and Hydroxychloroquine.<br>In this regard, MOH will soon embark on this clinical trial whereby the nine (9) MOH hospitals identified to participate in the WHO Solidarity Trial are ready to begin recruitment of study participants amongst the COVID-19 patients who meet the study criteria.

Tan[187]	21/04/2020	Three ministries have entered into a tripartite collaboration to ensure that Malaysia gets the "first-mover advantage" to secure Covid-19 vaccines when they become available.<br>They are the Science, Technology and Innovation Ministry (Mosti), the Health Ministry (MOH) and the Foreign Ministry.<br>The joint effort to bolster Malaysia's chances at securing the vaccine is important, given that there are only a few pre-clinical and clinical trials for the vaccine currently taking place in several countries, said Mosti minister Khairy Jamaluddin.<br>He said MOH and Mosti were tasked with identifying the countries that were developing Covid-19 vaccines and medication.
Ministry of Health Malaysia[188]	22/04/2020	MOH calls upon all Muslims in the country to take advantage of the upcoming month of Ramadan to perform religious activities, prayers and preparing meals for breaking fast together as a family in their respective homes. In addition to celebrating the month of Ramadan with loved ones at home, this measure will help us to care and protect family members, particularly elderly parents and small children from being infected by COVID-19.<br>Malaysians are advised to continue to practice social distancing of at least one metre from others and maintain a high level of personal hygiene at all times, such as regular hand washing with water and soap.
Ministry of Health Malaysia[189]	24/04/2020	MOH recommends that everyone involved with the online food business, including business owners, food operators and food delivery providers to constantly maintain good personal hygiene and comply to all guidelines issued by the MOH (http://fsq.moh.gov.my/v6/xs/index.php). This is to ensure that the food provided to consumers are safe. In addition, they are also advised to practice social distancing during food delivery.<br>MOH would like to emphasize that the immunization program in all health facilities is running as usual.Parents are advised to adhere to the immunization schedule and appointment date set by the health care provider.Avoid from defaulting vaccination schedule.<br>Parents who have returned to the village should make an appointment for baby / child immunization at any nearby health clinic or private clinic. In our efforts to break the COVID-19 chain by complying with our efforts to break the COVID-19 chain by complying with MOH recommendations, childhood immunization needs to be continued to maintain herd immunity.
Ministry of Health Malaysia[190]	27/04/2020	Hire Specialist and Medical Officers from private healthcare sectors by contract
Social Security Organisation[191]	27/04/2020	SOCSO PSP launched on 27 April 2020 is a COVID-19 screening program conducted to foreign workers in the construction and security securities sectors control subject to conditions provided by SOCSO.SOCSO PSP is dedicated to foreign workers who contribute to SOCSO and work in the construction and security services sectors in Selangor and Federal Territory of Kuala Lumpur only. Screening is focused on both sectors is based on a higher risk of COVID-19 infection in these sectors and also in accordance with the Government's directive to conduct targeted screening.
Bernama[192]	28/04/2020	The Ministry of Health today (MOH) receives a personal contribution of two million rubber medical gloves from the Sultan of Kelantan, Sultan Muhammad V for use by frontline workers in the war against Covid-19.
New Straits Times[193]	28/04/2020	A total of 125 Malaysians flew home on Tuesday from Kualanamu International Airport in Dell Serdang regency, North Sumatra. According to reports in the Jakarta Post, 69 Indonesians arrived at the airport at 4pm on a Malaysia Airlines flight and 45 minutes later, the Malaysians flew home on the same aircraft.

New Straits Times[194]	01/05/2020	Foreign travellers – with the exception of diplomats, permanent residents and expatriate pass holders working in essential services sectors – are still barred from entering Malaysia despite the relaxation of regulations with the implementation of the Conditional Movement Control Order (CMCO) beginning Monday.<br>Exempted foreigners, meanwhile, must undergo 14-day self-quarantine upon arrival in Malaysia.<br>This was confirmed in a brief guideline by the National Security Council (NSC) following Prime Minister Tan Sri Muhyiddin Yassin's announcement today of the revised standard operating procedure (SOP) for the CMCO.
Prime Minister's Office Ministry of Health Malaysia of Malaysia[195]	01/05/2020	Most of economic activities allowed to operate, subject to conditions and SOPs set by the authorities
Ministry of Health Malaysia[196]	02/05/2020	The MCO has enabled the implementation of the targeted approach to detect, screen, test, isolate and treat COVID-19 cases in the community. The effectiveness of this approach is evident when we look at statistics of the implementation i.e.:<br>The Enhanced MCO (EMCO) in 11 high-risk localities, whereby 607 confirmed COVID-19 cases were detected from 29,067 individuals tested (2.1%)<br>Case Detection related to the Seri Petaling gathering cluster, whereby 2,220 confirmed COVID-19 cases were detected from 34,503 individuals tested (6.4%)<br>Detection of cases amongst students and staff of the madrasah (religious schools) associated with the Seri Petaling gathering, whereby 378 confirmed COVID-19 cases were detected from 8,030 individuals tested (4.7%)<br>Screening and placement in quarantine centres for all Malaysians returning from overseas since 3 April 2020, whereby 274 confirmed COVID-19 cases were detected from 26,169 quarantined individuals (1.05%).<br>The recent trend of confirmed COVID-19 cases reported daily has shown an increase in imported cases and cases detected from EMCO localities. However, the public does not have to worry of increasing cases amongst imported or EMCO localities because these cases are isolated early for screening.<br>Local transmission of COVID-19 can be detected through the long-standing COVID-19 infection clinical surveillance. This is done through sampling of Influenza Like Illness (ILI) patients from sentinel locations nationwide and Severe Acute Respiratory Infection (SARI) patients in all MOH hospitals.
Ministry of Health Malaysia[197]	04/05/2020	As announced by the Prime Minister of Malaysia, the implementation of the Conditional Movement Control Order (CMCO) is effective from today, 4 May 2020.<br>As stated earlier, the Government has decided to implement the CMCO so as to re-open several sectors of the economy in a cautious and controlled manner. Business owners, traders and employers must comply with all of the requirements and Standard Operating Procedures (SOPs) set by the authorities. Public gatherings and activities that expose the public to the risk of COVID-19 infection are still prohibited.<br>While business owners, traders and employers must comply with all SOPs set by the Government, the population living in the States and Territories undergoing the CMCO are urged to also comply with THiS:<br>T: Terms and SOP set<br>Hi: High-risk groups such as children, infants, older adults and the disabled must be protected, and if unwell with

		symptoms, to seek early treatment<br>S: Safe social distancing is practiced at all times<br>MySejahtera enables users to perform self-health-assessment in addition to assisting MOH to obtain early information on the individual's health status to enable prompt and effective action.<br>The functionalities of MySejahtera include:<br>Hotspot tracker that allows users to obtain information on COVID-19 case hotspot area within one (1) kilometer of the user's location or search address<br>Provides information on the nearest COVID-19 screening facility and online advice assistance<br>In addition to personal use, users can also register information of family members who do not own smartphones, and this would enable MySejahtera to assess the health status of these family members as well. Users who have downloaded this application will need to update to the latest version to get the full benefit of MySejahtera. This application will be improved with the latest features and updated from time to time.<br>MOH urges all Malaysians as well as non-Malaysians in the country to download the MySejahtera application, and to conduct self-assessment using this application to identify their risk of COVID-19 infection.<br>In addition, as announced by Ministry of Science, Technology and Innovation (MOSTI) has developed the MyTrace application. This application enables the Government to conduct contact tracing on individuals who are detected to be positive for COVID-19. MyTrace is a complement to the MySejahtera application.
Ministry of Health Malaysia[106]	05/05/2020	As announced by the Senior Minister (Security Cluster) on 5 May 2020, the Government has set up a Compliance Operations Task Force. This Task Force comprises of 14 agencies for monitoring and enforcing compliance with the SOPs of the MCO throughout the country. Some of the agencies involved are: Royal Malaysian Police (PDRM), Malaysian Armed Forces (ATM), Civil Defence Forces (APM), Malaysian Maritime Enforcement Agency (APMM)Department of Labour, Department of Occupational Safety and Health, Public Works Department (JKR), Ministry of International Trade and Industry, Immigration Department of Malaysia, Ministry of Plantation Industries and Commodities, Malaysian Bio-Fuel Industry, Malaysian Palm Oil Board, Malaysian Rubber Board, Malaysian Timber Industry Board, Ministry of Education Malaysia, Hospital Directors, Construction Industry Development Authority of Malaysia (CIDB) and Public Works Department (JKR) Sarawak
Minderjeet[198]	05/05/2020	Authorities have placed two areas in Kuala Lumpur under a "semi-enhanced" movement control order (SEMCO), to allow mass testing on residents there. The affected areas are Taman Wilayah and Taman Desa Bakti in Selayang, and parts of the Chow Kit area along Jalan Raja Bot in the city centre. But Deputy Federal Territories Minister Edmund Santhara said people would still be allowed to move in and out of the area if they had valid reasons. "Businesses can operate as usual," he told FMTHe said the latest move was to allow authorities to conduct tests on all residents and foreign workers. Under SEMCO, 10% of residents will be sampled, with road blocks to monitor people's mobility.
Bunyan[199]	05/05/2020	1. Gerak Malaysia Developed by the Malaysian Communications and Multimedia Commission (MCMC), this app is designed to help the police and MOH in tracing and analysing users' movement nationwide to contain the spread of the pandemic. Users also can register or apply for interstate travel via the app instead of applying manually at police stations.<br>2. MySejahtera Jointly developed by four government bodies, including the National Security Council (NSC) and the Ministry of Health (MOH), this is a one-stop app to get the latest information and statistics on the pandemic. Useful for

		Persons Under Surveillance or those who are quarantined during the MCO, it has a self-evaluation feature, where one needs to fill up questionnaire on their current health status and once completed, a result will be provided as to whether the person is at risk of being a patient. The app also informs users of what action need to be taken if a person is infected with Covid-19, and has a tracker to track Covid-19 hotspots. The MOH has access to your data on the app, which allows it to take action if a possible infection has been identified.<br>3. MyTrace Developed by MOSTI, this is a contact tracing app that uses Bluetooth to measure how long a user's phone has been in proximity with other MyTrace users. If a user is diagnosed with Covid-19, this would allow the authorities to track and contact those who might have contracted it from them. Data can also be shared with the MySejahtera app, if a user has both installed on their phones.
CodeBlue[200]	06/05/2020	"As I mentioned, our focus now is on targeted areas. If we look back, Petaling is of course considered a red zone, because two areas are red–Sri Petaling is red and Lembah Pantai is red," said Ismail Sabri at a press conference today."But our focus isn't like before, not around Petaling, but in areas that are identified. And if there is a sudden increase in cases, we will enforce administrative MCO before CMCO, and if there is an increase in cases again, then we will enforce EMCO (Enhanced Movement Control Order)."
PGCare[201]	08/05/2020	1. What is the PGCare initiative? PGCare is developed as part of the "Penang Gradual Recovery Strategy (PGRS)" to ensure the health and safety of the people of Penang. PGCare is a digital logbook system that allows anyone to quickly check-in through QR code that will be displayed within a premise. This ensures businesses in Penang can run efficiently while strictly adhering with the standard operating procedures of the Security Council as more sectors are allowed to operate from May 8, 2020.<br>2. What are the objectives of PGCare initiative? Establishing an efficient preventive measure that will allow health authorities to quickly trace every contact, protecting our frontliners Empowering local community to adapt to the new normal Ensuring the continuity and of the state's economic sectors<br>3. How can PGCare help the State Health Department? With PGCare, the State Health Department's Contact Tracing effort can be conducted swiftly with accurate and up-to-date visitor information whenever there is a reported positive Covid-19 case occurrence located near any business premise (For example: Shopping malls, restaurants or grocery stores registered with PGCare). With fast and accurate data from PGCare, visitors to an affected business premise can be contacted by the health authorities to ensure appropriate tests can be conducted within the shortest time possible.<br>4. How is PGCare different from existing methods or platforms? Visitors to a business premise registered with PGCare are only required to sign up with their details via a simple form. Returning visitors can effortlessly check in to the same or different location by scanning a PGCare' QR code displayed around the premise. This ensures that visitor registration can be conducted smoothly and efficiently by all businesses.
Ministry of Health Malaysia[202]	10/05/2020	The Target Approach Activities Showing Positive Results So far, the implementation of the targeted approach has shown a positive impact in detecting additional cases of COVID-19. As of 10 May 2020, MOH has identified several target populations, such as: Participants of the Seri Petaling gathering, and their close contacts Students and staff of the madrasah and tahfiz (religious schools) closely associated with the Seri Petaling gathering Malaysians returning from overseas, and being housed in quarantine centres MOH healthcare workers (HCWs) Residents of EMCO localities or areas Pasar Borong (Wholesale Market) Kuala Lumpur (PBKL) and wet markets closely linked to the PBKL Foreign workers at construction sites in red zones Senior citizen care homes The public does not need to worry about the

		additional cases reported following the Government's aggressive and proactive actions, as this demonstrates the effectiveness of these activities. The cases detected have been isolated and hospitalised to ensure that the COVID-19 virus do not continue to spread in the community.
Prime Minister's Office of Malaysia[203]	10/05/2020	Under advices of MOH and MSC, I would like to announce that CMCO would be enforced until 9 June
Ministry of Health Malaysia[204]	15/05/2020	List of screening & triage booth for private hospitals, ambulatory care centres and clinics approved by MOH
Ministry of Health Malaysia[205]	20/05/2020	MOH urges the public to donate blood at the National Blood Centres and other Blood Centres throughout the country so that the blood supply would be continuous and adequate. Operations of the National Blood Centres and other Blood Centres have been extended until 9 pm throughout the month of Ramadan to enable individuals to donate after breaking fast.
Ministry of Health Malaysia[206]	22/05/2020	A month of fasting during the month of Ramadan during the COVID-19 pandemic has taught us all the true meaning of patience and sacrifice. Ramadan was experienced under new norms and practices to ensure that COVID-19 infection can be prevented. MOH is now calling on the public to celebrate Hari Raya Aidilfitri and the month of Syawal with new norms and practices, in accordance with all published Standard Operating Procedures (SOPs).<br>The celebration of Hari Raya Aidilfitri is not complete without the Hari Raya special food and drinks. This is indeed the normal practice of all Malaysians on Hari Raya. In order to ensure that the food and drinks can be enjoyed safely, the process of preparing food and meals should be given attention especially in terms of proper and safe handling and cooking.
Povera and Yunus[207]	28/05/2020	The government has strengthened enforcement along the country's borders, especially in areas where there is a risk of having 'lorong tikus' (illegal routes).<br>Senior Minister (Security Cluster) Datuk Seri Ismail Sabri Yaakob said this was done to stop illegal immigrants from neighbouring countries struggling with the global Covid-19 pandemic from entering Malaysia.<br>He said the decision to intensify 'Op Benteng' would also help the government to prevent the emergence of any imported cluster of Covid-19 from those affected countries, which has seen a declining trend in the number of daily new cases.<br>Op Benteng is an integrated operation involving the Malaysian Armed Forces, police, Malaysian Maritime Enforcement Agency and Malaysian Border Security Agency to enhance enforcement and control along the country's borders.
Ministry of Health Malaysia[208]	29/05/2020	MOH welcomes the announcement by the Minister of Human Resources on 27 May 2020, on the enforcement of the amendment to the Workers' Minimum Standards of Housing and Amenities Act 1990 (Act 446). This amendment is to improve the Guidelines on Foreign Workers' Accommodation 2018 published by the Department of Labour of Peninsular Malaysia.<br>This issue needs to be addressed immediately because the current condition of the workers' living quarters makes it difficult to comply to social distancing practices. In addition, employers are also responsible to provide health education to their workers on good hygiene practices, prevention of COVID-19 infection in the living quarters, as well as compliance with the various SOPs developed by the Government for the industries, including the workers' movements.

New Straits Times[209]	03/06/2020	The three neighbourhoods in the Kuala Langat district which have been cordoned off by the authorities since last night are not placed under Enhanced Movement Control Order (EMCO).Unlike areas placed under EMCO, residents of Taman Langat Utama, Taman Langat Murni 1 and Taman Langat Murni 2 are free to move about but they are subjected to undergo Covid-19 screening by the Health Ministry. Senior Minister (Security Cluster) Datuk Seri Ismail Sabri Yaakob said this when he was asked to comment on the Movement Control Order (MCO) under administrative control being enforced at the three residential areas. "As I mentioned previously, there are two types of directives including the EMCO which were previously enforced for areas in Selangor Mansion and (Kuala Lumpur) wholesale (market in Selayang)."The other type of directive is the Movement Control Order (MCO) under administrative control in which people are still free to move about although barbed wire fencing is being installed at the area similar to localities placed under EMCO," the Defence Minister said during his non-health related press conference, here, today.Similar to EMCO, Ismail Sabri said all residents staying at areas placed under the administrative control would also be tested for Covid-19.
Ida Nadirah,I[210]	05/06/2020	The Selangor government has merged its SELangkah Covid-19 contact tracing mobile application with the MySejahtera application developed by the Federal administration.
Prime Minister's Office of Malaysia[211]	07/06/2020	Alhamdulillah, since MCO and CMCO were implemented, the rate of COVID-19 infection in our country has shown a declining trend. Statistics from the Ministry of Health show that the rate of infection among locals is getting lower and more controlled. Most of the cases recorded recently are import cases and cases involving illegal immigrants in immigration detention depots. We are thankful that the swift action of the frontline officers has succeeded in curbing the spread of COVID-19 among the detainees at the immigrant depot and the situation is currently under control. At the same time, our country's medical and public health capacity is also at a better and more convincing level. This shows that Malaysia has successfully curbed the transmission of COVID-19 effectively and is now entering the recovery phase.
Ministry of Health Malaysia[212]	07/06/2020	Previously, the Government had adopted an aggressive approach to enforce the Movement Control Order (MCO) on 18 March 2020, and subsequently followed by the Conditional Movement Control Order (CMCO) since 4 May 2020, during which several economic sectors were allowed to operate in accordance with the set Standard Operating Procedures (SOPs).<br>The enforcement of the RMCO starting on 10 June 2020 is step-wise implementation by the Government to ensure that the country can go through the recovery phase in this COVID-19 pandemic. More economic sectors, social, educational, religious and business activities will be allowed. The movements of the public will also be freer. All these will be implemented in accordance with the SOPs set by the Government. It is hoped that this approach will balance the risks between lives and livelihoods, as part of the soft-landing approach in preparing the country for the exit strategy.
Ministry of Health Malaysia[213]	10/06/2020	Starting 10 June 2020, individuals returning from overseas will have to undergo home quarantine. These individuals will be placed under monitoring and surveillance or Home Surveillance Order (HSO) for 14 days according to the MOH guidelines. This Monitoring and Surveillance Order is under Section 15(1) of the Prevention and Control of Infectious Diseases Act 1988 [Act 342]. Individuals are also required to wear the HSO bracelet for the full duration of the Monitoring and Surveillance Order.
Ministry of Health Malaysia[214]	15/06/2020	MOH would like to announce that today is the last day of MOH's press conference daily live broadcasts. Starting this week, the press conference will only be held three times a week, on Mondays, Wednesdays and Fridays.

Ministry of Health Malaysia[215]	19/06/2020	Clinical surveillance is done by random COVID-19 sampling from MOH healthcare facilities. Up till the 24th epidemiological week (i.e. up to 13 June 2020), a total of 19,309 samples of Influenza Like Illness (ILI) and Severe Acute Respiratory Infection (SARI) cases were taken, of which:<br><br>ILI cases: 15 confirmed COVID-19 cases out of 2,048 samples (a positive rate of 0.7%)<br>SARI cases: 76 confirmed COVID-19 cases out of 17,261 samples (a positive rate of 0.4%)<br>MOH would like to inform that the number of sentinel health clinics has been increased to a total of 26 health clinics throughout the country. This is to strengthen the early warning system for COVID-19 infection transmission in Malaysia.<br>For pre-surgical screening (emergency and semi-emergency cases) at MOH hospitals; up till 19 June 2020, a total of 17 confirmed COVID-19 cases were detected from 37,756 patients screened (a positive rate of 0.05%).<br><br>As part of the targeted approach strategy, targeted screening is done for nine (9) identified high-risk groups, as follows:<br><br>Seri Petaling gathering: 3,375 confirmed COVID-19 cases out of 41,955 individuals screened (a positive rate of 8.04%)<br>Religious schools: 722 confirmed COVID-19 cases out of 19,050 individuals screened (a positive rate of 3.79%)<br>Healthcare workers: 363 confirmed COVID-19 cases out of 28,512 individuals screened (a positive rate 1.27%)<br>Locations under all types of Enhanced Movement Control Order (EMCO): 913 confirmed COVID-19 cases out of 31,258 individuals screened (a positive rate of 2.92%)<br>Travellers from overseas (since 3 April 2020): 598 confirmed COVID-19 cases out of 51,121 individuals screened (a positive rate of 1.17%)<br>Wet markets: 275 confirmed COVID-19 cases out of 34,028 individuals screened (a positive rate of 0.81%)<br>Non-Malaysian workers at construction sites and the security sector (in Red Zones): 154 confirmed COVID-19 cases out of 1,696 individuals screened (a positive rate of 9.08%)<br>Senior care facilities: 30 confirmed COVID-19 cases out of 16,213 individuals screened (a positive rate of 0.19%)<br>Immigration Detention Centres: 776 confirmed COVID-19 cases out of 8,280 individuals screened (a positive rate of 9.37%). Of these, 83% are from the Bukit Jalil Immigration Detention Centre.<br>COVID-19 surveillance in Malaysia will continue even when the Recovery Movement Control Order (RMCO) ends. In addition, the surveillance will be strengthened including in the private sector. MOH would like to inform that all confirmed COVID-19 cases in the public (government) and private healthcare facilities must by notified to the MOH. This is because COVID-19 has already been listed as a notifiable disease under the Prevention and Control of Infectious Diseases Act 1988 [Act 342].
Ministry of Health Malaysia[216]	26/06/2020	COVID-19 test performed all detainees and prisoners before they were sent into or sent out from detention centres or prisons
Ministry of Health Malaysia[217];	30/06/2020	As of 30 June, there were 764,999 individuals sampled, with positive rate of 1.13% (8639 positive cases). Total population in Malaysia - 32.7 millions, equivalent to 23,394 COVID-19 test per one million population

Department of Statistics Malaysia[218]		
Yusof et. al.[219]		Since the beginning of the COVID-19 pandemic, Malaysians have witnessed a series of patients' personal information being leaked into social media. For instance, a patient known as 'Patient 16' had become a target of hate and discrimination after his name and profile picture were widely circulated on social media, which continued even after he was cleared of the virus
Khoo et. al[220]		The National Institute of Forensic Medicine (IPFN) Malaysia has also produced a guideline on management of the dead for all COVID-19 suspected or confirmed deaths within the hospital as well as from outside the hospital (brought-in-dead cases by police). This guideline consists of various sections including guidelines on body transport from Emergency Departments or wards to the mortuary, postmortem examination, handling of the body and religious/ritual body preparation and disposal.<br>In this preparation phase, the IPFN Malaysia has taken the initiative to engage the Royal Malaysia Police (RMP) to create awareness on the risk of COVID-19 infection and safety precautions that must be adhered prevent infection to all RMP ground officers during the investigation of a body found at the scene. Training sessions were also given to the RMP on proper hand hygiene and washing, donning and doffing of the standard full personal protective equipment (PPE) which consists of a surgical mask, double surgical gloves, face shield, impervious gown or apron with full sleeve coverage, shoe covers and surgical cap [10]. Concurrently, personnel working in the mortuary are given refresher hands-on and practical training on the use of the powered air-purifying respirator (PAPR) as well as donning and doffing of the standard full PPE in all forensic medicine centers in the country.<br>COVID-19 centralized body facility This type of facility, identified by NADMA and situated at various locations, functions as temporary mortuary centre for all COVID-19 deaths in the country. It is a holding area for all the dead whereby its security is maintained by the RMP, APM or ATM and is equipped with cold body storage containers in which bodies are properly tagged, documented and stored.
Tang and Ho[221]		The Malaysian government has been vigilant on Covid-19 since January 2020, starting with issuance of advice against travelling to China on 25 January after three Chinese citizens were tested positive for Covid-19 (Immigration Department of Malaysia 2020). After that, a series of measures has been rolled out as summarized in Table Table1.1. The measures started with entry restriction of selective foreign nationals into Malaysia, as well as self-quarantine of Malaysian and non-Malaysian citizens returning from Covid-19 hotspots, and culminated with the passing and execution of the Movement Control Order (MCO) (Immigration Department of Malaysia 2020; Ministry of Health 2020; Prime Minister's Office of Malaysia 2020a).
Umair et. al.[222]		COVID-19 pandemic: stringent measures of Malaysia and implications for other countries<br>'Resolve, resilience, restart, recovery, revitalise and reform' is the new normal for the Malaysian people.13 It is a sixstep plan by the Malaysian government to address the impact of COVID-19 and to make sure that the country emerges stronger despite this virus. Using this six-step plan, Malaysians try to adhere to new standards of operating procedures (SOPs) to prevent another wave of infections in the country and to revive the economy in stages. Despite the loss of millions of dollars, they still have imposed conditional MCO, whereby economic sectors have been allowed to open with enforced guidelines; however, interstate travels are still banned. Red zone areas are strictly being monitored. The

		education sector has already been shifted to home-based learning in the country. Cinemas, sports competitions, leisure clubs, weddings, social events and theme parks will remain closed. There are special SOPs for houses of all worship faiths. Any changes to the SOPs would be announced from time to time by the government.
Elengoe[223]		2.1. Treatment of COVID-19 in Malaysia A person was "suspected" of having COVID-19 based on the criteria below [21]. Has acute respiratory infection (either with difficulty in breathing, sore throat, or dry cough) with or without fever AND Had a history of travel to or resided in a foreign country within 14 days before onset of disease OR Had close contact with an infected person in the last 14 days before the onset of symptoms Attended a gathering or event linked to a COVID-19 outbreak A person was "confirmed" COVID-19 positive after performing laboratory tests. COVID-19 patients can be diagnosed using diagnostic testing kits for the presence of the virus. Imaging techniques such as chest X-ray and pulmonary CT scans can be used to diagnose pneumonia in COVID-19 patients. There are 5 clinical stages of COVID-19 [21]. Stage 1. asymptomatic, Stage 2. symptomatic and no pneumonia, Stage 3. symptomatic and pneumonia, Stage 4. symptomatic, pneumonia and supplemental oxygen required, Stage 5. critically ill with multiorgan failure Currently, there is no vaccine or specific treatment for COVID-19 that has been approved for use in humans. In Malaysia, hydroxychloroquine has been proposed as a drug to treat patients with COVID-19. Hydroxychloroquine inhibits endocytic pathways by elevating the pH of the endosomes to block the pH-dependent entry of the virus into the host cell [45]. The suggested treatment regime using hydroxychloroquine is Stage 1. No treatment required Stage 2. Hydroxychloroquine only Stage 3/4. Hydroxychloroquine combined treatment with Lopinavir/Ritonavir Stage 5. Hydroxychloroquine combined treatment with Lopinavir/Ritonavir, Ribavarin or Interferon Beta [21].
Yan et. al.[224]		Medical teams in China [13], Singapore [99], Malaysia [100], and Thailand [97] have observed promising e<br>ects of KALETRA in combination with other medical interventions to treat COVID-19 patients, leading to many being discharged from hospitals.
Tan et. al.[225]		The core highlights of these strategies enforced during this pandemic are: (1) surgery ward and clinic decongestions; (2) deferment of elective surgeries; (3) restructuring of medical personnel work force; (4) utilization of online applications for tele-communication; (5) operating room (OR) adjustments and patient screening; and (6) continuing medical education and updating practices in context to COVID-19.
Tay et.al. [226]		The most fully equipped, dedicated OTs for COVID-19 patients /Person under Investigation (PUI) were immediately made available. The OT chosen ideally is one with negative air pressure or one that will minimise OT contamination, staff exposure and nearest to the entry point. The non- COVID operation theatres were further divided into Emergency and Semi-Emergency OT. All Electives cases were being postponed indefinitely. Surgical management of emergencies such as fractures, dislocations, tumours and infections might still be undertaken and surgeons must adhere to the recommendations of the Surgical and Orthopaedic Association regarding this. The emergency and semi-emergency cases were required to be tested for COVID-19. Even if they were tested negative, the non- COVID cases would still be handled by health care workers (HCW) in full Personalised Protective Equipment (PPE). This is to protect the HCW against "false negative cases
Cheong et. al.[227]		Through collaborative discussions with other members of the health care team, clinical pharmacists in Malaysia have been able to continue providing care through the following approaches which seek to optimize service delivery while minimizing the spread of COVID-19: • Remote screening of medication charts to identify medication-related problems and phone calls/text messaging to prescribers to address these problems • Arranging for short video calls or

		teleconferences after physicians complete their rounds to get updates on the clinical status of select patients in the ward • Filming videos of themselves giving instructions on how to use medical devices so that nurses can show these videos to patients, in lieu of pharmacist counselling • Providing round-the-clock drug information services via phone/email to the physicians they usually work with • Providing updates on the latest evidence to guide COVID-19 management to other members of the health care team.(9)
Shah et. al.[228]		Mainstream and social media COVID-19 has caused fear, anxiety, and confusion. The media, celebrities, and other influencers have appealed to the public to stay at home and avoid mass gatherings. The media has started to use the hashtag #stayhome. This hashtag has been used widely in the media, and it is hoped that important messages to stop the spread of COVID-19 can reach all levels of society.<br>The MCO has affected Malaysia's economy. Most companies have their employees working from home, and some workers have had to stop working. NGOs have been actively helping those who are affected by this pandemic. They have been providing food, shelter for the homeless, and have even given out money to help those in need. Some NGOs have helped by providing protective masks, disinfection chambers (Bernama, 2020c), and helping to educate citizens on COVID-19 (The Star, 2020c). All frontliners are required to wear PPE. However, due to the rapid increase in COVID- 19 cases, there have been shortages in PPE equipment. This shortage could have endangered the health of frontliners. Therefore, several NGOs and public figures have helped to sew PPE for medical frontliners. For example, several Malaysian fashion designers associated with the Malaysian Official Designers Association (Moda) have produced PPE for local medical staff (Cheong, 2020). Prison inmates have also been involved in sewing protective gear for frontliners regardless of their current situation (Chalil, 2020). Volunteer tailors have helped to prepare PPE for frontliners.

1. Kenyataan Akhbar KPK 15 Februari 2020 – Situasi Terkini Jangkitan 2019-NCoV dan Pengesanan Kes Baharu di Malaysia [Ministry of Health Press Statement on 15 February 2020 - Updates On The Coronavirus Disease 2019 (COVID-19) Situation In Malaysia] [press release]. Malaysia: Ministry of Health Malaysia, 15 February 2020.
2. Kenyataan Akhbar YBMK 28 Januari 2020 - Situasi Semasa Jangkitan 2019-nCoV di Malaysia dan Kesiapsiagaan KKM Menghadapi Penularannya

[Ministry of Health Malaysia Press Statement on 28 January 2020 - Updates On The Coronavirus Disease 2019 (COVID-19) Situation In Malaysia and MOH Preparedness in Facing the Outbreak] [press release]. Malaysia: Ministry of Health Malaysia, 28 January 2020.
3. Kenyataan Akhbar KPK 6 Januari 2020 – Makluman Kejadian Kejadian Kluster Radang Paru-Paru (Pneumonia) di Wuhan, Republik Rakyat China [Ministry of Health Malaysia Press Statement on 6 January 2020 - Updates On The Occurence of Pneumonia Clusters in Wuhan, People Republic of China Situation] [press release]. Malaysia: Ministry of Health, 6 January 2020.
4. Minister of Health Malaysia. Kenyataan Akhbar KPK 16 Januari 2020 – Kesiapsiagaan dan Respons KKM dalam Menghadapi Potensi Penularan Novel Coronavirus, Wuhan, China [Press Statement MOH 16 January 2020- Preparedness and Responds MOH in Facing the Potential Outbreak of Novel Coronavirus, Wuhan, China] Malaysia: Ministry of Health; 2020 [cited 17 May 2020]. Available from:

https://kpkesihatan.com/2020/01/16/kenyataan-akhbar-kpk-16-januari-2020-kesiapsiagaan-dan-respons-kkm-dalam-menghadapi-potensi-penularan-novel-coronavirus-wuhan-china/.
5. Minister of Health Malaysia. Makluman Kejadian Jangkitan Novel Coronavirus (nCoV) di Wuhan, Republik Rakyat China dan Langkah Kesiapsiagaan bagi Menghadapi Potensi Penularannya di Malaysia [Notification of Novel Coronavirus (nCoV) Disease in Wuhan, People Republic of China and Preparedness Steps to Face its Potential Outbreak in Malaysia] Malaysia: Ministry of Health; 2020 [updated 17 January 2020. Available from: https://www.moh.gov.my/moh/resources/Penerbitan/Garis%20Panduan/Pengurusan%20KEsihatan%20&%20kawalan%20pykit/Surat_Makluman_dan_interim_guideline_nCoV.pdf.
6. Laboratory Readiness for Detecting the 2019 novel coronavirus (2019-nCoV) infection in Malaysia [press release]. Malaysia: Ministry of Health, 13 February 2020.
7. Kenyataan Akhbar KPK 23 Januari 2020 - Kemaskini Situasi Semasa Wabak Jangkitan 2019 Novel Coronavirus (2019-Ncov) dan Status Kesiapsiagaan Bagi Menghadapi Potensi Penularannya di Malaysia [Press Statement MOH 23 January - Updates on Situation During Outbreak of Novel Coronavirus 2019 (2019-Ncov) and Preparedness Status to Face Its Outbreak Potential in Malaysia] [press release]. Malaysia: Ministry of Health Malaysia, 23 January 2020.
8. Kenyataan Akhbar KPK 24 Januari 2020 – Tindakan KKM Bagi Pengesanan Kontak (Contact Tracing) kepada Kes Pertama Positif Novel Coronavirus di Negara Singapura pada 24 Januari 2020 [Ministry of Health Malaysia Press Statement on 24 January 2020 - MOH Actions for Contact Tracing on the First Positive Case of Coronavirus Novel in Singapore on 24 January 2020] [press release]. Malaysia: Ministry of Health Malaysia, 24 January 2020.
9. Ministry of Health Malaysia. Keratan Akhbar Situasi Terkini 25 Januari 2020 [Newspaper Clippings of Current Situation 25 January 2020] Malaysia: Ministry of Health; 2020 [cited 14 May 2020]. Available from: http://covid-19.moh.gov.my/terkini/012020/situasi-terkini-25-jan-2020/Keratan%20Akhbar%2025%20JAN%202020-min.pdf.
10. Kenyataan Akhbar KPK 25 Januari 2020 – Pengesanan Kes Baharu yang Disahkan Dijangkiti 2019 Novel Coronavirus (2019-nCoV) di Malaysia [Ministry of Health Malaysia Press Statement on 25 January 2020 - Detection of New Confirmed Cases of Novel Coronavirus 2019 (2019-nCoV) in Malaysia] [press release]. Malaysia: Ministry of Health Malaysia, 25 January 2020.
11. Loheswar R. Health Ministry confirms three cases of coronavirus infection in Malaysia. Malay Mail. 2020 25 January 2020.
12. Kenyataan Akhbar KPK 26 Januari 2020 – Situasi Terkini Jangkitan Novel Coronavirus di Malaysia [Ministry of Health Malaysia Press Statement on 26 January 2020 - Updates On The Coronavirus Disease 2019 Situation In Malaysia] [press release]. Malaysia: Ministry of Health Malaysia, 26 January 2020.
13. Thomas J. Don’t panic, we have outbreak under control, says Wan Azizah. Free Malaysia Today. 2020 26 January 2020.
14. Palansamy Y. Health Ministry and Home Ministry to meet National Disaster Agency following coronavirus outbreak. Malay Mail. 2020 26 January 2020.
15. Yusof TA, Landau E. Visa facilities suspended for Wuhan, Hubei tourists. New Straits Times. 2020 28 January 2020.

16. Immigration Department of Malaysia. Expatriate Application For People Republic Of China (PRC) Passport Issued In Wuhan City, Hubei Province Of China Malaysia: Immigration Department of Malaysia; 2020 [updated 29/01/2020. Available from: https://esd.imi.gov.my/portal/latest-news/announcement/announcement-for-coronavirus-outbreak/.
17. New Straits Times. Travel suspension will cost millions 2020 [cited 2 August 2020]. Available from: https://www.matta.org.my/news/61555-travel-suspension-will-cost-millions.
18. Ong J. Putrajaya rushes funds for heat scanners as Wuhan virus risks persist. 2020 28 January 2020.
19. Kenyataan Akhbar KPK 29 Januari 2020 – Situasi Terkini Jangkitan 2019-nCoV di Malaysia dan Kesiapsiagaan KKM Menghadapi Penularannya [Ministry of Health Malaysia Press Statement on 29 January 2020 - Updates On The Coronavirus Disease 2019 (COVID-19) Situation In Malaysia and MOH Preparedness Facing its Transmission] [press release]. Malaysia: Ministry of Health Malaysia, 29 January 2020.
20. Mok O. Penang CM: No reports of coronavirus in state, tourism sites remain open. Malay Mail. 2020 29 January 2020.
21. Palansamy Y. PM: Govt to discuss with China to bring back Malaysians trapped in Hubei province. Malay Mail. 2020 29 January 2020.
22. Tawie S. Sarawak police initiate investigations against spreading of fake news on coronavirus. Malay Mail. 2020 29 January 2020.
23. Ben T. Johor exco says Kukup ferry passengers screened for coronavirus, all clear to date. Malay Mail. 2020 10 February 2020.
24. Minister of Health Malaysia. Pengecualian Caj Perubatan Kepada Pesakit Warga Asing Yang Disyaki / Dijangkiti 2019 nCoV Serta Kontak Kepada Pesakit Yang Disyaki Dan Disahkan Dijangkiti 2019 nCoV [Exemption from Medical Charges for Suspected / Infected Foreign Patients 2019 nCoV As well as Contact For Suspected And Confirmed Cases 2019 nCoV] Malaysia: Minister of Health Malaysia; 2020 [updated 29 January 2020; cited 20 June 2020]. Available from: http://www.moh.gov.my/index.php/database_stores/store_view_page/10/375.
25. Kenyataan Akhbar YBMK 30 Januari 2020 - Situasi Terkini Jangkitan 2019 Novel Coronavirus (2019-nCoV) di Malaysia [Ministry of Health Malaysia Press Statement on 30 January 2020 - Updates On The Novel Coronavirus Disease 2019 (2019-nCoV) Situation In Malaysia] [press release]. Malaysia: Ministry of Health Malaysia, 30 January 2020.
26. Loheswar R. Putrajaya announces task force to track down, bring back Malaysians in Hubei province. Malay Mail. 2020 30 January 2020.
27. Tan M. Malaysian airport cleaners diligently wipe down surfaces to protect travellers from Wuhan virus. Malay Mail. 2020 30 January 2020.
28. Arumugam T, Yusof TA. 'No plan to bar them from class'. New Straits Times. 2020 30 January 2020.
29. Kenyataan Akhbar KPK 30 Januari 2020 – Situasi Terkini Jangkitan 2019 Novel Coronavirus (2019-nCoV) Di Malaysia [Ministry of Health Malaysia Press Statement on 30 January 2020 - Updates On The Novel Coronavirus Disease 2019 (2019-nCoV) Situation In Malaysia] [press release]. Malaysia: Ministry of Health Malaysia, 30 January 2020.
30. Kenyataan Akhbar YBMK 31 Januari 2020 - Situasi Semasa Jangkitan Penyakit Coronavirus 2019 (COVID-19) di Malaysia [Ministry of Health Malaysia Press Statement on 31 Januari 2020 - Updates On The Coronavirus Disease 2019 (COVID-19) Situation In Malaysia] [press release]. Malaysia: Ministry of Health Malaysia, 31 January 2020.
31. Tan B. Johor official urges Malaysians to unite,fight discrimination on Wuhan coronavirus. Malay Mail. 2020 31 January 2020.
32. Povera A. Malaysia has measures in place to combat coronavirus. New Straits Times. 2020 31 January 2020.

33. Kenyataan Akhbar KPK 1 Februari 2020 – Situasi Terkini Jangkitan 2019 Novel Coronavirus (2019-nCoV) di Malaysia [Ministry of Health Malaysia Press Statement on 1 February 2020 - Updates On The Novel Coronavirus Disease 2019 (2019-nCoV) Situation In Malaysia] [press release]. Malaysia: Ministry of Health Malaysia, 1 February 2020.
34. Kenyataan Akhbar Menteri Kesihatan Malaysia 3 Februari 2020 - Situasi Terkini Jangkitan 2019 Novel Coronavirus (2019-nCoV) di Malaysia [Minister of Health Malaysia Press Statement on 3 February 2020 - Updates On The Novel Coronavirus Disease 2019 (2019-nCoV) Situation In Malaysia] [press release]. Malaysia: Ministry of Health, 3 Februari 2020.
35. Ministry of Health Malaysia Press Statement on 5 February 2020 - Updates On 2019-nCoV Disease Situation In Malaysia [press release]. Malaysia: Ministry of Health Malaysia, 5 February 2020.
36. Kenyataan Akhbar Kementerian Kesihatan Malaysia 6 Februari 2020 - Situasi Terkini Jangkitan 2019-nCoV dan Pengesanan Kes Baharu di Malaysia [Ministry of Health Malaysia Press Statement on 6 February 2020 - Updates On 2019-nCoV Disease Situation and Detection of New Cases In Malaysia] [press release]. Malaysia: Ministry of Health Malaysia, 6 February 2020.
37. Kenyataan Akhbar Kementerian Kesihatan Malaysia 7 Februari 2020 - Situasi Terkini Jangkitan 2019-nCoV dan Pengesanan Kes Baharu di Malaysia [Ministry of Health Malaysia Press Statement on 7 February 2020 - Updates On 2019-nCoV Disease Situation and Detection of New Cases In Malaysia] [press release]. Malaysia: Ministry of Health Malaysia, 7 February 2020.
38. Ministry of Health Malaysia Press Statement on 8 February 2020 - Essentials required in “Wartime” against Novel Coronavirus (2019-nCoV) in public areas [press release]. Malaysia: Ministry of Health Malaysia, 8 February 2020.
39. Kenyataan Akhbar KPK 9 Februari 2020 – Situasi Terkini Jangkitan 2019-NCoV dan Pengesanan Kes Baharu di Malaysia [Ministry of Health Press Statement on 9 February 2020 - Updates On The Coronavirus Disease 2019 (COVID-19) Situation In Malaysia] [press release]. Malaysia: Ministry of Health Malaysia, 9 February 2020.
40. Abas A. Ministry to keep close watch on face mask prices and supply. New Straits Times. 2020 10 February 2020.
41. Kenyataan Akhbar Kementerian Kesihatan Malaysia 10 Februari 2020 - Situasi Terkini Jangkitan 2019-nCoV dan Pengesanan Kes Baharu di Malaysia [Ministry of Health Malaysia Press Statement on 10 February 2020 - Updates On 2019-nCoV Disease Situation and Detection of New Cases In Malaysia] [press release]. Malaysia: Ministry of Health Malaysia, 10 February 2020.
42. Kenyataan Akhbar Kementerian Kesihatan Malaysia 10 Februari 2020 - Situasi Terkini Jangkitan 2019-nCoV di Malaysia [Ministry of Health Malaysia Press Statement on 10 February 2020 - Updates On 2019-nCoV Disease Situation In Malaysia] [press release]. Malaysia: Ministry of Health Malaysia, 10 February 2020.
43. Ministry of Health Malaysia Press Statement on 12 February 2020 - Updates On The Coronavirus Disease 2019 (COVID-19) Situation In Malaysia [press release]. Malaysia: Ministry of Health Malaysia, 12 February 2020.
44. Kenyataan Akhbar KPK 23 Februari 2020 – Pelaksanaan Survelan Penyakit Coronavirus 2019 (Covid-19) Bagi Mengesan Kes dalam Masyarakat Tempatan dan Situasi Terkini Jangkitan di Malaysia [Ministry of Health Malaysia Press Statement on 23 February 2020 - Implementation of Coronavirus Disease Surveillance 2019 (Covid-19) to Detect Cases in Local Communities & Updates On The Coronavirus Disease 2019 (COVID-19) Situation In Malaysia] [press release]. Malaysia: Ministry of Health Malaysia, 23 February 2020.
45. Perimbanayagam K. No need for alarm, Covid-19 in Malaysia yet to reach crisis-level. New Straits Times. 2020 12 February 2020.

46. Kenyataan Akhbar KPK 13 Februari 2020 – Penjelasan Isu Kes Coronavirus Disease (COVID-19) yang didakwa berlaku di Pulau Pinang [Ministry of Health Malaysia Press Statement on 13 February 2020 - Clarification of Coronavirus Disease (COVID-19) Case Issues allegedly occurred in Penang] [press release]. Malaysia: Ministry of Health Malaysia, 13 February 2020.
47. Kenyataan Akhbar KPK 13 Februari 2020 – Situasi Terkini Jangkitan Coronavirus Disease (COVID-19) di Malaysia [Ministry of Health Malaysia Press Statement on 13 February 2020 - Updates On The Coronavirus Disease 2019 (COVID-19) Situation In Malaysia] [press release]. Malaysia: Ministry of Health Malaysia, 13 February 2020.
48. Bernama. Covid-19: Private sector encouraged to install thermal scanners. 2020 14 February 2020.
49. Press Statement Updates on the Coronavirus Disease 2019 (COVID-19) Situation in Malaysia - Deputy Prime Minister of Malaysia Dato' Seri Dr Wan Azizah Dr Wan Ismail Chairperson Central Disaster Management Committee (Jawatankuasa Pengurusan Bencana Pusat JPBP) [press release]. Malaysia: National Disaster Management Agency, 16 February 2020.
50. Kenyataan Akhbar YBMK 18 Februari 2020 - Situasi Semasa Jangkitan Penyakit Coronavirus 2019 (COVID-19) di Malaysia [Ministry of Health Malaysia Press Statement on 18 February 2020 - Updates On The Coronavirus Disease 2019 (COVID-19) Situation In Malaysia] [press release]. Malaysia: Ministry of Health, 18 February 2020.
51. Kenyataan Akhbar YBMK 19 Februari 2020 - Situasi Semasa Jangkitan Penyakit Coronavirus 2019 (COVID-19) di Malaysia [Ministry of Health Malaysia Press Statement on 19 February 2020 - Updates On The Coronavirus Disease 2019 (COVID-19) Situation In Malaysia] [press release]. Malaysia: Ministry of Health, 19 February 2020.
52. Kenyataan Akhbar YBMK 20 Februari 2020 - Situasi Semasa Jangkitan Penyakit Coronavirus 2019 (COVID-19) di Malaysia [Ministry of Health Malaysia Press Statement on 20 February 2020 - Updates On The Coronavirus Disease 2019 (COVID-19) Situation In Malaysia] [press release]. Malaysia: Ministry of Health, 20 February 2020.
53. Press Statement DG of Health 22 February 2020 -Updates on the Coronavirus Disease 2019 (COVID-19) Case 22 in Malaysia [press release]. Malaysia: Ministry of Health Malaysia, 22 February 2020.
54. Kenyataan Akhbar KPK 23 Februari 2020 – Situasi Terkini Jangkitan Coronavirus Disease (COVID-19) di Malaysia - Kapal Persiaran World Dream [Ministry of Health Malaysia Press Statement on 23 February 2020 -Updates On The Coronavirus Disease 2019 (COVID-19) Situation In Malaysia - World Dream Cruise] [press release]. Malaysia: Ministry of Health Malaysia, 23 February 2020.
55. Kenyataan Akhbar KPK 21 April 2020 – Situasi Semasa Jangkitan Penyakit Coronavirus 2019 (COVID-19) di Malaysia [Ministry of Health Malaysia Press Statement on 21 April 2020, Updates On The Coronavirus Disease 2019 (COVID-19) Situation In Malaysia] [press release]. Malaysia: Ministry of Health Malaysia, 21 April 2020.
56. Penggantungan Sementera Kemasukan Jemaah Umrah, Ziarah dan Pelancong ke Arab Saubi [Temporary Suspension of Umrah, Pilgrimage and Tourist Entry to Saudi Arabia] [press release]. Malaysia: Ministry of Foreign Affairs Malaysia, 27 February 2020.
57. Ministry of Health Malaysia. Guidelines COVID-19 Management - Annex 1 : Case Definition of COVID-19 Malaysia: Ministry of Health Malaysia; 2020 [updated 26 February 2020; cited 7 June 2020]. Available from: http://www.moh.gov.my/moh/resources/Penerbitan/Garis%20Panduan/Pengurusan%20KEsihatan%20&%20kawalan%20pykit/2019-nCOV/Bil%204%20%202020/Annex%201%20case%20definition%20COVID%2026022020.pdf.

58. Ministry of Health Malaysia. Guidelines COVID-19 Management Annex 2a: Management of PUI not admitted Malaysia: Ministry of Health Malaysia; 2020 [updated 26 February 2020; cited 7 June 2020]. 4]. Available from: https://www.moh.gov.my/moh/resources/Penerbitan/Garis%20Panduan/Pengurusan%20KEsihatan%20&%20kawalan%20pykit/2019-nCOV/Bil%204%20%202020/Annex%202a%20PUI%20not%20admitted_26022020.pdf.
59. Ministry of Health Malaysia. Guidelines COVID-19 Management Annex 2d : Work Process in Management of Suspected COVID-19 Patients in Emergency and Trauma Department Malaysia: Ministry of Health Malaysia; 2020 [updated 26 February 2020. Available from: https://www.moh.gov.my/moh/resources/Penerbitan/Garis%20Panduan/Pengurusan%20KEsihatan%20&%20kawalan%20pykit/2019-nCOV/Bil%204%20%202020/Annex%202d%20Work%20Process%20of%20pre%20hospital%20care%20and%20emergency%20and%20trauma%20department_26022020.pdf.
60. Joint Statement MOH Malaysia-MOH Singapore 26 February 2020 – The First Meeting of the Malaysia – Singapore Joint Working Group (JWG) for the Coronavirus Disease (COVID-19) [press release]. Malaysia: Ministry of Health Malaysia, 26 February 2020.
61. Ministry of Health Malaysia. Guidelines COVID-19 Management Annex 3 : Senarai Designated Hospital Bagi Mengendalikan Kes COVID-19 [Guidelines COVID-19 Management Annex 3 : List of Designated Hospitals to Handle COVID-19 Cases] Malaysia: Ministry of Health; 2020 [updated 26 February 2020. Available from: http://www.moh.gov.my/moh/resources/Penerbitan/Garis%20Panduan/COVID19/Annex_3_Screening_centre_24032020.pdf.
62. Bernama. Special aircraft on second Wuhan evacuation mission arrives in KLIA. The Star. 2020 26 February 2020.
63. Kenyataan Akhbar KPK 27 Februari 2020 – Situasi Terkini Jangkitan Coronavirus Disease (COVID-19) di Malaysia [Ministry of Health Malaysia Press Statement on 27 February 2020 - Updates On The Coronavirus Disease 2019 (COVID-19) Situation In Malaysia] [press release]. Malaysia: Ministry of Health Malaysia, 27 February 2020.
64. Kenyataan Akhbar KPK 28 Februari 2020 – Situasi Terkini Jangkitan Coronavirus Disease (COVID-19) di Malaysia [Ministry of Health Malaysia Press Statement on 28 February 2020 - Updates On The Coronavirus Disease 2019 (COVID-19) Situation In Malaysia] [press release]. Malaysia: Ministry of Health Malaysia, 28 February 2020.
65. Kenyataan Akhbar KPK 29 Februari 2020 – Situasi Semasa Jangkitan Penyakit Coronavirus 2019 (COVID-19) di Malaysia [Ministry of Health Malaysia Press Statement on 29 February 2020 - Updates On The Coronavirus Disease 2019 (COVID-19) Situation In Malaysia] [press release]. Malaysia: Ministry of Health, 29 February 2020.
66. Kenyataan Akhbar KPK 1 Mac 2020 – Situasi Semasa Jangkitan Penyakit Coronavirus 2019 (COVID-19) di Malaysia [Ministry of Health Malaysia Press Statement on 1 March 2020 - Updates On The Coronavirus Disease 2019 (COVID-19) Situation In Malaysia] [press release]. Malaysia: Ministry of Health Malaysia, 1 March 2020.
67. DoctorOnCall. COVID-19 Testing Services in Malaysia: DoctorOnCall; 2020 [Available from: https://www.doctoroncall.com.my/medicine/coronavirus-covid-19-test-kit.
68. Kenyataan Media Pejabat Perdana Menteri [Prime Minister's Office Press Statement] [press release]. Malaysia: Prime Minister's Office of Malaysia, 10 March 2020.

69. Kenyataan Akhbar KPK 4 Mac 2020 – Situasi Semasa Jangkitan Penyakit Coronavirus 2019 (COVID-19) di Malaysia [Ministry of Health Malaysia Press Statement on 4 March 2020 - Updates On The Coronavirus Disease 2019 (COVID-19) Situation In Malaysia] [press release]. Malaysia: Ministry of Health Malaysia, 4 March 2020.
70. Kenyataan Akhbar KPK 5 Mac 2020 – Situasi Semasa Jangkitan Penyakit Coronavirus 2019 (COVID-19) di Malaysia & Mesyuarat Jawatankuasa Kerja Kluster Penyakit Coronavirus (COVID-19) Bil. 2 Tahun 2020 [Ministry of Health Malaysia Press Statement on 15 March 2020, Updates On The Coronavirus Disease 2019 (COVID-19) Situation In Malaysia & Technical Working Committee for COVID-19 Cluster Meeting No. 2 Year 2020] [press release]. Malaysia: Ministry of Health Malaysia, 5 March 2020.
71. Kenyataan Akhbar KPK 6 Mac 2020 – Situasi Semasa Jangkitan Penyakit Coronavirus 2019 (COVID-19) di Malaysia [Ministry of Health Malaysia Press Statement on 6 March 2020 - Updates On The Coronavirus Disease 2019 (COVID-19) Situation In Malaysia] [press release]. Malaysia: Ministry of Health Malaysia, 6 March 2020.
72. Kenyataan Akhbar Kementerian Kesihatan Malaysia Situasi Semasa Jangkitan Penyakit Coronavirus 2019 (Covid-19) Di Malaysia 7 Mac 2020 [Ministry of Health Malaysia Press Statement on 7 March 2020 - Updates On The Coronavirus Disease 2019 (COVID-19) Situation In Malaysia] [press release]. Malaysia: Ministry of Health, 7 March 2020.
73. Kenyataan Akhbar KPK 8 Mac 2020 – Situasi Semasa Jangkitan Penyakit Coronavirus 2019 (COVID-19) di Malaysia [Ministry of Health Malaysia Press Statement on 8 March 2020 - Updates On The Coronavirus Disease 2019 (COVID-19) Situation In Malaysia] [press release]. Malaysia: Minister of Health Malaysia, 8 March 2020.
74. Kenyataan Akhbar KPK 9 Mac 2020 – Situasi Semasa Jangkitan Penyakit Coronavirus 2019 (COVID-19) di Malaysia [Ministry of Health Malaysia Press Statement on 9 March 2020 - Updates On The Coronavirus Disease 2019 (COVID-19) Situation In Malaysia] [press release]. Malaysia: Ministry of Health Malaysia, 9 March.
75. Ministry of Health Malaysia. Guidelines COVID-19 Management Annex 25: Management Guidelines For Workplaces. Malaysia: Ministry of Health; 2020.
76. Kenyataan Akhbar KPK 11 Mac 2020 – Situasi Semasa Jangkitan Penyakit Coronavirus 2019 (COVID-19) di Malaysia [Ministry of Health Malaysia Press Statement on 11 March 2020 - Updates On The Coronavirus Disease 2019 (COVID-19) Situation In Malaysia] [press release]. Malaysia: Ministry of Health Malaysia, 11 March 2020.
77. Ministry of Health Malaysia. Guidelines COVID-19 Management Annex 26: Covid-19: Social Distancing Guidelines For Workplace, Homes And Individuals. Malaysia: Ministry of Health; 2020.
78. Kenyataan Akhbar KPK 12 Mac 2020 – Situasi Semasa Jangkitan Penyakit Coronavirus 2019 (COVID-19) di Malaysia [Ministry of Health Malaysia Press Statement on 12 March 2020, Updates On The Coronavirus Disease 2019 (COVID-19) Situation In Malaysia] [press release]. Malaysia: Ministry of Health, 12 March 2020.
79. KPKT Umum Inisiatif Pengecualian Sewa Satu Bulan Rumah Projek Perumahan Rakyat(PPR) [KPKT Announces One Month Rental Exemption Initiative for Household under People Home Project (PPR)] [press release]. Malaysia: Ministry of Housing and Local Government, 21 March 2020.
80. Zainul E. Malaysia has yet to achieve target of conducting 16,500 Covid-19 tests per day. The Edge Markets. 2020 14 April 2020.

81. Kenyataan Akhbar KPK 13 Mac 2020 – Situasi Semasa Jangkitan Penyakit Coronavirus 2019 (COVID-19) di Malaysia [Ministry of Health Malaysia Press Statement on 13 March 2020 - Updates On The Coronavirus Disease 2019 (COVID-19) Situation In Malaysia] [press release]. Malaysia: Ministry of Health, 13/03/2020.
82. Bernama. Govt launches Covid-19 fund to help those affected by outbreak. 2020 11 March 2020.
83. Kaos JJ. M'sians in Covid-19-affected countries told to register at nearest embassy. 2020 13 March 2020.
84. Kenyataan Akhbar KPK 14 Mac 2020 -Situasi Semasa Jangkitan Penyakit Coronavirus 2019 (COVID-19) di Malaysia [Ministry of Health Malaysia Press Statement on 14 March 2020 - Updates On The Coronavirus Disease 2019 (COVID-19) Situation In Malaysia] [press release]. Malaysia: Ministry of Health Malaysia, 14 March 2020.
85. Hammim R. Enhanced MCO issued for two areas in Simpang Renggam. New Strait Times. 2020 14 March 2020.
86. Kenyataan Akhbar YBMK 15 Mac 2020 - Situasi Semasa Jangkitan Penyakit Coronavirus 2019 (COVID-19) di Malaysia [Ministry of Health Malaysia Press Statement on 15 March 2020 - Updates On The Coronavirus Disease 2019 (COVID-19) Situation In Malaysia] [press release]. Malaysia: Ministry of Health, 15 March 2020.
87. Kenyataan Akhbar KPK 15 Mac 2020 – Situasi Semasa Jangkitan Penyakit Coronavirus 2019 (COVID-19) di Malaysia [Ministry of Health Malaysia Press Statement on 15 March 2020, Updates On The Coronavirus Disease 2019 (COVID-19) Situation In Malaysia] [press release]. Malaysia: Ministry of Health, 15 March 2020.
88. Prevention and Control of Infectious Disease Act 1988 Declaration of Quarantine Station 2020, 342 (2020).
89. Prevention and Control of Infectious Disease Act 1988 Declaration of Quarantine Station (No. 2) 2020, 342 (2020).
90. Prevention and Control of Infectious Disease Act 1988 Declaration of Quarantine Station (No. 3) 2020, 342 (2020).
91. Prevention and Control of Infectious Disease Act 1988 Declaration of Quarantine Station (No. 6) 2020, 342 (2020).
92. Prevention and Control of Infectious Disease Act 1988 Declaration of Quarantine Station (No. 7) 2020, 342 (2020).
93. Prevention and Control of Infectious Disease Act 1988 Declaration of Quarantine Station (No. 8) 2020, 342 (2020).
94. Prevention and Control of Infectious Disease Act 1988 Declaration of Quarantine Station (No. 9) 2020, 342 (2020).
95. Prevention and Control of Infectious Disease Act 1988 Declaration of Quarantine Station (No. 10) 2020, 342 (2020).
96. Prevention and Control of Infectious Disease Act 1988 Declaration of Quarantine Station (No. 5) 2020, 342 (2020).
97. Prevention and Control of Infectious Disease Act 1988 Declaration of Quarantine Station (No. 11) 2020, 342 (2020).
98. Prevention and Control of Infectious Disease Act 1988 Declaration of Quarantine Station (No. 4) 2020, 342 (2020).
99. Kenyataan Akhbar KPK 16 Mac 2020 – Situasi Semasa Jangkitan Penyakit Coronavirus 2019 (COVID-19) di Malaysia [Ministry of Health Malaysia Press Statement on 16 March 2020 - Updates On The Coronavirus Disease 2019 (COVID-19) Situation In Malaysia] [press release]. Malaysia: Ministry of Health, 16 March 2020.
100. Prime Minister’s Office of Malaysia. The Prime Minister’s Special Message on COVID-19 16 March 2020 Malaysia: Prime Minister’s Office of Malaysia; 2020 [updated 16 March 2020; cited 19 March 2020]. Available from: https://www.pmo.gov.my/2020/03/perutusan-khas-yab-perdana-menteri-mengenai-covid-19-16-mac-2020/.
101. Aziz A. PM to address nation tonight on Covid-19 after National Security Council meeting. The Edge Markets. 2020 16 March 2020.
102. Prevention and Control of Infectious Diseases (Declaration of Infected Local Areas) Order 2020, 342 (2020).

103. Prevention and Control of Infectious Diseases (Measures within Infected Local Areas) Regulations 2020, 342 (2020).
104. Kenyataan Akhbar KPK 17 Mac 2020 – Situasi Semasa Jangkitan Penyakit Coronavirus 2019 (COVID-19) di Malaysia [Ministry of Health Malaysia Press Statement on 17 March 2020 - Updates On The Coronavirus Disease 2019 (COVID-19) Situation In Malaysia] [press release]. Malaysia: Ministry of Health, 17 March 2020.
105. National Security Council Malaysia. Soalan Lazim (FAQ's) – Bil. 1 (Dikemaskini) Perintah Kawalan Pergerakan (Movement Control Order) 18 – 31 Mac 2020 [Frequently Asked Questions (FAQ's - No.1 (Updated) Movement Control Order (Movement Control Order) 18 - 31 March 2020] Malaysia: National Security Council Malaysia; 2020 [updated 17 March 2020; cited 17 August 2020]. Available from: https://www.mkn.gov.my/web/wp-content/uploads/sites/3/2020/03/FAQ-Bil1730.pdf.
106. Kenyataan Akhbar KPK 25 June 2020 – Situasi Semasa Jangkitan Penyakit Coronavirus 2019 (COVID-19) di Malaysia [Ministry of Health Malaysia Press Statement on 25 Jun 2020, Updates On The Coronavirus Disease 2019 (COVID-19) Situation In Malaysia] [press release]. Malaysia: Ministry of Health, 25 June 2020.
107. National Registration Department of Malaysia. Frequently Asked Questions Regarding The Operation Of National Registration Department During the Period of Movement Control Order. Malaysia: National Registration Department of Malaysia; 2020.
108. Minister of Health Malaysia. Secretary-General Circular Letter No. 2 of 2020 Payment of Special Allowances to Doctors and Health Professionals in Dealing with and Controlling the Spread of the Covid-19 Outbreak Malaysia: Ministry of Health; 2020 [updated 18 March 2020; cited 20 June 2020]. Available from: https://www.moh.gov.my/index.php/database_stores/store_view_page/10/379.
109. National Disaster Management Agency. Garis Panduan Pemberian Bantuan Khas COVID-19 [COVID-19 Special Assistance Guidelines] Malaysia: National Disaster Management Agency; 2020 [updated 18 March 2020; cited 19 July 2020]. Available from: http://www.nadma.gov.my/images/nadma/documents/bantuan/Bantuan_Khas_COVID19_garispanduan.pdf.
110. National Security Council Malaysia. Kenyataan Media Majlis Keselamatan Negara, Jabatan Perdana Menteri 18 Mac 2020 [National Security Council, Prime Minister Department Press Statement 18 March 2020] Malaysia: National Security Council Malaysia,; 2020 [updated 18 March 2020; cited 8 September 2020]. Available from: https://www.mkn.gov.my/web/wp-content/uploads/sites/3/2020/03/PERGERAKAN-KAWALAN-COVID19-amended-18032020-UPDATED-12.12AM.pdf.
111. Kenyataan Akhbar YBMK 18 Mac 2020 - Situasi Semasa Jangkitan Penyakit Coronavirus 2019 (COVID-19) di Malaysia [Ministry of Health Malaysia Press Statement on 18 March 2020 - Updates On The Coronavirus Disease 2019 (COVID-19) Situation In Malaysia] [press release]. Malaysia: Ministry of Health Malaysia, 18 March 2020.
112. Ministry of Women FaCD. Kenyataan Media Menteri Pembangunan Wanita, Keluarga Dan Masyarakat [Minister of Women, Family and Community Development Press Statement] Malaysia: Ministry of Women, Family and Community Development; 2020 [updated 18 March 2020; cited 3 August 2020]. Available from: https://www.kpwkm.gov.my/kpwkm/uploads/files/KenyataanMedia/2020/FINAL%20KENYATAAN%20MEDIA%20TALIAN%20KASIH%20BEROPERASI%20SEMULA.pdf.
113. National Security Council Malaysia. Kenyataan Media Majlis Keselamatan Negara Jabatan Perdana Menteri 18 Mac 2020 [National Security Council Prime Minister Department Press Statement 18 March 2020] Malaysia: National Security Council Malaysia; 2020 [updated 18

March 2020; cited 5 August 2020]. Available from: https://www.mkn.gov.my/web/wp-content/uploads/sites/3/2020/03/2.-KENYATAAN-MEDIA-MKN-PERGERAKAN-KAWALAN-COVID19-MITIfinal.pdf.
114. Kenyataan Akhbar KPK 20 Mac 2020 – Situasi Semasa Jangkitan Penyakit Coronavirus 2019 (COVID-19) di Malaysia [Ministry of Health Malaysia Press Statement on 20 March 2020 - Updates On The Coronavirus Disease 2019 (COVID-19) Situation In Malaysia] [press release]. Malaysia: Ministry of Health Malaysia, 20 March 2020.
115. Palansamy Y. Senior minister: Military to help enforce Covid-19 shutdown from Sunday. Malay Mail. 2020 20 March 2020.
116. Kenyataan Akhbar KPK 21 Mac 2020 – Situasi Semasa Jangkitan Penyakit Coronavirus 2019 (COVID-19) di Malaysia [Ministry of Health Malaysia Press Statement on 21 March 2020 - Updates On The Coronavirus Disease 2019 (COVID-19) Situation In Malaysia] [press release]. Malaysia: Ministry of Health, 21 March 2020.
117. Daim N. 500 more ventilators needed in preparation for increasing Covid-19 cases. New Straits Times. 2020 21 March 2020.
118. Yusof TA. 212 Malaysians arrive safely home from Tashkent. New Straits Times. 2020 22 March 2020.
119. Minister of Health Malaysia. Minister of Health Malaysia Youtube Videos Malaysia: Ministry of Health; 2020 [updated 2 November 2020. Available from: https://www.youtube.com/c/KementerianKesihatanMalaysia/videos.
120. Minister of Health Malaysia. Guidelines COVID-19 Management Annex 22: Guideline On Management Of Coronavirus Disease 2019 (COVID-19) in Surgery Malaysia: Ministry of Health; 2020 [updated 22 March 2020. Available from: https://www.moh.gov.my/moh/resources/Penerbitan/Garis%20Panduan/COVID19/annex_22_COVID-19_Guidelines_Surgical_22032020.pdf.
121. Minister of Health Malaysia. Guidelines COVID-19 Management Annex 23: Guideline On Management Of Coronavirus Disease 2019 (COVID-19) in Pregnancy Malaysia: Ministry of Health; 2020 [updated 22 March 2020. Available from: https://www.moh.gov.my/moh/resources/Penerbitan/Garis%20Panduan/COVID19/Annex_23_Guidelines_COVID_Pregnancy_22032020.pdf.
122. Minister of Health Malaysia. Guidelines COVID-19 Management Annex 28: Guideline On Management Of Coronavirus Disease 2019 (COVID-19) in Dialysis Centres & Nephrology Units Malaysia: Ministry of Health; 2020 [updated 22 March 2020. Available from: https://www.moh.gov.my/moh/resources/Penerbitan/Garis%20Panduan/COVID19/Annex_28_Dialysis_and_Nephrology_Units_22032020.pdf.
123. Minister of Health Malaysia. Guidelines COVID-19 Management Annex 2f: Flow Chart for Home Sampling of Covid-19 Malaysia: Ministry of Health; 2020 [updated 22 March 2020. Available from: http://www.moh.gov.my/moh/resources/Penerbitan/Garis%20Panduan/COVID19/Annex_2f_uberisation_22032020.pdf.
124. Minister of Health Malaysia. Guidelines COVID-19 Management Annex 30: Otorhinolaryngology Services, Ministry of Health Malaysia. Malaysia: Ministry of Health; 2020.
125. Minister of Health Malaysia. Guidelines COVID-19 Management Annex 7: Notification Form. Malaysia: Ministry of Health; 2020.
126. Minister of Health Malaysia. Guidelines COVID-19 Management Annex 9 : Management Of COVID-19 At Point Of Entry. Malaysia: Ministry of Health; 2020.
127. Ministry of Health Malaysia. Guidelines COVID-19 Appendix 1: Health Declaration Form Malaysia: Ministry of Health; 2020 [updated 22 March 2020. Available from: https://www.moh.gov.my/moh/resources/Penerbitan/Garis%20Panduan/COVID19/Appendix_1_Health_Declaration_Form_COVID-19_MCO.pdf.

128. Ministry of Health Malaysia. Guidelines COVID-19 Management Annex 2 : Passenger Locator Form (as per WHO/ICAO). Malaysia: Ministry of Health; 2020.
129. Kenyataan Akhbar KPK 22 Mac 2020 – Situasi Semasa Jangkitan Penyakit Coronavirus 2019 (COVID-19) di Malaysia [Ministry of Health Malaysia Press Statement on 22 March 2020 - Updates On The Coronavirus Disease 2019 (COVID-19) Situation In Malaysia] [press release]. Malaysia: Ministry of Health, 22 March 2020.
130. Ministry of Health Malaysia. Guidelines COVID-19 Management Annex 32 : Quarantine centre. Malaysia: Ministry of Health; 2020.
131. Bernama. Rina Harun: 55 individuals returning to Malaysia from Iran on Sunday (March 22). 2020 21 March 2020.
132. Kenyataan Akhbar KPK 23 Mac 2020 – Situasi Semasa Jangkitan Penyakit Coronavirus 2019 (COVID-19) di Malaysia [Ministry of Health Malaysia Press Statement on 23 March 2020 - Updates On The Coronavirus Disease 2019 (COVID-19) Situation In Malaysia] [press release]. Malaysia: Ministry of Health, 23 March 2020.
133. Povera A. 4 locations in KL identified as Covid-19 red zones. New Straits Times. 2020 22 March 2020.
134. Povera A, Yunus A. PM: Additional measures to lessen people's burden, enhance healthcare capabilities. New Straits Times. 2020 23 March 2020.
135. Ministry of Health Malaysia. Guideline COVID-19 Management No.5/2020 Malaysia: Ministry of Health; 2020 [updated 27 October 2020. Available from: http://covid-19.moh.gov.my/garis-panduan/garis-panduan-kkm.
136. Minister of Health Malaysia. Guidelines COVID-19 Management Annex 27 COVID-19: Management Guidelines For Special Settings: Minister of Health Malaysia; 2020 [updated 25 March 2020; cited 7 October 2020]. Available from: http://www.moh.gov.my/moh/resources/Penerbitan/Garis%20Panduan/COVID19/Annex_27_COVID_guide_for_special_settings_25032020_.pdf.
137. Kenyataan Akhbar KPK 24 Mac 2020 – Situasi Semasa Jangkitan Penyakit Coronavirus 2019 (COVID-19) di Malaysia [Ministry of Health Malaysia Press Statement on 24 March 2020 - Updates On The Coronavirus Disease 2019 (COVID-19) Situation In Malaysia] [press release]. Malaysia: Ministry of Health, 24 March 2020.
138. Reuters. Covid-19: Health Ministry to increase testing to 16,500 daily. New Straits Times. 2020 24 April 2020.
139. Bernama. MOH identifies 3,000 contract staff to help combat Covid-19. theSundaily. 2020 24 March 2020.
140. Prevention and Control of Infectious Diseases (Declaration of Infected Local Areas) (Extension of Operation) Order 2020, 342 (2020).
141. Kenyataan Akhbar KPK 26 Mac 2020 – Situasi Semasa Jangkitan Penyakit Coronavirus 2019 (COVID-19) di Malaysia [Ministry of Health Malaysia Press Statement on 26 March 2020 - Updates On The Coronavirus Disease 2019 (COVID-19) Situation In Malaysia] [press release]. Malaysia: Ministry of Health, 26 March 2020.
142. Sarawak Multimedia Authority. Sarawak Readies Digital Surveillance Solution To Fight COVID-19 Malaysia: Sarawak Multimedia Authority; 2020 [updated 27 March 2020; cited 5 August 2020]. Available from: https://www.sma.gov.my/modules/web/pages.php?mod=news&sub=news_view&menu_id=&sub_id=&nid=43&m=3&y=2020.
143. Kenyataan Akhbar KPK 27 Mac 2020 – Situasi Semasa Jangkitan Penyakit Coronavirus 2019 (COVID-19) di Malaysia [Ministry of Health Malaysia Press Statement on 27 March 2020 - Updates On The Coronavirus Disease 2019 (COVID-19) Situation In Malaysia] [press release]. Malaysia: Ministry of Health Malaysia, 27 March 2020.

144. Prime Minister's Office of Malaysia. Prihatin Rakyat Economic Stimulus Package (PRIHATIN) Speech Text Malaysia: Prime Minister's Office; 2020 [updated 27 March 2020. Available from: https://www.pmo.gov.my/2020/03/speech-text-prihatin-esp/.
145. Kenyataan Akhbar KPK 28 Mac 2020 – Situasi Semasa Jangkitan Penyakit Coronavirus 2019 (COVID-19) di Malaysia [Ministry of Health Malaysia Press Statement on 28 March 2020 - Updates On The Coronavirus Disease 2019 (COVID-19) Situation In Malaysia] [press release]. Malaysia: Ministry of Health, 28 March 2020.
146. Bernama. China's donated test kits and face masks arrive in Malaysia. Free Malaysia Today. 2020 28 March 2020.
147. Kenyataan Akhbar KPK 29 March 2020 -Situasi Semasa Jangkitan Penyakit Coronavirus 2019 (COVID-19) di Malaysia [Ministry of Health Malaysia Press Statement on 29 March 2020 - Updates On The Coronavirus Disease 2019 (COVID-19) Situation In Malaysia] [press release]. Malaysia: Ministry of Health, 29 March 2020.
148. Kenyataan Akhbar Menteri Kanan Mengenai Perintah Kawalan Pergerakan Diperketatkan (PKPD) di Batu 21-24 Sungai Lui, Hulu Langat, Selangor [Press Statement] [press release]. Prime Minister's Office of Malaysia, 29 March 2020.
149. Kenyataan Akhbar KPK 30 March 2020 -Situasi Semasa Jangkitan Penyakit Coronavirus 2019 (COVID-19) di Malaysia [Ministry of Health Malaysia Press Statement on 30 March 2020 - Updates On The Coronavirus Disease 2019 (COVID-19) Situation In Malaysia] [press release]. Malaysia: Ministry of Health, 30 March 2020.
150. Prime Minister's Office of Malaysia. COVID-19: PM visits MAEPS Low-Risk Patient Quarantine & Treatment Centre Malaysia: Prime Minister's Office; 2020 [updated 30 March 2020; cited 23 August 2020]. Available from: https://www.pmo.gov.my/2020/03/covid-19-pm-visits-maeps-low-risk-patient-quarantine-treatment-centre/.
151. The Malaysian Insight. Hishammuddin thanks UAE for medical equipment donation. The Malaysian Insight. 2020 30 March 2020.
152. Mohsen AS. More than 4,300 M'sians still stranded overseas, says deputy minister. theSundaily. 2020 31 March 2020.
153. Coalition launched to accelerate research on the prevention and treatment of COVID-19 in low- and middle-income countries [press release]. Malaysia: Ministry of Health, 31 March 2020.
154. Prevention and Control of Infectious Diseases (Measures within Infected Local Areas) (No. 2) Regulations 2020, 342 (2020).
155. Kenyataan Akhbar KPK 1 April 2020 -Situasi Semasa Jangkitan Penyakit Coronavirus 2019 (COVID-19) di Malaysia [Ministry of Health Malaysia Press Statement on 1 April 2020 - Updates On The Coronavirus Disease 2019 (COVID-19) Situation In Malaysia] [press release]. Malaysia: Ministry of Health, 01 April 2020.
156. Bernama. COVID-19: Singapore contributes 5,000 flocked swabs to Malaysia. Bernama. 2020 1 April 2020.
157. Hariz Mohd. Malaysia sasar sedia 1,400 ventilator untuk rawat pesakit Covid-19. Malaysiakini. 2020 1 April 2020.
158. Kenyataan Akhbar KPK 2 April 2020 – Situasi Semasa Jangkitan Penyakit Coronavirus 2019 (COVID-19) di Malaysia [Ministry of Health Malaysia Press Statement on 2 April 2020 - Updates On The Coronavirus Disease 2019 (COVID-19) Situation In Malaysia] [press release]. Malaysia: Ministry of Health, 02 April 2020.
159. Kenyataan Akhbar KPK 3 April 2020 – Situasi Semasa Jangkitan Penyakit Coronavirus 2019 (COVID-19) di Malaysia [Ministry of Health Malaysia Press Statement on 3 April 2020 - Updates On The Coronavirus Disease 2019 (COVID-19) Situation In Malaysia] [press release]. Malaysia: Ministry of Health, 3 April 2020.
160. Nik Anis M. Ismail Sabri: Straight to quarantine centre for any Malaysians returning home, no exceptions. The Star. 2020 31 March 2020.

161. Kenyataan Akhbar KPK 4 April 2020 -Situasi Semasa Jangkitan Penyakit Coronavirus 2019 (COVID-19) di Malaysia [Ministry of Health Malaysia Press Statement on 4 April 2020 - Updates On The Coronavirus Disease 2019 (COVID-19) Situation In Malaysia] [press release]. Malaysia: Ministry of Health, 4 April 2020.
162. Bernama. Covid-19: Maeps transformed into massive makeshift hospital. Malaysiakini. 2020 7 April 2020.
163. Khairulrijal R. Covid-19: Maeps transformed into massive makeshift hospital. New Straits Times. 2020 4 April 2020.
164. Kenyataan Akhbar KPK 5 April 2020 – Situasi Semasa Jangkitan Penyakit Coronavirus 2019 (COVID-19) di Malaysia [Ministry of Health Malaysia Press Statement on 5 April 2020 - Updates On The Coronavirus Disease 2019 (COVID-19) Situation In Malaysia] [press release]. Malaysia: Ministry of Health, 5 April 2020.
165. Joint Press Release MOH-WHO 6 April 2020 – Malaysia starts global "Solidarity Trial" – a research effort to test possible treatments for COVID-19 [press release]. Malaysia: Ministry of Health, 06/04/2020.
166. Bernama. MOH offering free medicine by mail service till June. Malay Mail,. 2020 7 April 2020.
167. Khairulrijal R. Only National Security Council can issue directives during MCO. New Straits Times. 2020 6 April 2020.
168. Prime Minister's Office of Malaysia. PM receives RM4 mln for COVID-19 Fund, bringing total to RM22.6 mln Malaysia: Prime Minister's Office; 2020 [updated 7 April 2020; cited 23 August 2020]. Available from: https://www.pmo.gov.my/2020/04/pm-receives-rm4-mln-for-covid-19-fund-bringing-total-to-rm22-6-mln/.
169. Kenyataan Akhbar KPK 8 April 2020 – Situasi Semasa Jangkitan Penyakit Coronavirus 2019 (COVID-19) di Malaysia [Ministry of Health Malaysia Press Statement on 8 April 2020 - Updates On The Coronavirus Disease 2019 (COVID-19) Situation In Malaysia] [press release]. Malaysia: Ministry of Health, 8 April 2020.
170. United Nations High Commissioner for Refugees. UNHCR-GoM joint action to prevent, manage Covid-19 infections among refugees: United Nations High Commissioner for Refugees; 2020 [updated 13/04/2020; cited 2 June 2020]. Available from: https://www.unhcr.org/en-my/news/stories/2020/4/5e94189d4/unhcr-gom-joint-action-to-prevent-manage-covid-19-infections-among-refugees.html.
171. Ministry of Health Malaysia. Surat Edaran TKSU (Pengurusan) - Pemakluman Mengenai Dana MOH Fwd My Heroes Oleh Fwd Takaful Berhad [Deputy Secretary General (Management) Circular - Notification About MOH Fwd My Heroes Fund By Fwd Takaful Berhad] Malaysia: Ministry of Health Malaysia; 2020 [updated 9 April 2020. Available from: https://www.moh.gov.my/index.php/database_stores/store_view_page/10/381.
172. Kenyataan Akhbar KPK 9 April 2020 – Situasi Semasa Jangkitan Penyakit Coronavirus 2019 (COVID-19) di Malaysia [Ministry of Health Malaysia Press Statement on 9 April 2020 - Updates On The Coronavirus Disease 2019 (COVID-19) Situation In Malaysia] [press release]. Malaysia: Ministry of Health, 09/04/2020.
173. Kenyataan Akhbar KPK 10 April 2020 – Situasi Semasa Jangkitan Penyakit Coronavirus 2019 (COVID-19) di Malaysia [Ministry of Health Malaysia Press Statement on 10 April 2020, Updates On The Coronavirus Disease 2019 (COVID-19) Situation In Malaysia] [press release]. Malaysia: Ministry of Health, 10 April 2020.
174. Soo WJ. Health D-G: Malaysia likely hit our Covid-19 peak last week, need six weeks to break chain. Malay Mail,. 2020 10 April 2020.
175. Bernama. MAEPS quarantine centre to receive Covid-19 patients next week. Malay Mail,. 2020 10 April 2020.

176. Kenyataan Akhbar KPK 11 April 2020 – Situasi Semasa Jangkitan Penyakit Coronavirus 2019 (COVID-19) di Malaysia [Ministry of Health Malaysia Press Statement on 11 April 2020 - Updates On The Coronavirus Disease 2019 (COVID-19) Situation In Malaysia] [press release]. Malaysia: Ministry of Health, 11 April 2020.
177. Kenyataan Akhbar KPK 12 April 2020 -Situasi Semasa Jangkitan Penyakit Coronavirus 2019 (COVID-19) di Malaysia [Ministry of Health Malaysia Press Statement on 12 April 2020 - Updates On The Coronavirus Disease 2019 (COVID-19) Situation In Malaysia] [press release]. Malaysia: Ministry of Health, 12 April 2020.
178. The Star. Final shipment of ICU beds arrive in Malaysia, all 100 now in local hospitals. 2020 12 April 2020.
179. Kenyataan Akhbar KPK 14 April 2020 -Situasi Semasa Jangkitan Penyakit Coronavirus 2019 (COVID-19) di Malaysia [Ministry of Health Malaysia Press Statement on 14 April 2020 - Updates On The Coronavirus Disease 2019 (COVID-19) Situation In Malaysia] [press release]. Malaysia: Ministry of Health, 14 April 2020.
180. Kenyataan Akhbar KPK 15 April 2020 – Situasi Semasa Jangkitan Penyakit Coronavirus 2019 (COVID-19) di Malaysia [Ministry of Health Malaysia Press Statement on 15 April 2020 - Updates On The Coronavirus Disease 2019 (COVID-19) Situation In Malaysia] [press release]. Malaysia: Ministry of Health, 15 April 2020.
181. Kenyataan Akhbar 16 April 2020 – Situasi Semasa Jangkitan Penyakit Coronavirus 2019 (COVID-19) di Malaysia [Ministry of Health Malaysia Press Statement on 16 April 2020, Updates On The Coronavirus Disease 2019 (COVID-19) Situation In Malaysia] [press release]. Malaysia: Ministry of Health, 16 April 2020.
182. National Disaster Management Agency. Soalan Lazim FAQ - Mengenai Applikasi MySejahtera MKN 17/04/2020 [National Disaster Management Agency, Frequently Asked Questions Regarding MySejahtera NSC Application 17/04/2020] Malaysia: National Disaster Management Agency; 2020 [updated 17 April 2020; cited 19 June 2020]. Available from: http://portalbencana.nadma.gov.my/ms/component/content/article/48-covid-19/faq/1129-faq-mengenai-applikasi-mysejahtera-mkn-17-04-2020?Itemid=101.
183. Kenyataan Akhbar KPK 17 April 2020 – Situasi Semasa Jangkitan Penyakit Coronavirus 2019 (COVID-19) di Malaysia [Ministry of Health Malaysia Press Statement on 17 April 2020 - Updates On The Coronavirus Disease 2019 (COVID-19) Situation In Malaysia] [press release]. Malaysia: Ministry of Health, 17 April 2020.
184. Prime Minister’s Office of Malaysia. King makes personal donation to COVID-19 fund Malaysia: Prime Minister’s Office; 2020 [updated 16 April 2020; cited 23 August 2020]. Available from: https://www.pmo.gov.my/2020/04/king-makes-personal-donation-to-covid-19-fund/.
185. Tariq Q. MCMC launches contact tracing app: Gerak Malaysia (update: not approved by Mosti). 2020 17 April 2020.
186. Kenyataan Akhbar KPK 19 April 2020 – Situasi Semasa Jangkitan Penyakit Coronavirus 2019 (COVID-19) di Malaysia [Ministry of Health Malaysia Press Statement on 19 April 2020 - Updates On The Coronavirus Disease 2019 (COVID-19) Situation In Malaysia] [press release]. Malaysia: Ministry of Health, 19 April 2020.
187. Tan T. Three ministries team up to secure vaccines. 2020 22 April 2020.
188. Kenyataan Akhbar KPK 22 April 2020 – Situasi Semasa Jangkitan Penyakit Coronavirus 2019 (COVID-19) di Malaysia [Ministry of Health Malaysia Press Statement on 22 April 2020, Updates On The Coronavirus Disease 2019 (COVID-19) Situation In Malaysia] [press release]. Malaysia: Ministry of Health, 22 April 2020.

189. Kenyataan Akhbar KPK 24 April 2020 – Situasi Semasa Jangkitan Penyakit Coronavirus 2019 (COVID-19) di Malaysia [Ministry of Health Malaysia Press Statement on 24 April 2020, Updates On The Coronavirus Disease 2019 (COVID-19) Situation In Malaysia] [press release]. Malaysia: Ministry of Health, 24 April 2020.
190. Minister of Health Malaysia. Iklan Pembelian Perkhidmatan Doctor Pakar Swasta dan Doktor Swasta Secara Kontrak Untuk Perkhidmatan (Contract For Service-CFS) Bagi Penyampaian Perkhidmatan Kesihatan Dalam Tempoh Penularan Wabak COVID-19 [Advertisement for the Hiring of Services of Private Specialist and Private Doctors on Contract for Services -CFS for Delivery of Healthcare Service During the Period of COVID-19 Outbreak] Malaysia: Ministry of Health; 2020 [updated 27 April 2020; cited 9 August 2020]. Available from: http://www.moh.gov.my/index.php/database_stores/attach_download/554/41.
191. Social Security Organisation. FAQ Peluasan Skop Program Saringan Prihatin (PSP) PERKESO [SOCSO FAQ on Extension of Scope for Prihatin Screening Programme (PSP)]: Social Security Organisation; 2020 [updated 1 June 2020; cited 7 September 2020]. Available from: https://www.perkeso.gov.my/images/psp/FAQ_SUBSIDI_PSP_PERKESO_010620_FINAL.pdf.
192. Bernama. Sultan of Kelantan contributes two million rubber gloves to MOH. 2020 28 April 2020.
193. New Straits Times. 125 Malaysians fly home. New Straits Times. 2020 30 April 2020.
194. New Straits Times. CMCO: With few exceptions, foreign travellers still barred from entering country. New Straits Times. 2020 1 May 2020.
195. Prime Minister's Office of Malaysia. Teks Ucapan YAB Tan Sri Haji Muhyiddin bin Haji Mohd Yassin Perdana Menteri Malaysia 1 Mei 2020 [Speech Text YAB Tan Sri Dato' Haji Muhyiddin bin Haji Mohd Yassin Prime Ministry of Malaysia 1 May 2020] Malaysia: Prime Minister's Office; 2020 [updated 1 May 2020; cited 23 August 2020]. Available from: https://www.pmo.gov.my/2020/05/perutusan-khas-perdana-menteri-sempena-hari-pekerja-2020/.
196. Kenyataan Akhbar KPK 2 Mei 2020 – Situasi Semasa Jangkitan Penyakit Coronavirus 2019 (COVID-19) di Malaysia [Ministry of Health Malaysia Press Statement on 2 May 2020 - Updates On The Coronavirus Disease 2019 (COVID-19) Situation In Malaysia] [press release]. Malaysia: Ministry of Health Malaysia, 2 May 2020.
197. Kenyataan Akhbar KPK 4 Mei 2020 -Situasi Semasa Jangkitan Penyakit Coronavirus 2019 (COVID-19) di Malaysia [Ministry of Health Malaysia Press Statement on 4 May 2020, Updates On The Coronavirus Disease 2019 (COVID-19) Situation In Malaysia] [press release]. Malaysia: Ministry of Health, 04 May 2020.
198. Minderjeet K. Sungai Buloh Hospital to become centre for infectious diseases. Free Malaysia Today. 2020 5 May 2020.
199. Bunyan J. Gerak Malaysia, MySejahtera, MyTrace: Apps to get you through the MCO. 2020 5 May 2020.
200. CodeBlue. Administrative MCO will be enforced in areas with a sudden increase of Covid-19 cases, and EMCO if there are more infections: Codeblue; 2020 [updated 6 May 2020; cited 18 September 2020]. Available from: https://codeblue.galencentre.org/2020/04/24/moh-sends-socso-covid-19-testing-sop-for-workers/.
201. PGCare. FAQ Malaysia: PGCare; 2020 [updated 31 August 2020; cited 28 September 2020]. Available from: https://pgcare.my/faq.
202. Kenyataan Akhbar KPK 10 Mei 2020 – Situasi Semasa Jangkitan Penyakit Coronavirus 2019 (COVID-19) di Malaysia [Ministry of Health Malaysia Press Statement on 10 May 2020 - Updates On The Coronavirus Disease 2019 (COVID-19) Situation In Malaysia] [press release]. Malaysia: Ministry of Health Malaysia, 10 May 2020.

203. Prime Minister’s Office of Malaysia. Teks Ucapan YAB Tan Sri Haji Muhyiddin bin Haji Mohd Yassin Perdana Menteri Malaysia 10 Mei 2020 [Speech Text YAB Tan Sri Dato' Haji Muhyiddin bin Haji Mohd Yassin Prime Ministry of Malaysia 10 May 2020] Malaysia: Prime Minister’s Office; 2020 [updated 10 May 2020; cited 25 September 2020]. Available from: https://www.pmo.gov.my/wp-content/uploads/2020/06/Teks-Perutusan-YAB-PM-PKPP-07062020.pdf.
204. Ministry of Health Malaysia. Health Screening Booth and Screening & Triaging Services For COVID-19 Malaysia: Ministry of Health Malaysia; 2020 [updated 5 July 2020; cited 18 September 2020]. Available from: http://medicalprac.moh.gov.my/v2/modules/mastop_publish/?tac=Senarai%20KPJKS%20yang%20telah%20diluluskan%20bagi%20COVID-19.
205. Kenyataan Akhbar KPK 20 Mei 2020 – Situasi Semasa Jangkitan Penyakit Coronavirus 2019 (COVID-19) di Malaysia [Ministry of Health Malaysia Press Statement on 20 May 2020 - Updates On The Coronavirus Disease 2019 (COVID-19) Situation In Malaysia] [press release]. Malaysia: Ministry of Health Malaysia, 20 May 2020.
206. Kenyataan Akhbar KPK 22 Mei 2020 – Situasi Semasa Jangkitan Penyakit Coronavirus 2019 (COVID-19) di Malaysia [Ministry of Health Malaysia Press Statement on 22 May 2020 - Updates On The Coronavirus Disease 2019 (COVID-19) Situation In Malaysia] [press release]. Malaysia: Ministry of Health Malaysia, 22 May 2020.
207. Povera A, Yunus A. Govt concerned about immigrants entering M'sia via illegal routes after Raya period. New Straits Times. 2020 28 May 2020.
208. Kenyataan Akhbar KPK 29 Mei 2020 – Situasi Semasa Jangkitan Penyakit Coronavirus 2019 (COVID-19) di Malaysia [Ministry of Health Malaysia Press Statement on 29 May 2020 - Updates On The Coronavirus Disease 2019 (COVID-19) Situation In Malaysia] [press release]. Malaysia: Ministry of Health, 29 May 2020.
209. New Straits Times. Semi-EMCO: Residents at three Kuala Langat neighbourhoods free to move about. New Straits Times. 2020 3 June 2020.
210. Ida Nadirah I. SELangkah merges with Malaysia’s MySejahtera app, says Selangor MB Malaysia: Media Selangor; 2020 [updated 5 June 2020; cited 14 August 2020]. Available from: https://selangorjournal.my/2020/06/selangkah-merges-with-malaysias-mysejahtera-app-says-selangor-mb/.
211. Prime Minister’s Office of Malaysia. Teks Ucapan YAB Tan Sri Haji Muhyiddin bin Haji Mohd Yassin Perdana Menteri Malaysia Pelaksanaan Perintah Kawalan Pergerakan Pemulihan (PKPP) 7 Jun 2020 (Ahad) [Speech Text YAB Tan Sri Dato' Haji Muhyiddin bin Haji Mohd Yassin Prime Ministry of Malaysia Implementation of Recovery Movement Control Order (RMCO) 7 June (Sunday)] Malaysia: Prime Minister’s Office; 2020 [updated 7 Jun 2020; cited 25 September 2020]. Available from: https://www.pmo.gov.my/wp-content/uploads/2020/06/Teks-Perutusan-YAB-PM-PKPP-07062020.pdf.
212. Kenyataan Akhbar KPK 7 Jun 2020 – Situasi Semasa Jangkitan Penyakit Coronavirus 2019 (COVID-19) di Malaysia [Ministry of Health Malaysia Press Statement on 7 June 2020 - Updates On The Coronavirus Disease 2019 (COVID-19) Situation In Malaysia] [press release]. Malaysia: Ministry of Health, 27 June 2020.
213. Kenyataan Akhbar KPK 27 Jun 2020 – Situasi Semasa Jangkitan Penyakit Coronavirus 2019 (COVID-19) di Malaysia [Ministry of Health Malaysia Press Statement on 27 June 2020 - Updates On The Coronavirus Disease 2019 (COVID-19) Situation In Malaysia] [press release]. Malaysia: Ministry of Health, 27 June 2020.

214. Kenyataan Akhbar KPK 15 Jun 2020 – Situasi Semasa Jangkitan Penyakit Coronavirus 2019 (COVID-19) di Malaysia [Ministry of Health Malaysia Press Statement on 15 June 2020 - Updates On The Coronavirus Disease 2019 (COVID-19) Situation In Malaysia] [press release]. Malaysia: Ministry of Health, 15 June 2020.
215. Kenyataan Akhbar KPK 19 June 2020 – Situasi Semasa Jangkitan Penyakit Coronavirus 2019 (COVID-19) di Malaysia [Ministry of Health Malaysia Press Statement on 19 Jun 2020, Updates On The Coronavirus Disease 2019 (COVID-19) Situation In Malaysia] [press release]. Malaysia: Ministry of Health, 19 June 2020.
216. Minister of Health Malaysia. Annex 27a Tindakan Pencegahan, Kawalan Infeksi dan Penggunaan Personal Protective Equipment (PPE) di Fasiliti Penjara, Lokap, Depot Imigresen, PUSPEN dan Rumah Perlindungan [Annex 27a Infection Prevention and Control and Usage of Personal Protective Equipment (PPE) at Prisons, Lockups, Immigration Depots, PUSPEN and Shelter Homes]: Minister of Health Malaysia; 2020 [updated 26 June 2020; cited 8 October 2020]. Available from: http://covid-19.moh.gov.my/garis-panduan/garis-panduan-kkm/Annex_27a_SOP_IPC_Pusat_Tahanan_&_Rumah_Perlindungan.pdf.
217. Ministry of Health Malaysia. Jumlah Keseluruhan Taburan Kes COVID-19 Di Malaysia Sehingga 30 Jun 2020, 12 tengah hari [Total COVID-19 Case Distribution in Malaysia as of 12 noon, 30 June 2020] Malaysia2020 [updated 30 June 2020; cited 5 October 2020]. Available from: http://covid-19.moh.gov.my/user/pages/02.terkini/062020/situasi-terkini-30-jun-2020/taburankes.jpg.
218. Department of Statistics Malaysia. Principal Statistic - Malaysia 2nd Quarter 2020 2020 [updated 30 June 2020; cited 9 October 2020]. Available from: https://www.dosm.gov.my/.
219. Yusof ANM, Muuti MZ, Ariffin LA, Tan MKM. Sharing Information on COVID-19: the ethical challenges in the Malaysian setting. Asian bioethics review [Internet]. 2020 5 July 2020 8 August 2020]:[1-13 pp.].
220. Khoo LS, Hasmi AH, Ibrahim MA, Mahmood MS. Management of the dead during COVID-19 outbreak in Malaysia. Forensic Sci Med Pathol [Internet]. 2020 5 July 2020 7 August 2020]; 16(3):[463-70 pp.]. Available from: https://pubmed.ncbi.nlm.nih.gov/32519316

https://www.ncbi.nlm.nih.gov/pmc/articles/PMC7280680/.
221. Tang KHD. Movement control as an effective measure against Covid-19 spread in Malaysia: an overview. Z Gesundh Wiss [Internet]. 2020 5 July 2020 8 August 2020]:[1-4 pp.]. Available from: https://pubmed.ncbi.nlm.nih.gov/32837842

https://www.ncbi.nlm.nih.gov/pmc/articles/PMC7293423/.
222. Umair S, Waqas U, Faheem M. COVID-19 pandemic: stringent measures of Malaysia and implications for other countries. Postgraduate medical journal [Internet]. 2020 5 July 2020 9 August 2020].
223. Elengoe A. COVID-19 Outbreak in Malaysia. Osong public health and research perspectives [Internet]. 2020 5 July 2020 6 August 2020]; 11(3):[93-100 pp.].
224. Yan Y, Shin WI, Pang YX, Meng Y, Lai J, You C, et al. The First 75 Days of Novel Coronavirus (SARS-CoV-2) Outbreak: Recent Advances, Prevention, and Treatment. Int J Environ Res Public Health [Internet]. 2020 5 July 2020 7 August 2020]; 17(7).
225. Tan CLH, Huei TJ, Mohamad Y, Alwi RI, Tuan Mat TNA. Critical adjustments and trauma surgery trends in adaptation to COVID-19 pandemic in Malaysia. Chin J Traumatol [Internet]. 2020 5 July 2020 7 August 2020]; 23(4):[207-10 pp.]. Available from: https://pubmed.ncbi.nlm.nih.gov/32653358

https://www.ncbi.nlm.nih.gov/pmc/articles/PMC7255157/.
226. Tay K, Kamarul T, Woo YL, Mansor M, Li X, Wong J, et al. COVID-19 in Singapore and Malaysia: Rising to the Challenges of Orthopaedic Practice in an Evolving Pandemic. Malays Orthop J [Internet]. 2020 5 July 2020 9 August 2020]; 14(2):[7-15 pp.]. Available from: https://www.ncbi.nlm.nih.gov/pmc/articles/PMC7169474/.
227. Cheong MWL, Brock T, Karwa R, Pastakia S. COVID-19 and Clinical Pharmacy Worldwide - A Wake Up Call and a Call to Action. J Am Coll Clin Pharm [Internet]. 2020 5 July 2020 8 August 2020]:[10.1002/jac5.286 p.]. Available from: https://pubmed.ncbi.nlm.nih.gov/32838222

https://www.ncbi.nlm.nih.gov/pmc/articles/PMC7323236/.
228. Shah AUM, Safri SNA, Thevadas R, Noordin NK, Rahman AA, Sekawi Z, et al. COVID-19 outbreak in Malaysia: Actions taken by the Malaysian government. International Journal of Infectious Diseases [Internet]. 2020 5 July 2020 8 August 2020]; 97:[108-16 pp.]. Available from: http://www.sciencedirect.com/science/article/pii/S1201971220304008.
